# Time-Series Modeling and Forecasting of Cerebral Pressure–Flow Physiology: A Scoping Systematic Review of the Human and Animal Literature

**DOI:** 10.3390/s24051453

**Published:** 2024-02-23

**Authors:** Nuray Vakitbilir, Logan Froese, Alwyn Gomez, Amanjyot Singh Sainbhi, Kevin Y. Stein, Abrar Islam, Tobias J. G. Bergmann, Izabella Marquez, Fiorella Amenta, Younis Ibrahim, Frederick A. Zeiler

**Affiliations:** 1 Biomedical Engineering, Price Faculty of Engineering, University of Manitoba, Winnipeg, MB R3T 5V6, Canada; froesel3@myumanitoba.ca (L.F.); amanjyot.s.sainbhi@gmail.com (A.S.S.); steink34@myumanitoba.ca (K.Y.S.); islama9@myumanitoba.ca (A.I.); frederick.zeiler@umanitoba.ca (F.A.Z.); 2 Section of Neurosurgery, Department of Surgery, Rady Faculty of Health Sciences, University of Manitoba, Winnipeg, MB R3A 1R9, Canada; gomeza35@myumanitoba.ca (A.G.); younis.ibrahim@umanitoba.ca (Y.I.); 3 Department of Human Anatomy and Cell Science, Rady Faculty of Health Sciences, University of Manitoba, Winnipeg, MB R3E 0J9, Canada; 4 Undergraduate Engineering, Price Faculty of Engineering, University of Manitoba, Winnipeg, MB R3T 5V6, Canada; bergmant@myumanitoba.ca (T.J.G.B.); marquezi@myumanitoba.ca (I.M.); amentaf@myumanitoba.ca (F.A.); 5 Department of Clinical Neuroscience, Karolinska Institutet, 171 77 Stockholm, Sweden; 6 Division of Anesthesia, Department of Medicine, Addenbrooke’s Hospital, University of Cambridge, Cambridge CB2 0QQ, UK

**Keywords:** cerebral physiologic signal analysis, cerebral pressure–flow dynamics, time-series modeling, time-series forecasting

## Abstract

The modeling and forecasting of cerebral pressure–flow dynamics in the time–frequency domain have promising implications for veterinary and human life sciences research, enhancing clinical care by predicting cerebral blood flow (CBF)/perfusion, nutrient delivery, and intracranial pressure (ICP)/compliance behavior in advance. Despite its potential, the literature lacks coherence regarding the optimal model type, structure, data streams, and performance. This systematic scoping review comprehensively examines the current landscape of cerebral physiological time-series modeling and forecasting. It focuses on temporally resolved cerebral pressure–flow and oxygen delivery data streams obtained from invasive/non-invasive cerebral sensors. A thorough search of databases identified 88 studies for evaluation, covering diverse cerebral physiologic signals from healthy volunteers, patients with various conditions, and animal subjects. Methodologies range from traditional statistical time-series analysis to innovative machine learning algorithms. A total of 30 studies in healthy cohorts and 23 studies in patient cohorts with traumatic brain injury (TBI) concentrated on modeling CBFv and predicting ICP, respectively. Animal studies exclusively analyzed CBF/CBFv. Of the 88 studies, 65 predominantly used traditional statistical time-series analysis, with transfer function analysis (TFA), wavelet analysis, and autoregressive (AR) models being prominent. Among machine learning algorithms, support vector machine (SVM) was widely utilized, and decision trees showed promise, especially in ICP prediction. Nonlinear models and multi-input models were prevalent, emphasizing the significance of multivariate modeling and forecasting. This review clarifies knowledge gaps and sets the stage for future research to advance cerebral physiologic signal analysis, benefiting neurocritical care applications.

## 1. Introduction

Cerebral physiologic signals serve as windows into the complex neurophysiological processes of the brain. These signals not only provide essential insights into cerebral dynamics but also hold critical clinical implications for both humans and veterinary cohorts, particularly in the realm of neurocritical care [1,2]. Understanding the relationships within and among these signals is paramount for accurate diagnosis, monitoring, and therapeutic intervention in patients with neurological disorders [3]. Of particular importance is being able to understand the temporal behavior, and potentially forecast or predict various aspects of cerebral pressure–flow and oxygen delivery metrics, as these aspects are potentially modifiable in real-time in both the health sciences and veterinary fields. Additionally, although further research is needed, it is crucial to highlight the potential relationship between cerebral pressure–flow and certain neurological conditions, such as brain tumors, Alzheimer’s disease, and Parkinson’s disease [4,5]. Such cerebral pressure–flow metrics are derived from a combination of invasive/non-invasive cerebral monitoring devices, providing high-frequency continuous data streams related to intracranial pressure (ICP), cerebral perfusion pressure (CPP), cerebral blood flow (CBF) and CBF velocity (CBFv; acquired through transcranial Doppler (TCD)), cerebral autoregulation (CA), extracellular brain tissue oxygen (PbtO_2_), and regional oxygen saturations (rSO_2_; using near-infrared spectroscopy (NIRS)) [6,7]. Typically, continuous waveforms of ICP, CPP, CBF, and PbtO_2_ are acquired through invasive sensors placed directly into the cranial cavity and brain parenchyma. CBFv and NIRS signals can be obtained continuously in a non-invasive pattern using Doppler ultrasound probes to insonate the middle cerebral artery (i.e., CBFv), or using spatially resolved continuous-wave NIRS signal sources for oxy- and deoxyhemoglobin signals or processed rSO_2_ values. CA metrics carry the unique nature of being able to be derived from raw physiologic data streams from either invasive (i.e., ICP) or non-invasive (i.e., TCD or NIRS) sensor sources.

There are several time-series analysis techniques used for the examination and modeling of cerebral physiologic signals that bridge frequency domain, time domain, and machine learning methods. Regarding the temporal modeling of such cerebral pressure–flow data streams, however, the literature on this topic remains scattered, with various approaches including both linear and nonlinear methods using statistical analysis techniques such as time domain and frequency domain analyses, having been developed to assess the pressure–flow relation for dynamic CA modeling [8]. Similarly, work including transfer function analysis (TFA), the continuous wavelet transform (CWT), empirical mode decomposition (EMD), fast Fourier transform (FFT), cross-spectral analysis, wavelet analysis, Granger causality, autoregressive (AR) models, and so on [9] have been described. In addition to the statistical time-series analysis techniques, there are various machine learning algorithms used for cerebral physiologic signal modeling [10,11] as well as for the prediction task [12], including models such as linear regression, artificial neural networks (ANNs), convolutional neural networks (CNNs), extreme gradient boosting (XGBoost), and decision trees. These algorithms offer adaptability and data-driven capabilities that can uncover intricate patterns within the data, particularly in cases where complexities demand more flexible modeling approaches [13]. The juxtaposition of these two approaches, i.e., statistical time-series analysis techniques (leveraging frequency or time domain methods) and machine learning algorithms, presents a compelling landscape for the temporal analysis and forecasting of cerebral pressure–flow physiologic signals.

This systematic scoping review aims to comprehensively explore and provide a synthesis and evaluation of the literature on temporally resolved cerebral pressure–flow physiologic data modeling and forecasting/prediction. We aim to provide insights into the methodologies employed, current knowledge, key findings, research gaps, limitations, and implications for future research. Our goal is to shed light on the evolving landscape of cerebral pressure–flow physiologic signal analysis and modeling, ultimately contributing to improved fundamental physiological understanding.

## 2. Materials and Methods

The methodology outlined in the Cochrane Handbook for Systematic Reviews [14] was followed as guidance for this systematic scoping review of the available literature. Our reporting adhered to the guidelines provided by the Preferred Reporting Items for Systematic Reviews and Meta-Analysis (PRISMA) [15] and PRISMA Extension for Scoping Review [16]. The methodology and search approach employed in this review closely align with previous systematic reviews carried out by our research team [17,18]. The formulation of review objectives and the design of the search strategy were a collaborative effort involving the primary (NV, LF) and senior (FZ) authors.

### 2.1. Search Questions, Population, and Inclusion/Exclusion Criteria

In this systematic scoping review, we examined the following question: What cerebral pressure–flow physiology has been modeled or predicted/forecasted using high-temporal time-series methods?

For the purposes of this scoping review, we defined continuous pressure–flow physiologic data streams as those from either invasive or non-invasive sensors, being recorded at a minimum of 0.1 Hertz (Hz), and measuring some aspect of ICP, CPP, CBF, CBFv, CA, PbtO_2_, rSO_2_, or cerebral compliance. We are including English language full-manuscript studies only, which studies human or animal subjects in states of health or disease. Given the primary focus of our review centered on the modeling or prediction of cerebral pressure–flow physiological time-series data, all included studies had to describe modeling or prediction/forecasting of cerebral pressure–flow signals in time. Finally, any study that leveraged time-series analytic (time and/or frequency domain) or machine learning methods to derive these temporally resolved models was included. This included the following modeling methodologies: cross-spectral analysis; Welch method; multiple coherence estimation; TFA; FFT; power-spectrum analysis; CWT; wavelet analysis; discrete-time Laguerre function model; principal dynamic modes (PDMs); linear Laguerre-based model; Laguerre–Volterra network (LVN) model; Volterra–Wiener method; Aaslid–Tiecks model; Zhao–Atlas–Marks distribution (ZAMD); moving correlation coefficient; single pulse analysis; dynamical Bayesian inference (DBI); Granger causality; generalized harmonic wavelets (GHWs); nonparametric transfer function estimator; Laguerre expansion technique (LET); autoregressive moving average (ARMA); autoregressive with exogenous input (ARX); autoregressive moving average with exogenous input (ARMAX); autoregressive integrative moving average (ARIMA); autoregressive ordinal-regression (AR-OR); vector autoregressive (VAR); vector autoregressive integrative moving average (VARIMA); ANN; hidden Markov model (HMM); k-nearest neighbor (k-NN) algorithm; lasso regression; linear regression; logistic regression; time-varying temporal signal angle measurement (TSAM) algorithm; time-lagged recurrent neural network (TLRN); SVM; wavelet-based k-means clustering; forecasting with additive switching of seasonality, trend, and exogenous regressors (FASSTER); time-varying dynamic linear models (DLMs); fractal analysis with box-counting and Higuchi algorithms; random forest; exponential smoothing (ETS) model; intrinsic multiscale pressure–flow analysis (IMPFA); multimodal pressure–flow analysis (MMPF); XGBoost; light gradient boosting model (LGBM); adaptive boosting (AdaBoost); extremely randomized decision trees (ExtraTrees); robust time-varying generalized partial directed coherence with the Kalman filter; dual extended Kalman filter (DEKF); short-time Fourier transform (STFT); Kalman filtering; recurrent neural network (RNN); multiresolution dynamic predictor (MDP); probabilistic Markov model; linear mixed effects (LME); and Mandeville’s visco-elastic Windkessel (VM) and elastic Windkessel (EW) models.

Non-English studies, as well as organoid and purely theoretical studies, were excluded, as these did not align with our aim to focus on empirical research. Additionally, studies involving non-continuous data streams, such as MRI studies, were deemed outside the scope of this review. Similarly, purely electroencephalography (EEG) data studies were excluded, as the focus was on cerebral pressure–flow physiologic data steams (as described above). Furthermore, non-original studies and abstract-only studies were intentionally omitted to ensure the inclusion of substantive research contributions in our analysis.

### 2.2. Search Strategy

We conducted comprehensive searches across multiple databases, namely BIOSIS, Cochrane Library, EMBASE, MEDLINE, and SCOPUS, covering the entire period from the inception of each database up to mid-March 2023 using tailored search strategies for each database to ensure precision. A detailed outline of the search strategy for BIOSIS, along with the specific keywords employed, can be found in Supplementary Appendix SA. Following the retrieval of search results from these sources, we merged the findings and conducted a meticulous deduplication process.

### 2.3. Study Selections

Utilizing a two-reviewer approach, involving NV and LF, we conducted a meticulous two-stage manual review of all articles yielded by the search strategy. In the initial filtering phase, both reviewers independently assessed all identified studies using the search strategy described earlier, evaluating their eligibility based on the title and abstract. The resulting list of selected studies then proceeded to a second filtering phase, where, once again, both reviewers independently assessed the studies for inclusion, this time based on a full-text examination. In the event of any discrepancies between the two reviewers, a third-party mediator (FZ) was consulted for resolution. Additionally, for any conference abstracts identified during this process, we diligently attempted to locate associated peer-reviewed manuscripts for potential inclusion. To further ensure the comprehensiveness of our review, we conducted a thorough examination of the reference lists, of the articles reviewed, on time-series analysis.

### 2.4. Data Collection

The data fields encompassed various study subject characteristics, including biological sex, age, height, weight, cerebral physiology, as well as other physiological parameters and their respective measurement methods. Additionally, we extracted information about data resolution, the approaches employed for time-series modeling or prediction, any comparative analyses of models, primary objectives, and the relevant findings and conclusions from the studies.

### 2.5. Bias Assessment

Considering the objective of this review, to provide a thorough and broad survey of the literature, we did not undertake a formal bias assessment.

### 2.6. Statistical Analysis

Meta-analysis was omitted from this study, given the extensive heterogeneity in study designs and outcomes within the relevant literature.

## 3. Results

**Figure 1:**
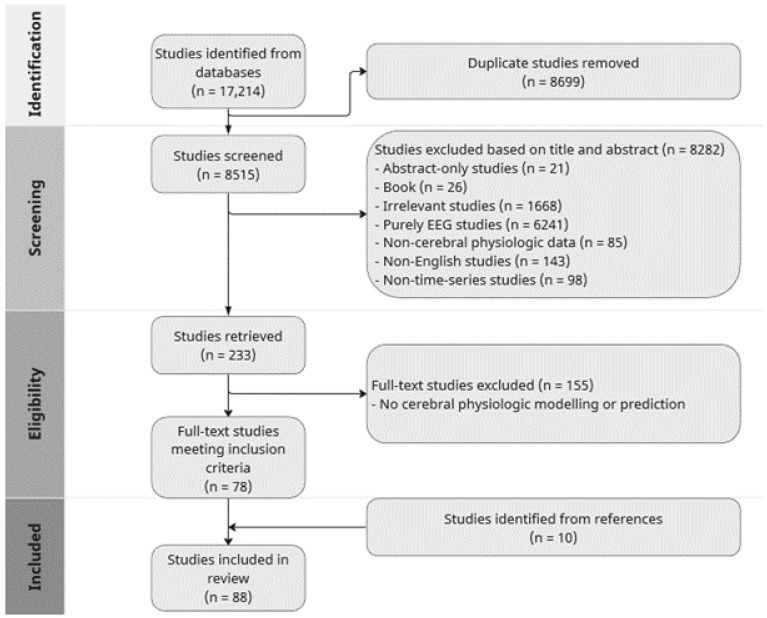
PRISMA flow diagram of this systematic review.

The search and filtration results are summarized in Figure 1 using a PRISMA flow-diagram. A total of 17,214 studies were identified through the combined search across all five databases, of which 8699 were removed as duplicates. During the screening process, 8282 studies were deemed unsuitable based on their titles and abstracts, in accordance with the inclusion/exclusion criteria. Consequently, 233 studies were extracted for full-text review in the subsequent phase. The full-text review led to the exclusion of 155 studies that were outside the scope of modeling or predicting cerebral physiology, resulting in 78 eligible studies for inclusion. Furthermore, a supplementary exploration of the reference sections within those texts led to the identification of 10 additional studies, resulting in a total of 88 studies incorporated into this systematic review. Details of the included studies can be found in Supplementary Appendix SB, in Tables S1–S8.

**Figure 2:**
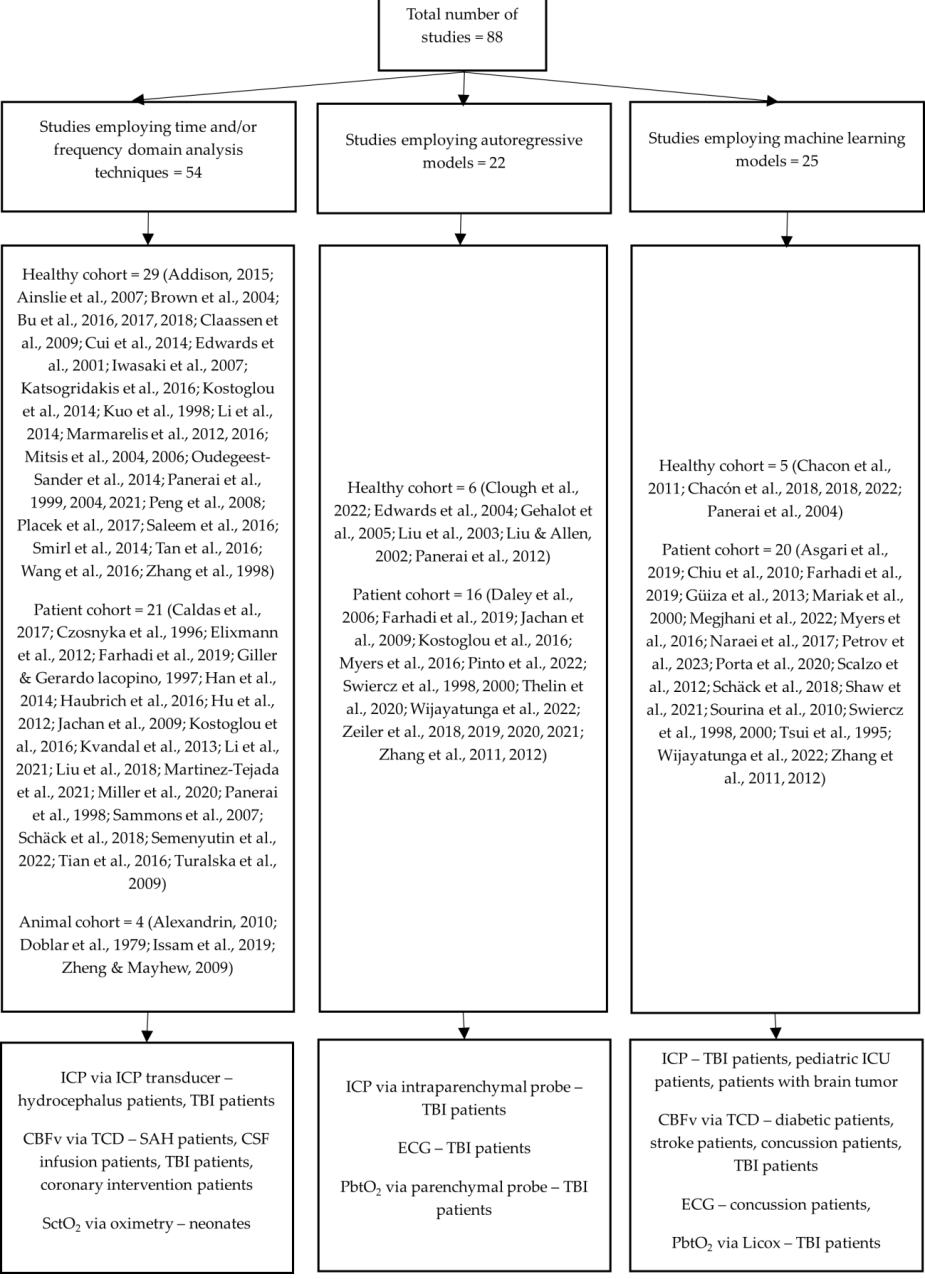
Distribution of studies corresponding to the methodologies employed as well as the medical diagnostic tests with respect to studied pathology.

In this systematic review, 38 of the included studies investigated healthy population data [1,13,19,20,21,22,23,24,25,26,27,28,29,30,31,32,33,34,35,36,37,38,39,40,41,42,43,44,45,46,47,48,49,50,51,52,53,54], 46 studied patient populations [2,3,55,56,57,58,59,60,61,62,63,64,65,66,67,68,69,70,71,72,73,74,75,76,77,78,79,80,81,82,83,84,85,86,87,88,89,90,91,92,93,94,95,96,97,98], while the remaining 4 studies focused on animal subjects [99,100,101,102]. Figure 2 illustrates the distribution of studies based on the methods employed in their research as well as the medical diagnostic tests with respect to the studied pathology. Please note that the studies conducting comparisons may have been referenced multiple times due to the various methodologies utilized.

The extensive summaries of healthy population studies are listed in Tables S1–S3, whereas studies with patient cohorts are presented in Tables S4–S7 and animal studies in Table S8 in Supplementary Appendix SB. In the sections to follow, we outline the ability of the time and/or frequency domain, and machine learning methods to model and/or predict the above-defined continuous cerebral pressure–flow physiologic metrics of interest in the following sections: (1) healthy human populations, (2) human patient populations, and (3) animal cohorts.

### 3.1. Healthy Population—General Study Characteristics and Modeling Methods

Among the studies involving healthy populations, 20 studies [1,19,20,21,22,23,26,28,32,33,35,36,43,47,48,50,51,52,53,54] used time-series analysis techniques composed of frequency-domain analysis methods, TFA, and wavelet analysis; 6 studies [27,30,31,37,38,46] used AR time-series models, namely ARMA and ARX; and 12 studies [13,24,25,29,34,39,40,41,42,44,45,49] employed multiple modeling techniques, including time and/or frequency domain techniques and machine learning models, for comparative analysis at various frequency ranges, i.e., very low frequency (VLF), low frequency (LF), and high frequency (HF). These studies, listed in Table 1, are categorized based on the modeled or forecasted cerebral physiologic signals and the corresponding modeling techniques, including a comment on their ability to model or predict.

In most of the studies, the study population consisted primarily of healthy young adult volunteers who were free from documented cardiovascular or neurological diseases, with an average age of approximately 30 years. However, there were exceptions, as some studies [23,28,36,43,52,53] included both young and elderly healthy adults, and one study [51] encompassed healthy volunteers, heart transplant recipients, and donor controls.

In most of the studies, the data were resampled from very high resolution to low resolution ranging from 1 Hz to 5 Hz. A significant portion of the studies recorded CBFv using a transcranial Doppler probe targeting the middle cerebral artery (MCA); however, some studies only recorded rSO_2_ [1] or the change in oxyhemoglobin concentration (Δ[HbO]) using NIRS [21,22,23,28,36,52,53]. Two studies recorded both CBFv using TCD and Δ[HbO] using NIRS [43]. Additionally, in one study [19], the change in deoxyhemoglobin concentration (Δ[Hb]) and change in total hemoglobin (Δ[HbTot]) using NIRS were recorded in addition to Δ[HbO] and CBFv. Arterial blood pressure (ABP) and end-tidal carbon dioxide (EtCO_2_) were also recorded in the majority of the studies, except for a select few that recorded only ABP [1,25,28,31,35,38,44,45], only EtCO_2_ [29], or neither [21,22,23,36,52,53]. Some studies estimated the resistance-area product (P_RA_) and critical closing pressure (CrCP) from CBFv and ABP [27,33,47,56].

#### 3.1.1. Time and/or Frequency Domain Modeling Techniques

The studies, which employed time and/or frequency domain models with a healthy subject cohort, mainly modeled CBFv [19,20,26,32,33,35,43,47,48,50,51,54] or Δ[HbO] [19,21,22,23,28,36,52,53] signals, while one study examined the rSO_2_ signal [1]. Overall, the study results reported the successful modeling of these cerebral physiologic signals discovering the underlying patterns, in many cases to assess CA [20,33,34,39,40,47,49,54]. Due to the nature of data collection from healthy volunteers, ICP and PbtO_2_ signals were not modeled in any of the studies included in this section. Additionally, forecasting of the cerebral physiologic signals was not carried out.

Cross-spectral analysis, which is used to examine the connection between two time series with respect to their frequency dependence [104], was utilized in two studies ([20,35], *p*-value < 0.01). Welch’s method, which is used for estimation of the power spectral density of a signal, was utilized in one study ([33], *p*-value < 10^−4^). Two studies [33,48] utilized multiple coherence estimation, which analyzes the degree of coherence or correlation between a reference signal and multiple other signals at various frequencies. Power spectrum analysis, which is used for understanding the frequency components present in a signal and their relative amplitudes, was employed in one study [51]. TFA, which is a mathematical representation of a relation between an input and output of a linear, time-invariant system [105], was utilized by five studies to rigorously evaluate the dynamic relationship between ABP and CBFv as a result of repeated squat–stand maneuvers ([26,51], *p*-value < 0.01), hypoxia ([32], *p*-value < 0.05), hypercapnia [47,54], and the placebo effect ([50], *p*-value < 0.05). In two other studies, TFA was used to analyze the relation between CBFv and various signals, i.e., Δ[HbO] and EtCO_2_ ([19], *p*-value < 0.05), and ABP and EtCO_2_ ([43], *p*-value < 0.05).

Wavelet analysis is used for analyzing signals in the time–frequency domain [106]. One study [1] utilized the synchro-squeezed CWT (synchro-CWT) model, which is an advanced wavelet analysis model for improved time–frequency analysis and cross-frequency interaction assessment in signals [107], to analyze the coupling dynamics between ABP and rSO_2_. Seven studies employed wavelet analysis to investigate phase synchronization patterns within Δ[HbO] signals, observing varying wavelet amplitude, wavelet coherence (WCO), and wavelet phase coherence (WPCO) in different frequency ranges as a result of long-term offshore work ([21], *p*-value < 0.04), sleep deprivation ([22,23], *p*-value < 0.04 in both studies), and aging ([28], *p*-value < 0.03; [36], *p*-value < 0.014; [52], *p*-value < 0.03; [53], *p*-value < 0.05). Another study [50] analyzed cerebral pressure–flow relations with wavelet analysis (*p*-value < 0.05).

#### 3.1.2. Autoregressive Modeling Techniques

The studies employing various AR models reported the successful modeling of the CBFv signal, several of which assessed CA [27,30,31,38]. Similar to time–frequency domain techniques, ICP and PbtO_2_ signals were not modeled, and forecasting of the cerebral physiologic signals was not carried out.

These AR time-series models included ARMA models which is a statistical model capable of analyzing and forecasting the behavior of time-series data by combining AR and moving average (MA) components, and ARX models which represent dynamic systems with dependencies on both past values and external input signals. Among these, three studies utilized ARMA for the analysis of dynamic CA under paced hyperventilation (PHPV) ([27], *p*-value = 0.003) and hypercapnia [30], and to examine CBFv response during motor stimulation ([46], *p*-value < 0.03). Another three studies employed ARX to model CA during rest ([31], *p*-value < 0.3 between 1.5 min and 6 min datasets, *p*-value ranging from 0.54 to 0.88 between 1.5 min datasets), under noisy conditions ([38]; 5-s recovery percentage (R5%) = 92–97 ± 8 depending on noise and variation in ABP), and to assess CBFv under normocapnia and hypercapnia ([37], *p*-value < 0.001).

#### 3.1.3. Model Comparison Studies

The majority of the model comparison studies [29,34,39,40,41,42,44,45,49] employed time and/or frequency domain models, while a few [13,24,25,45] compared machine learning algorithms in their performance to model CBFv signals. As mentioned in the previous sections, other cerebral physiologic signals such as ICP and PbtO_2_ were not collected from the subject cohort. In the studies under comparative model evaluation, various types of time-series analysis techniques and machine learning algorithms were employed. The objective of the studies was mainly to compare the modeling performance of linear models and nonlinear models as well as compare the effect of input size. All employed models were suggested to offer the ability to model CBFv signals to an extent. However, better modeling performances were observed with linear models and multiple-input models. Additionally, better modeling was reported with machine learning models compared to time/frequency domain models [13,45].

SVM, which is a supervised machine learning algorithm used for the classification or modeling of time-series data, was utilized by three studies to assess cerebrovascular reactivity (CVR) [24] and dynamic CA [13,25] by comparing linear and nonlinear models. Chacon et al. compared linear AR SVM, nonlinear AR (NAR) SVM, linear finite impulse response (FIR) SVM, and nonlinear FIR (NFIR) SVM models for modeling CBFv ([24], *p*-value < 0.002 with AR models). Another study compared TFA, NAR SVM, and NFIR SVM to model the CBFv response to BP changes ([13], *p*-value < 0.001 with nonlinear SVM models). Furthermore, another comparative study by Chacon et al. compared performances of FIR SVM, NFIR SVM, nonlinear ARX (NARX) SVM, and ARX SVM models for assessing CBF [25]. Panerai et al. compared the performance of the Volterra–Wiener method to the FFT and Aaslid–Tiecks model, which is a mathematical model that can be used for estimating the step responses from spontaneous fluctuations in ABP and CBFv [108] (*p*-value < 10^−6^) [44]. In their consecutive study, Panerai et al. compared the TLRN model, which is used for modeling sequential data to capture temporal dependencies and patterns, the Aaslid–Tiecks model, the linear Volterra–Wiener method, TFA, and the simple linear regression model for modeling CBFv [45]. Another study conducted a comparative analysis involving the Zhao–Atlas–Marks distribution (ZAMD), which is a distribution function characterized by a cone-shaped kernel, that can be used for CA assessment [109]; TFA; and ARMA models to estimate the phase shift between ABP and CBFv [49].

Additionally, several studies carried out comparative studies between single-input and multiple-input models in addition to the comparison of linearity. Overall multiple-input models achieved better results in all the studies included [30,34,39,40,41,42]. Edwards et al. employed two-input ARMAX, which is an extension of ARMA incorporating exogenous input variables to improve the model’s predictive capability [110], and one-input cross-spectral analysis [30]. Kostoglou et al. compared one-input and two-input discrete-time Laguerre function models, which are capable of representing and analyzing signals and systems in the time, frequency, or Laguerre domain [34]. The PDM model was used in two studies [39,40] to analyze dynamic CA. One study [39] compared linear- and nonlinear-, one- and two-input PDM models to a linear Laguerre-based model, and linear single-input TFA (normalized mean squared error (NMSE) = 40.4% with nonlinear two-input PDM model). In a consecutive study by Marmarelis et al., they focused on PDMs to compare between linear- and nonlinear-, two- and three-input models ([40], *p*-value < 0.005). LVN, which is a type of an artificial neural network for modeling nonlinear dynamic systems [111], was used in two studies to compare between variations of models with linearity (linear and nonlinear) and input size (one- and two-input) [42] as well as different model orders (1st, 2nd, and 3rd) [41] to assess the interactions of various signals on CBFv variations ([41]; NMSE < 33). The Volterra–Wiener method, which is an approach used for estimation of the linear and nonlinear expressions of the dynamic pressure–volume relationship [112], was utilized by two studies to assess the dynamic relationship between ABP and CBFv.

### 3.2. Human Patient Population Studies—General Study Characteristics and Modeling Methods

Similar to healthy cohort studies, the studies involving patient populations have been organized into categories. A total of 14 studies [56,58,60,62,64,65,69,70,71,73,78,82,90,92] utilized time-series analysis techniques, including dynamic relationship analysis methods, TFA, and wavelet analysis. A total of 5 studies [59,80,89,95,96] employed variations in AR time-series models, 8 studies [55,57,72,74,77,81,85,86] utilized machine learning models, and 19 studies [2,3,61,63,66,67,68,75,76,79,83,84,87,88,91,93,94,97,98] carried out comparative model evaluations.

In the majority of the articles, the study population consisted primarily of traumatic brain injury (TBI) patients with various severities, with the exceptions of articles that studied idiopathic normal-pressure hydrocephalus patients [60], SAH patients [62,74], cerebrospinal fluid (CSF) infusion patients [73], coronary intervention patients [56,81,82], pediatric patients [61,78,90], elderly people with cerebral infarction (CI) [64,71], hypertensive individuals [70], diabetic patients [57], intracerebral hemorrhage patients [72,87,88], stroke patients [66], arterial stenosis patients [67,75,84], concussion patients [68], and patients with various ICP-related conditions [3].

The patient cohort studies are divided into TBI and non-TBI patients grouped based on cerebral physiologic signals and the modeling technique, along with a comment on their ability to model or predict, and are listed in Table 2 and Table 3, respectively.

In most of the studies, the data were resampled from very high resolution to low resolution ranging from 0.1 Hz to 5 Hz. Overall, the majority of the studies recorded ICP with either a fiber-optic transducer, a subdural catheter, an intraparenchymal probe, or an external ventricular drain, except for studies that recorded only CBFv via a TCD probe from the MCA [56,57,62,66,67,68,73,75,78,81,82,84], Δ[HbO] and Δ[Hb] using NIRS [64,70,71], or cerebral tissue oxygen saturation (SctO_2_) via oximetry [90]. Some studies estimated CPP from MABP and ICP [2,55,58,59,61,63,74,80,94,95], pressure–volume reserve (RAP) from the pulse amplitude of ICP and ICP data [55,65], and pressure reactivity index (PRx) from ABP and ICP [55,69,74,89]. Some studies also measured continuous PbtO_2_ with an invasive parenchymal probe [76,83,96].

#### 3.2.1. Time and/or Frequency Domain Modeling Techniques

A small portion of the studies employing time and/or frequency domain models with a patient cohort consist of TBI patients [58,65,69,92] and neonates [78,90], while the remaining studies are on individuals with various health issues [56,60,62,64,70,71,73,82]. The studies mainly modeled ICP [58,60,65,69,92], CBFv [56,62,65,73,78,82,92], or Δ[HbO] [64,70,71] signals, while SctO_2_ was modeled in only one study [90]. Several of these studies [56,62,69,78,82,90] aimed to assess CA. Overall, study findings revealed effective modeling of the mentioned cerebral physiologic signals uncovering the intrinsic patterns.

In a study by Czosnyka et al., the moving correlation coefficient was utilized for tracking changes in the correlation between two time series as a function of time, observing a clear correlation between the fundamental harmonic of the ICP pulse wave and the mean ICP for severe TBI patients [58]. Single pulse analysis, which is method of studying and analyzing individual, isolated pulses within a larger data stream, was used in a study by Elixmann et al. to identify ICP signals [60]. Another investigation, Giller and Gerardo Iacopino, utilized FFT, which analyzes and process signals in the frequency domain, to assess the coherence between CBFv and blood pressure (BP) [62]. DBI can detect time-varying dynamics despite noise and track temporal changes in the relevant parameters [70]. Two studies employed DBI to investigate the coupling strength between ABP and oxyhemoglobin concentration (HbO) ([70], *p*-value < 0.02; [71], *p*-value < 0.03). Martinez-Tejada et al. employed the Granger causality method in conjunction with ensemble empirical mode decomposition (EEMD) to explore causal relationships between oscillatory modes of ICP, ABP, and CBFv [73]. TFA was employed by four studies in the patient populations to investigate dynamic CA ([56], *p*-value = 0.052; [65,78], *p*-value < 0.0009; [82], *p*-value < 0.02). Wavelet analysis was used in the remaining four studies to study prefrontal functional connectivity ([64], *p*-value < 0.4), to evaluate CA ([69], *p*-value < 0.05; [90], *p*-value < 0.3), and to analyze CBF ([92], *p*-value < 0.1).

#### 3.2.2. Autoregressive Modeling Techniques

All of the studies [59,80,89,95,96] employing various AR models modeled ICP data along with CPP, and in one study with PbtO_2_. The studies reported effective modeling results overall.

ARMA was utilized in one study [59] to study the impact of ABP and ICP on cerebrovascular pressure transmission. The VARFI model was used in the study by Pinto et al. to examine the interconnections between oscillations in R-R intervals, MABP, and the pulse amplitude of ICP [80]. ARIMA was employed along with VARIMA, and univariate logistic regression and Granger causality were employed in a study by Thelin et al. to examine the statistical time-series relationship between ICP, MABP, and PRx of adult TBI patients [89]. The ARIMA model differs from ARMA with the ability to handle non-stationary in data such as seasonality or trends [113], and VARIMA is used for analyzing multivariate time-series data exhibiting non-stationarity or complex dynamics [114]. Univariate logistic regression is a statistical modeling technique to analyze the relationship between an independent variable and a binary outcome, i.e., dependent variable [115]. Additionally, Granger causality is a method of assessing the causal influences between simultaneously obtained time series [116]. Another study [95] used VARIMA and impulse response function (IRF) analysis to assess the effect of craniectomy on PRx as well as the relationship between vasogenic slow waves of ICP and MABP (*p*-value < 0.3). In a consecutive study, Zeiler et al. employed ARIMA, VARIMA-generated IRF plots, and Granger causality to investigate the relationship between slow-wave fluctuations in ICP, MABP, and PbtO_2_ [96].

#### 3.2.3. Machine Learning Techniques

Eight studies [55,57,72,74,77,81,85,86] employed a range of machine learning algorithms to address diverse objectives. Half of these studies [55,77,85,86] analyzed TBI patient data with ICP and CPP, while the other half examined ICP [72,74] or CBFv [57,81] signals from individuals with various health issues. The studies analyzing ICP signals carried out prediction [55,74,77,85,86] or classification [72] tasks. Overall, two study findings revealed an effective modeling of CBFv signals. The remaining studies reported adequate ICP prediction and stressed the potential of the respective model.

HMM, which is capable of modeling sequential or temporal data and capturing patterns in sequences [117], was employed by Asgari et al. to categorize cerebral dynamic states [55]. Another study [57] employed SVM to extract features with the linear cross-correlation function (CCF) and the nonlinear correlation dimension (CD) to non-invasively classify dynamic CA. In a subsequent investigation, Mariak et al. employed ANN, which learns patterns, classifies data, and makes predictions by making probability-based associations between an input and output, to classify ICP waveforms into risk classes [72]. The TSAM algorithm, which is a method designed to address the challenges associated with analyzing time-varying data, particularly with limited data availability, was used to predict delayed cerebral ischemia (DCI) ([74]; model accuracy = 67.3%). In another study, Naraei et al. applied wavelet-based K-means clustering, which is a unsupervised clustering technique that does not rely on prior knowledge gained from labeled data, to differentiate ICP levels [77]. Porta et al. utilized the k-NN algorithm, which is a machine learning and pattern recognition method used for classification and regression tasks, for spectral and complexity analysis of cardiovascular and cerebrovascular controls in patients undergoing surgery for aortic valve replacement [81]. In a study by Shaw et al., FASSTER time-varying DLM, which utilizes multi-DLM switching to enhance the precision of modeling the impact of external factors on a time series, was employed for ICP forecasting ([85]; median absolute error = 2.98 mmHg). Lastly, Sourina et al. used fractal analysis, which produces a numerical metric that characterizes the self-replicating patterns identified in time-series data, with box-counting and Higuchi algorithms, for the prediction of changes in health status of a TBI patient [86].

#### 3.2.4. Model Comparison Studies

Various time-series analysis techniques and machine learning algorithms were compared mainly for prediction tasks using ICP data of TBI patients [2,3,61,63,76,79,83,87,88,91,93,94,97,98], while a small portion of the studies performed comparisons of models for the modeling of CBFv signals from patients with various conditions to assess CA [66,67,68,75,84]. It was reported that the employed models possessed the capacity to either predict ICP or model CBFv signals to some extent. However, machine learning models, specifically ensemble learning models, such as random forest and ExtraTrees, showed better performance overall in terms of ICP prediction, whereas in a few cases [63,76], time/frequency domain studies had better prediction performances compared to simple machine learning techniques.

One study assessed the Gaussian process (GP) algorithm, which is a probabilistic machine learning technique used for regression and classification, and logistic regression for the prediction of increased ICP episodes [63]. Another employed IMPFA, which is a model that embraces nonlinear dynamics theories and does not make presumptions about linearity or stationarity; MMPF, which is a method used for studying the pressure–flow relationship proposed to handle nonstationary signals better; and TFA models to examine CBFv [66]. A study by Jachan et al. utilized the ARMAX model, VAR model, and nonparametric transfer function estimator, which is a method of modeling and analyzing input–output relationships of dynamic systems without imposing structural assumptions, to assess dynamic CA ([67], *p*-value = 0.45). Kostoglou et al. modeled CBFv by comparing the performances of ARX and the impulse response model based on LET, which is a mathematical tool that transforms complex functions into a series of simpler Laguerre functions ([68], *p*-value < 0.035). In another study, Miller et al. compared TFA and GHW, which is a mathematical model that analyzes data in the time–frequency domain, and the wavelet transform to quantify dynamic CA ([75], *p*-value < 0.003). Myers et al. utilized GP, logistic regression, and the AR-OR model, which combines elements of the AR model with ordinal regression to analyze ordinal data with temporal dependencies, for the prediction of intracranial hypoxia and tissue hypoxia crises in severe TBI patients [76].

Various ensemble machine learning models such as XGBoost, LGBM, AdaBoost, ExtraTrees, and random forest were used for comparison in several studies. XGBoost and LGBM build an ensemble of decision trees through gradient boosting used for regression and classification tasks [79], AdaBoost combines the predictions of weak classifiers to create a strong classifier, and ExtraTrees leverages the power of decision trees and randomization to create efficient and robust predictive models [3]. Additionally, random forest combines multiple decision trees to improve the accuracy of the model for classification and regression tasks [61]. Farhadi et al. compared the performance of ARIMA and the ETS model, which is a time-series forecasting method that uses exponentially decreasing weights to assign higher weight to the most recent observations; linear regression; Lasso regression, which differs from linear regression by the addition of a feature selection mechanism and regularization to prevent overfitting; SVM; and random forest in forecasting ICP episodes ([61]; NMSE = 0.89 with random forest). Petrov et al. utilized random forest, XGBoost, and LGBM for onset ICP crisis prediction ([79]; precision = 0.76 and accuracy = 0.86 with random forest), achieving the highest reported accuracy among the studies included. In another study, Scalzo et al. employed multiple linear regression, AdaBoost, and ExtraTrees for the temporal prediction of intracranial hypertension ([3]; AUC = 0.87~0.96 with ExtraTrees).

Five studies compared performances of ANN [87,88,97,98] and RNN [91] with non-machine learning techniques for the prediction of ICP. One study compared the performances of ANN, ARX, and Kalman filtering, which is a recursive mathematical algorithm used for estimating the state of a dynamic system [87]. In a consecutive study by Swiercz et al., ANN with wavelet decomposition was compared to AR with Kalman filtering [88]. Tsui et al. compared RNN with MDP, which employs the discrete wavelet transform to calculate wavelet coefficients [91]. Zhang et al. compared an NARX-ANN-based mean forecast algorithm (ANN_NARX_-MFA), nonlinear autoregressive ANN algorithm (ANN_NAR_), and ARMA [97]. In a consecutive study, Zhang et al. utilized ARIMA based on the partial autocorrelation function (PACF) and autocorrelation function (ACF), ARIMA based on the Akaike information criterion (AIC), and ANN models [98].

Schäck et al. proposed a new method, robust time-varying generalized partial directed coherence with the Kalman filter, for nonlinear causality analysis of multivariate time series of physiological data and compared the performances with DEKF, which is a recursive estimation algorithm used for state estimation in dynamic systems [83]. Semenyutin et al. employed CWT and STFT, which is a model used for analyzing non-stationary signals in the time–frequency domain, to determine the state of CA [84]. Wijayatunga et al. employed the probabilistic Markov model, which can analyze the time-dependent behavior of a system, and six different AR models for the prediction of individual ICU patients’ future ICP levels [93]. In two separate studies, Zeiler et al. utilized LME, which is a statistical tool used for analyzing data with dependencies, repeated measurements, and hierarchical structures [118], with the ARIMA model for the estimation of PRx for TBI patients in a large dataset [2] and for a small dataset [94].

### 3.3. Animal Studies

The studies with animal cohorts utilized various animals, including albino mature outbred male rats [99], Wistar male rats [101], female hooded lister rats [102], and, in one instance, goats [100]. The studies measured either CBF with a laser Doppler flowmetry [99,102] or an electromagnetic flow probe [100], or CBFv using a TCD probe [101]. All studies carried out modeling tasks and reported an effective modeling of CBFv or CBF signals to assess CA. The animal cohort studies are listed in Table 4 and categorized based on cerebral physiologic signals and the modeling technique, along with a comment on their modeling ability.

Wavelet analysis was employed by Alexandrin to study the myogenic response of pial arteries [99]. Doblar et al. employed Fourier analysis to examine the effects of hypoxia on the dynamic characteristics of cerebrovascular responses [100]. Issam et al. utilized cross-spectral analysis to investigate the regulation of CBF in response to emotional stress [101]. In a study by Zheng and Mayhew, a comparative analysis between Mandeville’s VM and EW models, which are mathematical models used in cardiovascular physiology to describe and simulate the behavior of the circulatory system, for modeling CBF and cerebral blood volume (CBV) was carried out [102].

## 4. Discussion

In this systematic scoping review, we comprehensively analyzed the current landscape of cerebral physiological time-series modeling and forecasting. Our examination of the selected studies reveals a multifaceted field characterized by diverse methodologies and approaches ranging from statistical models to machine learning algorithms. In this section, we aim to elaborate on the current knowledge, identify knowledge gaps, present the limitations of the literature and our review, and chart a course for future research endeavors. Overall, we observed that the statistical time-series analysis techniques have been the most utilized methods for cerebral physiology modeling with an increased utilization of machine learning algorithms especially for the prediction task. Among the machine learning algorithms, SVM has been the most employed, whereas Decision Tree has been shown to outperform even the SVM model. It was also observed that the nonlinear and multiple-input models, in general, had better performance. The following paragraphs further detail these findings.

In our comprehensive review of 88 studies, we observed that the majority of these studies employed statistical time-series analysis techniques, totaling 65 in number. Among these, several techniques were prominently utilized. Notably, TFA was the focus of investigation in six studies conducted on healthy cohorts [19,26,32,43,47,54] and four studies on patient cohorts [56,65,78,82]. Wavelet analysis emerged as another prominent technique, featuring nine studies conducted on healthy cohorts [1,21,22,23,28,36,50,52,53], four studies on patient cohorts [64,69,90,92], and one study conducted on an animal cohort [99]. Furthermore, variations in AR models for time-series modeling were explored in six studies involving healthy cohorts [27,30,31,37,38,46] and five studies focusing on patient cohorts [59,80,89,95,96]. Another 14 studies of time-series analysis techniques employed a range of frequency domain analysis methods, including cross-spectral analysis [20,35,101], DBI [70,71], Fourier analysis [62,100], Granger causality [73,89,95,96], the multiple coherence function [48,50], power spectrum analysis [51], Welch’s method [33], and Windkessel models [102], as well as various dynamic domain analysis methods, including the moving correlation coefficient [58] and single pulse analysis [60]. A further 10 studies employed time-series analysis techniques within the framework of comparative studies. These comparative analyses encompassed a range of intriguing investigations, including examinations between the Laguerre–Wiener method, FFT, and Tiecks model [44]; examinations of ZAMD, TFA, and ARMA [49] in healthy populations; evaluations contrasting IMPFA, MMPF, and TFA [66]; and assessments of ARMAX, VAR, and the nonparametric transfer function estimator [67]. There were also comparisons between ARX with the impulse response model based on LET [68], in-depth examinations contrasting TFA, GHW, and wavelet transform [75], and investigations into the distinctions between the robust time-varying generalized partial directed coherence with the Kalman filter and DEKF [83]. Additionally, there were comparisons between CWT and STFT [84], and comparisons of Sx_a, Mx_a, and Dx_a LME models in conjunction with ARIMA [2,94] in patient populations. The remaining six studies utilizing time-series analysis techniques conducted comparative analyses of input quantities, including single-input and multiple-input scenarios, as well as assessments of linearity. These studies encompassed various comparisons, such as the evaluation of cross-spectral analysis (single-input) versus the ARMAX model (two-input) [29]; comparisons between one-input and two-input, discrete-time Laguerre function models [34]; nonlinear and linear, single-input and two-input PDM-based models; nonlinear, two-input, second-order LVN models and nonlinear one-input TFA [39]; examinations of three-input and two-input, linear and nonlinear PDMs [40]; as well as LVN models (one-input and two-input, encompassing linear and nonlinear models of first, second, and third order) [41,42].

Among the 88 studies in our review, a subset of 23 studies engaged in the utilization of machine learning algorithms. In four studies involving healthy populations, machine learning algorithms were applied in various comparison studies. These comparisons included assessments of AR SVM versus FIR SVM models [24], as well as evaluations of TFA, NAR SVM, and NFIR SVM models [13]. Furthermore, investigations involved the comparison of FIR SVM, NFIR SVM, NARX SVM, and ARX SVM models [25], along with a comparison encompassing TLRN, the Aaslid–Tieck model, the Laguerre–Wiener method, TFA, and simple linear regression [45]. For patient population studies, the focus shifted to both individual machine learning algorithm analyses with eight studies and comparative analyses with eleven studies. The former involved the application of HMM [55], CCF- and CD-SVM [57], ANN [72], the TSAM algorithm [74], wavelet-based k-means clustering [77], k-NN [81], FASSTER time-varying DLM [85], and fractal analysis utilizing box-counting and Higuchi algorithms [86]. The latter comprised comparisons between ARIMA, ETS models, linear regression, Lasso regression, SVM, and random forest [61]; comparisons between the GP algorithm and logistic regression [63], evaluations comparing the GP algorithm, logistic regression, and AR-OR models [76]; and comparisons between random forest, XGBoost, and LGBM [79]. Additional comparisons encompassed multiple linear regression, AdaBoost, and ExtraTrees [3], as well as assessments involving ANN, ARX, and Kalman filtering [87]. Further, investigations focused on comparisons between ANN with wavelet decomposition and AR with Kalman filtering [88], comparisons between MDP and RNN [91], and comparisons between the probabilistic Markov model and six different AR models [93]. Lastly, the analysis extended to comparisons between ANN_NARX_-MFA, ANN_NAR_, and ARMA [97], as well as comparisons between ARIMA based on PACF and ACF, and ARIMA based on AIC and ANN [98].

It is worth noting that within our systematic scoping review of 88 papers, a distinct division was observed in the methodologies applied. While a substantial portion of the studies, comprising 65 out of the 88, opted for traditional statistical time-series analysis techniques, a smaller subset of 23 papers utilized machine learning algorithms. The prevalence of statistical time-series analysis techniques, particularly variations in AR models, TFA, and wavelet analysis, highlights their historical significance, reliability, and ease of interpretation. Researchers have traditionally relied on these well-established methods to extract meaningful insights from temporal data. Subsequently, in the analysis of the healthy and patient population data in this review, statistical time-series analysis and modeling techniques demonstrated their efficacy in capturing cerebral physiologic signal relationships. However, the use of machine learning algorithms in this domain signals a growing recognition of their ability to uncover intricate patterns, particularly in cases where data complexities call for more adaptive and data-driven modeling approaches. Through comparative studies pitting machine learning algorithms against statistical analysis techniques for modeling as well as prediction abilities, a distinct advantage was observed in favor of machine learning algorithms [13,25,45,87,88,93,97] with the exception of one study [98], where ARIMA based on PACF and ACF with a higher accuracy with a mean R^2^ of 0.898 outperformed the ANN model with a mean R^2^ of 0.804. This observation with the exception of the aforementioned study underscores the superior predictive power and adaptability of machine learning methods in this context. Furthermore, the comparative analyses consistently favored the use of nonlinear models [24,39,41,42]. This trend was also prevalent with studies that perform modeling in conjunction with multiple-input signals [29,34,40,41,42]. The prevalence of multiple-input models exhibiting superior performance underscores the significance of multivariate modeling and forecasting in this domain. It reinforces the notion that incorporating information from multiple sources or dimensions enhances our ability to understand and predict complex dynamic systems effectively.

Notably, the majority of studies leaned towards nonlinear models, highlighting their ability to capture the inherent complexities within the data. It is worth noting that this preference was consistent across various machine learning algorithms and statistical time-series analysis techniques. However, in the study by Panerai et al., the linear Laguerre–Wiener method notably outperformed its nonlinear counterpart during thigh cuff tests. This observation raises an intriguing point regarding the influence of the temporal pattern of MABP fluctuations on the performance of nonlinear models [44]. This exceptional result suggests that the specific characteristics of the data, particularly the temporal patterns of MABP, may serve as crucial factors in determining the choice between linear and nonlinear modeling approaches. Nonetheless, it is essential to exercise caution when generalizing from this single instance. To validate this notion, further studies involving comparisons between linear and nonlinear models should be conducted, specifically with data collection under thigh cuff test conditions. These investigations are necessary to confirm the potential impact of temporal patterns on model performance. Additionally, the incorporation of machine learning methods warrants further scrutiny and validation to comprehensively assess their efficacy in this context.

In our scoping review, we observed variations in the prediction accuracy of different studies that focused on forecasting cerebral physiologic signals. Only a limited number of studies conducted prediction tasks within a healthy population. Liu and Allen demonstrated the success of the ARX model in predicting step responses under various conditions [38]. Marmarelis et al. showed significantly reduced prediction errors using nonlinear two-input PDM models in CBFV prediction [39], while in a consecutive study, Marmarelis et al. highlighted the importance of including HR and nonlinearities in reducing prediction errors [40]. In two separate studies, Mitsis et al. emphasized the benefits of incorporating EtCO_2_ as an input and leveraging nonlinear models to achieve the lowest output prediction errors [41,42]. In contrast, a larger number of studies have concentrated on prediction within patient populations. Asgari et al. explored various regression and forecasting models, with Lasso regression and random forest demonstrating high accuracy for ICP forecasting in a patient population [55]. Similarly, Güiza et al. favored the GP model for predicting ICP episodes in TBI patients [55]. Myers et al. identified the crucial role of ICP and its changes in predicting elevated ICP and hypoxic events, with the AR-OR model providing advance warnings [76]. Petrov et al. highlighted the superior performance of random forest in onset ICP crisis prediction [79], while Scalzo et al. found that ExtraTrees was effective in temporal ICP prediction, the performance of which was followed by AdaBoost and multilinear classifiers [3]. In two separate studies, Swiercz et al. showed that ANN outperformed traditional predictors, especially when combined with wavelet decomposition [87,88]. Tsui et al. introduced the MDP model as an efficient ICP predictor in short- and long-term intervals [91]. Wijayatunga et al. developed probabilistic Markov and AR models for individual patient ICP predictions [93]. Zeiler et al. demonstrated, in two separate studies, the effectiveness of LME models with ARIMA for PRx estimation [2,94]. Similarly, two separate studies by Zhang et al. favored ARIMA models based on PACF and ACF for continuous trend prediction [97,98]. These studies collectively provide a comprehensive overview of the diverse predictive capabilities, especially in the field of ICP prediction, offering valuable insights for patient care and management.

In the context of cerebral physiology signals, the predominant focus in the majority of the studies has been on the modeling and prediction of CBFv and ICP signals. In the healthy population studies, the primary focus of analysis and modeling centered around CBFv. In contrast, within the patient population, particularly among TBI patients with varying severity, most studies predominantly concentrated on modeling ICP. This shift in focus towards ICP is crucial from a clinical perspective, as it aligns with the critical importance of ICP prediction in clinical practice. However, this concentration has resulted in a noticeable gap in the exploration of the signal modeling and prediction of HbO with twelve articles [19,21,22,23,28,36,43,52,53,64,70,71], rSO_2_ with one article [1], and SctO_2_ with one article [90], leaving significant room for further investigation and research in these areas. On the other hand, while the number of animal studies in our review was limited, it is worth noting that all of these studies were dedicated to modeling CBF. In these animal studies, a consistent pattern emerged as they exclusively relied on statistical analysis methods to achieve their modeling objectives.

As previously mentioned, machine learning algorithms consistently demonstrated superior performance when compared to statistical time-series analysis techniques. Specifically, SVM stands out as an extensively employed method [13,24,25,57,61], consistently exhibiting excellent predictive capabilities. Nevertheless, notable findings from various studies indicate that ExtraTrees [3] and random forest [61,79], which are ensemble learning algorithms, have consistently outperformed other machine learning models such as SVM, linear regression, Lasso regression, and XGBoost. These findings underscore the potential promise of ensemble learning methods, and they merit further investigation and validation in comparison with both statistical time-series analysis techniques and additional machine learning algorithms, including ANN, HMM, and deep learning approaches such as RNN.

Future research endeavors should focus on comprehensive performance assessments to elucidate the strengths and limitations of these modeling approaches within the context of cerebral physiologic signal analysis. Furthermore, exploring the potential of deep learning algorithms, such as CNN and long short-term memory (LSTM), which is a type of RNN known for its ability to capture long-range dependencies, holds significant promise. LSTM possesses unique capabilities that enable it to effectively learn and leverage temporal dependencies, which can potentially result in substantial improvements in prediction accuracy. On the other hand, CNN excels in capturing spatial and hierarchical patterns in data. By combining the temporal modeling strengths of LSTM with the pattern recognition capabilities of CNN, it could be possible to enhance cerebral physiology modeling and prediction.

### 4.1. Limitations of the Literature

We have identified 88 studies with varying study cohorts and cerebral physiologic data. Among the healthy cohort, the CBFv recorded with TCD probes from the MCA was the most studied cerebral physiologic signal. In patient cohorts, although the majority of the studies included ICP signals, several studies assessed CBFv signals recorded with a TCD probe from the MCA. These studies assumed that the changes in CBFv were indicative of alterations in CBF which relies on the assumption that the diameter of the insonated segment of the mid-cerebral artery remains constant. However, this assumption may not hold true in all situations as the diameter of blood vessels could change as a response to variations in blood flow [119]. Additionally, TCD ultrasound may encounter various technical limitations, including technician proficiency, the clarity of the temporal bone window, and the impact of insonation angles on recorded CBFv measurements [120]. If the assumption that the diameter of the insonated segment remains constant is not correct, it can lead to inaccuracies in estimating actual CBF based on CBFv measurements. Another limitation arises from the use of P_RA_ and CrCP, which are estimated using ABP signals; hence, the accurate estimation of P_RA_ relies on the accuracy of non-invasive measurements of ABP [47]. In the studies modeling ABP and CBFv, the variance in measurement positions may introduce a time lag between CBFv and ABP recordings, potentially leading to an overestimation of the phase shift at HF ranges [49]. Furthermore, this systematic review highlights the scarcity of studies and underscores the necessity for further research to analyze the potential relationship between cerebral pressure–flow and specific neurological conditions, such as brain tumors, Alzheimer’s disease, and Parkinson’s disease. Finally, it is important to note that studies focusing on prediction lack a standardized approach for reporting prediction accuracy or error metrics. This absence of a consistent reporting style hinders the comparability and cross-validation of results with other studies, potentially impeding the synthesis of findings within the field.

### 4.2. Limitations of This Review

This systematically conducted review aimed to provide a comprehensive scoping overview of the literature on time-series analysis, modeling, and prediction within the realm of cerebral physiologic signals. Nevertheless, several inherent limitations should be acknowledged. Firstly, the inherent heterogeneity in experimental designs, subjects, and data modalities across the included studies precluded the possibility of conducting a meta-analysis. The diversity of experimental conditions rendered a meta-analysis neither feasible nor appropriate, as the studies exhibited substantial variability. Secondly, we excluded EEG studies from this systematic scoping review to specifically focus on cerebral pressure–flow dynamics. While EEG represents a significant component of cerebral physiology research, its extensive representation in the literature of cerebral electrophysiologic signals modeled warrants a dedicated review of its own. Thirdly, our focus was exclusively on time-series signals, and as such, we did not include imaging modalities, such as CT, PET, and MRI, within the scope of this review. Imaging studies, although valuable in cerebral physiology research, do not typically capture temporally resolved data and, therefore, were not considered in this analysis. Fourth, while efforts were made to include the most up-to-date research, it should be noted that some of the latest developments may not have been covered in this review, due to the database search dateline, which ended in mid-March 2023. Lastly, it is important to note that this review only includes English-language studies, which may introduce a potential language bias and potentially limit the comprehensiveness of our review. These limitations underscore the need for future research endeavors to address the challenges posed by heterogeneity, explore the rich landscape of EEG studies, and delve into the distinctive characteristics of imaging modalities in the context of cerebral physiologic signal analysis. Additionally, efforts to encompass a wider linguistic range of studies can further enrich our understanding of this complex domain.

### 4.3. Future Directions

In our systematic review of cerebral physiologic signal time-series analysis, we observed notable research gaps. While machine learning algorithms, particularly SVM and ensemble learning methods like ExtraTrees and random forest, have consistently demonstrated superior performance compared to statistical time-series analysis techniques in terms of prediction, there remains a need for comprehensive assessments, including comparisons with additional machine learning models such as ANN, HMM, and deep learning approaches like RNN. Additionally, the potential of deep learning algorithms, such as CNN and LSTM, has been underexplored, despite their ability to capture long-range dependencies and spatial patterns, respectively, which could significantly enhance cerebral physiology modeling and prediction. Moreover, our systematic review highlights the prevalence of studies leaning towards nonlinear models. To delve deeper into this trend and fully comprehend its implications, further research should explore the applicability of nonlinear models across various cerebral physiologic signals and experimental conditions. Investigating the robustness of these models under diverse scenarios will contribute to a more nuanced understanding of their performance. Furthermore, our review revealed a concentration on modeling and predicting CBFv and ICP signals, leaving a considerable research gap in the exploration of HbO and rSO_2_ signal modeling and prediction. Additionally, there is potential for integrating assessment through the utilization of smart chip-based sensors to process data directly within the device or through a separate device that consolidates multiple signals for predictive analysis. Such advancements hold promise for enhancing diagnostic capabilities and facilitating real-time monitoring, thus warranting further investigation. Future research endeavors should aim to fill these gaps by conducting comprehensive assessments of ensemble learning methods, exploring the potential of deep learning algorithms, and dedicating efforts to the modeling and prediction of oxygenation signals, ultimately advancing our understanding and applications in cerebral physiologic signal analysis.

## 5. Conclusions

In conclusion, our systematic scoping review of cerebral physiologic signal time-series analysis aimed to provide a comprehensive understanding of existing methodologies for the modeling/prediction of continuous cerebral pressure–flow dynamics and to identify areas for future research. We have observed that machine learning algorithms, notably SVM and ensemble learning methods like ExtraTrees and random forest, exhibit promising capabilities in modeling and prediction, highlighting the need for comprehensive assessments against a wider array of machine learning models. Furthermore, the untapped potential of deep learning algorithms, including CNN and LSTM, offers exciting avenues for improving accuracy in cerebral physiology modeling. We also emphasize the importance of addressing the research gap in the modeling and prediction of HbO and rSO_2_ signals, as this remains a relatively unexplored area with significant clinical implications. By addressing the identified gaps, substantial contributions could be made for the advancement of cerebral physiologic signal analysis, ultimately improving real-time interpretation and prediction for the benefit of life, health, and veterinary sciences.

## Figures and Tables

**Table 1:** Healthy human populations—summary of cerebral physiologic modeling and prediction abilities.

Time–Frequency Domain Modeling Techniques			
Cerebral Physiologic Metric	Number of Studies and Technique	Temporal Modeling Ability	Prediction/Forecasting Ability
CPP	Cross-spectral analysis—1 study [29]	Successfully modeled.	Not explored.
CBFv	Aaslid–Tiecks model—1 study [44]	Effective modeling of CBFv signal was reported ([51], *p*-value < 0.01; [32], *p*-value < 0.05; [50], *p*-value < 0.05; [19], *p*-value < 0.05; [43], *p*-value < 0.05).	Not explored.
	Cross-spectral analysis—3 studies [20,30,35,45]		
	Discrete-time Laguerre function model—1 study [34]		
	FFT—1 study [44]		
	Multiple coherence analysis—2 studies [33,48]		
	Laguerre–Wiener method—2 studies [44,45]		
	LVN model—2 studies [41,42]		
	PDM-based model—2 studies [39,40]		
	Power spectrum analysis—1 study [51]		
	TFA—9 studies [19,26,32,39,40,43,49,51,103]		
	Wavelet analysis—1 study [50]		
	Welch method—1 study [33]		
	ZAMD—1 study [49]		
CA	Cross-spectral analysis—1 study [20]	Successfully modeled.	Not explored.
	Discrete-time Laguerre function model—1 study [34]		
	PDM-based model—2 studies [39,40]		
	TFA—5 studies [39,40,47,49,103]		
	Welch method—1 study [33]		
	ZAMD—1 study [49]		
NIRS *	TFA—1 study [19]	Effective modeling of Δ[HbO] signal was reported ([21], *p*-value < 0.04; [22,23], *p*-value < 0.04 in both studies; [28], *p*-value < 0.03; [36], *p*-value < 0.014; [52], *p*-value < 0.03; [53], *p*-value < 0.05; [50], *p*-value < 0.05).	Not explored.
	Wavelet analysis—8 studies [1,21,22,23,28,36,52,53]		
**Autoregressive Modeling Techniques**			
**Cerebral Physiologic Metric**	**Number of Studies and Technique**	**Temporal Modeling Ability**	**Prediction/Forecasting Ability**
CPP	ARMAX—1 study [29]	Successfully modeled.	Not explored.
CBFv	ARMA—3 studies [27,46,49]	Effective modeling of CBFv signal was reported ([27], *p*-value = 0.003; [46], *p*-value < 0.03; [31], *p*-value < 0.3; [37], *p*-value < 0.001).	Not explored.
	ARMAX—1 study [29]		
	ARX—3 studies [31,37,38]		
CA	ARMA—3 studies [27,30,49]	Successfully modeled.	Not explored.
	ARX—2 studies [31,38]		
**Machine Learning Techniques**			
**Cerebral Physiologic Metric**	**Number of Studies and Technique**	**Temporal Modeling Ability**	**Prediction/Forecasting Ability**
CBFv	Linear regression—1 study [45]	Effective modeling of CBFv signal was reported ([24], *p*-value < 0.002; [13], *p*-value < 0.001)	Not explored.
	SVM—3 studies [13,24,25]		
	TLRN—1 study [45]		
CA	SVM—3 studies [13,24,25]	Successfully modeled.	Not explored.

ARMA = autoregressive moving average, ARMAX = autoregressive moving average with exogenous input, ARX = autoregressive with exogenous input, CA = cerebral autoregulation, CBF = cerebral blood flow, CBFv = cerebral blood flow velocity, CPP = cerebral perfusion pressure, FFT = fast Fourier transform, LVN = Laguerre–Volterra network, NIRS = near-infrared spectroscopy, PDM = principal dynamic mode, TFA = transfer function analysis, TLRN = time-lagged recurrent neural network, SVM = support vector machine, ZAMD = Zhao–Atlas–Marks distribution. * NIRS represents oxy- and deoxyhemoglobin signals (Δ[HbO], etc.), as well as process saturation measures such as regional oxygen saturation (rSO_2_).

**Table 2:** Human TBI patient populations—summary of cerebral physiologic modeling and prediction abilities.

Time–Frequency Domain Modeling Techniques			
Cerebral Physiologic Metric	Number of Studies and Technique	Temporal Modeling Ability	Prediction/Forecasting Ability
ICP	DEKF—1 study [83]	Effective modeling of ICP signal was reported ([92], *p*-value < 0.1).	Not explored.
	GP algorithm—2 studies [63,76]		
	Granger causality—3 studies [89,95,96]		
	MDP—1 study [91]		
	Moving correlation coefficient—1 study [58]		
	Probabilistic Markov model—1 model [93]		
	Robust time-varying generalized partial directed coherence with Kalman filter—1 study [83]		
	TFA—1 study [65]		
	Wavelet analysis—1 study [69]		
CPP	GP algorithm—1 study [63]	Successfully modeled.	Not explored.
	Moving correlation coefficient—1 study [58]		
CBFv	TFA—2 studies [65,92]	Effective modeling of CBFv signal was reported ([92], *p*-value < 0.1).	Not explored.
CA	Wavelet analysis—1 study [69]	Successfully modeled.	Not explored.
PbtO_2_	DEKF—1 study [83]	Successfully modeled.	Not explored.
	GP algorithm—1 study [76]		
	Granger causality—1 study [96]		
	Robust time-varying generalized partial directed coherence with Kalman filter—1 study [83]		
**Autoregressive Modeling Techniques**			
**Cerebral Physiologic Metric**	**Number of Studies and Technique**	**Temporal Modeling Ability**	**Prediction/Forecasting Ability**
ICP	AR—2 studies [88,93]	Effective modeling of ICP signal was reported ([95], *p*-value < 0.3).	Not explored.
	AR-OR—1 study [76]		
	ARIMA—5 studies [2,89,94,96,98]		
	ARMA—2 studies [59,97]		
	VARFI—1 study [80]		
	VARIMA—3 studies [89,95,96]		
CPP	ARMA—1 study [59]	Successfully modeled.	Not explored.
	VARFI—1 study [80]		
	VARIMA—1 study [95]		
CBFv	ARIMA—2 studies [2,94]	Successfully modeled.	Not explored.
PbtO_2_	AR-OR—1 study [76]	Successfully modeled.	Not explored.
	ARIMA—1 study [96]		
	VARIMA—1 study [96]		
**Machine Learning Techniques**			
**Cerebral Physiologic Metric**	**Number of Studies and Technique**	**Temporal Modeling Ability**	**Prediction/Forecasting Ability**
ICP	ANN—3 studies [88,97,98]	Not explored.	Adequate performance to predict ICP was reported ([79]; precision = 0.76 and accuracy = 0.86 with random forest).
	HMM—1 study [55]		
	FASSTER time varying DLM—1 study [85]		
	Fractal analysis with box-counting and Higuchi algorithms—1 study [86]		
	LGBM—1 study [79]		
	Logistic regression—2 studies [63,76]		
	Random forest—1 study [79]		
	RNN—1 study [91]		
	Wavelet-based k-means clustering—1 study [77]		
	XGBoost—1 study [79]		
CPP	HMM—1 study [55]	Not explored.	Adequate prediction performance was reported.
	Logistic regression—1 study [63]		
PbtO_2_	Logistic regression—1 study [76]	Not explored.	Adequate prediction performance was reported.

ANN = artificial neural network, AR = autoregressive, AR-OR = autoregressive ordinal-regression, ARIMA = autoregressive integrated moving average, ARMA = autoregressive moving average, CA = cerebral autoregulation, CBF = cerebral blood flow, CBFv = cerebral blood flow velocity, CPP = cerebral perfusion pressure, DEFK = dual extended Kalman filter, DLM = dynamic linear model, FASSTER = forecasting with additive switching of seasonality, trend and exogenous regressors, GP = Gaussian process, HMM = hidden Markov model, ICP = intracranial pressure, LGBM = light gradient boosting model, MDP = multiresolution dynamic predictor, PbtO_2_ = partial pressure of brain tissue oxygen, RNN = recurrent neural network, TFA = transfer function analysis, VARFI = vector autoregressive fractionally integrated, VARIMA = vector autoregressive integrated moving average, XGBoost = extreme gradient boosting.

**Table 3:** Human non-TBI patient populations—summary of cerebral physiologic modeling and prediction abilities.

Time–Frequency Domain Modeling Techniques			
Cerebral Physiologic Metric	Number of Studies and Technique	Temporal Modeling Ability	Prediction/Forecasting Ability
ICP	ETS model—1 study [61]	Not explored.	Adequate performance to predict ICP was reported.
	Granger causality with EEMD—1 study [73]		
	Kalman filtering—1 study [87]		
	Single pulse analysis—1 study [60]		
CBFv	CWT—1 study [84]	Effective modeling of CBFv signal was reported ([56], *p*-value = 0.052; [78], *p*-value < 0.0009; [82], *p*-value < 0.02; [69], *p*-value < 0.05; [90], *p*-value < 0.3; [92], *p*-value < 0.1).	Not explored.
	FFT—1 study [62]		
	GHW—1 study [75]		
	Granger causality with EEMD—1 study [73]		
	IMPFA—1 study [66]		
	Impulse-response-based LET model—1 study [68]		
	MMPF—1 study [66]		
	Nonparametric transfer function estimator—1 study [67]		
	STFT—1 study [84]		
	TFA—4 studies [6,66,75,82]		
	Wavelet analysis—1 study [69]		
CA	GHW—1 study [75]	Successfully modeled.	Not explored.
	TFA—4 studies [6,56,75,82]		
	Wavelet analysis—2 studies [75,90]		
NIRS *	DBI—2 studies [70,71]	Effective modeling of Δ[HbO] signal was reported ([70], *p*-value < 0.02; [71], *p*-value < 0.03; [64], *p*-value < 0.4).	Not explored.
	Wavelet analysis—2 studies [64,90]		
**Autoregressive Modeling Techniques**			
**Cerebral Physiologic Metric**	**Number of Studies and Technique**	**Temporal Modeling Ability**	**Prediction/Forecasting Ability**
ICP	ARIMA—1 study [61]	Successfully modeled.	Not explored.
	ARX—1 study [87]		
CBFv	ARMAX—1 study [67]	Successfully modeled.	Not explored.
	ARX—1 study [68]		
	VAR—1 study [67]		
CA	ARX—1 study [68]	Successfully modeled.	Not explored.
**Machine Learning Techniques**			
**Cerebral Physiologic Metric**	**Number of Studies and Technique**	**Temporal Modeling Ability**	**Prediction/Forecasting Ability**
ICP	AdaBoost—1 study [3]	Not explored.	Adequate performance to predict ICP was reported ([61]; NMSE = 0.89 with random forest, [3]; AUC = 0.87~0.96 with ExtraTrees).
	ANN—2 studies [72,87]		
	ExtraTrees—1 study [3]		
	Lasso regression—1 study [61]		
	Linear regression—2 studies [3,61]		
	Random forest—1 study [61]		
	SVM—1 study [61]		
	TSAM algorithm—1 study [74]		
CPP	TSAM algorithm—1 study [74]	Not explored.	Successfully modeled.
CBFv	k-NN—1 study	Successfully modeled.	Not explored.
	SVM—1 study [57]		
CA	SVM—1 study [57]	Successfully modeled.	Not explored.
PbtO_2_	TSAM algorithm—1 study [74]	Successfully modeled.	Not explored.

AdaBoost = adaptive boosting, ANN = artificial neural network, ARIMA = autoregressive integrated moving average, ARMAX = autoregressive moving average with exogenous input, ARX = autoregressive with exogenous input, CA = cerebral autoregulation, CBFv = cerebral blood flow velocity, CPP = cerebral perfusion pressure CWT = cross-wavelet transform, DBI = dynamical Bayesian inference, EEMD = ensemble empirical mode decomposition, ETS = exponential smoothing, ExtraTrees = extremely randomized decision trees, GHW = generalized harmonic wavelets, ICP = intracranial pressure, IMPFA = intrinsic multiscale pressure–flow analysis, k-NN = k-nearest neighbor, LET = Laguerre expansion technique, MMPF = multimodal pressure–flow analysis, NIRS = near-infrared spectroscopy, PbtO_2_ = partial pressure of brain tissue oxygen, STFT = short-time Fourier transform, SVM = support vector machine, TFA = transfer function analysis, TSAM = time-varying temporal signal angle measurement, VAR = vector autoregressive. * NIRS represents oxy- and deoxyhemoglobin signals, as well as process saturation measures such as regional oxygen saturation (rSO_2_).

**Table 4:** Animal cohorts—summary of cerebral physiologic modeling and prediction abilities.

Time–Frequency Domain Modeling Techniques		
Cerebral Physiologic Metric	Number of Studies and Technique	Temporal Modeling Ability
CBF	Windkessel models—1 study [102]	Successfully modeled.
	Fourier analysis—1 study [100]	
	Wavelet analysis—1 study [99]	
CBFv	Cross-spectral analysis—1 study [101]	Successfully modeled.

CBF = cerebral blood flow, CBFv = cerebral blood flow velocity.

## Data Availability

This review relied on academic literature databases that are publicly available.

## Supplementary Materials

The following supporting information can be downloaded at https://www.mdpi.com/article/10.3390/s24051453/s1. Supplementary Appendix SA—Search strategy for BIOSIS; Supplementary Appendix SB—Detailed summary of the systematic review.

## References

[1] Addison P.S. Identifying Stable Phase Coupling Associated with Cerebral Autoregulation Using the Synchrosqueezed Cross-Wavelet Transform and Low Oscillation Morlet Wavelets. Proceedings of the 2015 37th Annual International Conference of the IEEE Engineering in Medicine and Biology Society (EMBC).

[2] Zeiler F.A., Smielewski P., Donnelly J., Czosnyka M., Menon D.K., Ercole A. (2018). Estimating Pressure Reactivity Using Noninvasive Doppler-Based Systolic Flow Index. J. Neurotrauma.

[3] Scalzo F., Hamilton R., Asgari S., Kim S., Hu X. (2012). Intracranial Hypertension Prediction Using Extremely Randomized Decision Trees. Med. Eng. Phys..

[4] Komlodi-Pasztor E., Gilbert M.R., Armstrong T.S. (2022). Diagnosis and Management of Stroke in Adults with Primary Brain Tumor. Curr. Oncol. Rep..

[5] Korte N., Nortley R., Attwell D. (2020). Cerebral Blood Flow Decrease as an Early Pathological Mechanism in Alzheimer’s Disease. Acta Neuropathol..

[6] Panerai R.B. (1998). Assessment of Cerebral Pressure Autoregulation in Humans—A Review of Measurement Methods. Physiol. Meas..

[7] Skrifvars M.B., Sekhon M., Åneman E.A. (2021). Monitoring and Modifying Brain Oxygenation in Patients at Risk of Hypoxic Ischaemic Brain Injury after Cardiac Arrest. Crit. Care.

[8] Panerai R.B. (2008). Cerebral Autoregulation: From Models to Clinical Applications. Cardiovasc. Eng..

[9] Silverman A., Petersen N.H. (2023). Physiology, Cerebral Autoregulation. StatPearls.

[10] Ahmadi M., O’Neil M., Fragala-Pinkham M., Lennon N., Trost S. (2018). Machine Learning Algorithms for Activity Recognition in Ambulant Children and Adolescents with Cerebral Palsy. J. NeuroEng. Rehabil..

[11] Hu P., Li Y., Liu Y., Guo G., Gao X., Su Z., Wang L., Deng G., Yang S., Qi Y. (2022). Comparison of Conventional Logistic Regression and Machine Learning Methods for Predicting Delayed Cerebral Ischemia After Aneurysmal Subarachnoid Hemorrhage: A Multicentric Observational Cohort Study. Front. Aging Neurosci..

[12] Islam M.S., Hussain I., Rahman M.M., Park S.J., Hossain M.A. (2022). Explainable Artificial Intelligence Model for Stroke Prediction Using EEG Signal. Sensors.

[13] Chacón M., Jara J.L., Miranda R., Katsogridakis E., Panerai R.B. (2018). Non-Linear Models for the Detection of Impaired Cerebral Blood Flow Autoregulation. PLoS ONE.

[14] Cochrane Handbook Cochrane Handbook for Systematic Reviews of Interventions. https://training.cochrane.org/handbook.

[15] Page M.J., McKenzie J.E., Bossuyt P.M., Boutron I., Hoffmann T.C., Mulrow C.D., Shamseer L., Tetzlaff J.M., Akl E.A., Brennan S.E. (2021). The PRISMA 2020 Statement: An Updated Guideline for Reporting Systematic Reviews. BMJ.

[16] Tricco A.C., Lillie E., Zarin W., O’Brien K.K., Colquhoun H., Levac D., Moher D., Peters M.D.J., Horsley T., Weeks L. (2018). PRISMA Extension for Scoping Reviews (PRISMA-ScR): Checklist and Explanation. Ann. Intern. Med..

[17] Sainbhi A.S., Marquez I., Gomez A., Stein K.Y., Amenta F., Vakitbilir N., Froese L., Zeiler F.A. (2023). Regional Disparity in Continuously Measured Time-Domain Cerebrovascular Reactivity Indices: A Scoping Review of Human Literature. Physiol. Meas..

[18] Siddiqi A.Z., Froese L., Gomez A., Sainbhi A.S., Stein K., Park K., Vakitbilir N., Zeiler F.A. (2023). The Effect of Burst Suppression on Cerebral Blood Flow and Autoregulation: A Scoping Review of the Human and Animal Literature. Front. Physiol..

[19] Ainslie P.N., Barach A., Murrell C., Hamlin M., Hellemans J., Ogoh S. (2007). Alterations in Cerebral Autoregulation and Cerebral Blood Flow Velocity during Acute Hypoxia: Rest and Exercise. Am. J. Physiol. Heart Circ. Physiol..

[20] Brown C.M., Dütsch M., Öhring S., Neundörfer B., Hilz M.J. (2004). Cerebral Autoregulation Is Compromised during Simulated Fluctuations in Gravitational Stress. Eur. J. Appl. Physiol..

[21] Bu L., Li J., Li F., Liu H., Li Z. (2016). Wavelet Coherence Analysis of Cerebral Oxygenation Signals Measured by Near-Infrared Spectroscopy in Sailors: An Exploratory, Experimental Study. BMJ Open.

[22] Bu L., Zhang M., Li J., Li F., Liu H., Li Z. (2017). Effects of Sleep Deprivation on Phase Synchronization as Assessed by Wavelet Phase Coherence Analysis of Prefrontal Tissue Oxyhemoglobin Signals. PLoS ONE.

[23] Bu L., Wang D., Huo C., Xu G., Li Z., Li J. (2018). Effects of Poor Sleep Quality on Brain Functional Connectivity Revealed by Wavelet-Based Coherence Analysis Using NIRS Methods in Elderly Subjects. Neurosci. Lett..

[24] Chacon M., Araya C., Panerai R.B. (2011). Non-Linear Multivariate Modeling of Cerebral Hemodynamics with Autoregressive Support Vector Machines. Med. Eng. Phys..

[25] Chacón M., Rojas-Pescio H., Peñaloza S., Landerretche J. (2022). Machine Learning Models and Statistical Complexity to Analyze the Effects of Posture on Cerebral Hemodynamics. Entropy.

[26] Claassen J.A.H.R., Levine B.D., Zhang R. (2009). Dynamic Cerebral Autoregulation during Repeated Squat-Stand Maneuvers. J. Appl. Physiol..

[27] Clough R.H., Minhas J.S., Haunton V.J., Hanby M.F., Robinson T.G., Panerai R.B. (2022). Dynamics of the Cerebral Autoregulatory Response to Paced Hyperventilation Assessed Using Subcomponent and Time-Varying Analyses. J. Appl. Physiol..

[28] Cui R., Zhang M., Li Z., Xin Q., Lu L., Zhou W., Han Q., Gao Y. (2014). Wavelet Coherence Analysis of Spontaneous Oscillations in Cerebral Tissue Oxyhemoglobin Concentrations and Arterial Blood Pressure in Elderly Subjects. Microvasc. Res..

[29] Edwards M.R., Lin D.C., Hughson R.L., Poon C.-S., Kazemi H. (2001). Modeling the Interaction Between Perfusion Pressure and CO_2_ on Cerebral Blood Flow. Frontiers in Modeling and Control of Breathing: Integration at Molecular, Cellular, and Systems Levels.

[30] Edwards M.R., Devitt D.L., Hughson R.L. (2004). Two-Breath CO_2_ Test Detects Altered Dynamic Cerebrovascular Autoregulation and CO_2_ Responsiveness with Changes in Arterial Pco2. Am. J. Physiol. Regul. Integr. Comp. Physiol..

[31] Gehalot P., Mathew A., Behbehani K., Zhang R. Efficacy of Using Mean Arterial Blood Pressure Sequence for Linear Modeling of Cerebral Autoregulation. Proceedings of the 2005 IEEE Engineering in Medicine and Biology 27th Annual Conference.

[32] Iwasaki K., Ogawa Y., Shibata S., Aoki K. (2007). Acute Exposure to Normobaric Mild Hypoxia Alters Dynamic Relationships between Blood Pressure and Cerebral Blood Flow at Very Low Frequency. J. Cereb. Blood Flow. Metab..

[33] Katsogridakis E., Simpson D.M., Bush G., Fan L., Birch A.A., Allen R., Potter J.F., Panerai R.B. (2016). Revisiting the Frequency Domain: The Multiple and Partial Coherence of Cerebral Blood Flow Velocity in the Assessment of Dynamic Cerebral Autoregulation. Physiol. Meas..

[34] Kostoglou K., Debert C.T., Poulin M.J., Mitsis G.D. (2014). Nonstationary Multivariate Modeling of Cerebral Autoregulation during Hypercapnia. Med. Eng. Phys..

[35] Kuo T.B.-J., Chern C.-M., Sheng W.-Y., Wong W.-J., Hu H.-H. (1998). Frequency Domain Analysis of Cerebral Blood Flow Velocity and Its Correlation with Arterial Blood Pressure. J. Cereb. Blood Flow. Metab..

[36] Li Z., Zhang M., Cui R., Xin Q., Liqian L., Zhou W., Han Q., Gao Y. (2014). Wavelet Coherence Analysis of Prefrontal Oxygenation Signals in Elderly Subjects with Hypertension. Physiol. Meas..

[37] Liu Y., Birch A.A., Allen R. (2003). Dynamic Cerebral Autoregulation Assessment Using an ARX Model: Comparative Study Using Step Response and Phase Shift Analysis. Med. Eng. Phys..

[38] Liu Y., Allen R. (2002). Analysis of Dynamic Cerebral Autoregulation Using an ARX Model Based on Arterial Blood Pressure and Middle Cerebral Artery Velocity Simulation. Med. Biol. Eng. Comput..

[39] Marmarelis V.Z., Shin D.C., Zhang R. (2012). Linear and Nonlinear Modeling of Cerebral Flow Autoregulation Using Principal Dynamic Modes. Open Biomed. Eng. J..

[40] Marmarelis V.Z., Mitsis G.D., Shin D.C., Zhang R. (2016). Multiple-Input Nonlinear Modelling of Cerebral Haemodynamics Using Spontaneous Arterial Blood Pressure, End-Tidal CO_2_ and Heart Rate Measurements. Philos. Trans. R. Soc. A Math. Phys. Eng. Sci..

[41] Mitsis G.D., Poulin M.J., Robbins P.A., Marmarelis V.Z. (2004). Nonlinear Modeling of the Dynamic Effects of Arterial Pressure and CO_2_ Variations on Cerebral Blood Flow in Healthy Humans. IEEE Trans. Biomed. Eng..

[42] Mitsis G.D., Zhang R., Levine B.D., Marmarelis V.Z. (2006). Cerebral Hemodynamics during Orthostatic Stress Assessed by Nonlinear Modeling. J. Appl. Physiol..

[43] Oudegeest-Sander M.H., van Beek A.H.E.A., Abbink K., Olde Rikkert M.G.M., Hopman M.T.E., Claassen J.A.H.R. (2014). Assessment of Dynamic Cerebral Autoregulation and Cerebrovascular CO_2_ Reactivity in Ageing by Measurements of Cerebral Blood Flow and Cortical Oxygenation. Exp. Physiol..

[44] Panerai R.B., Dawson S.L., Potter J.F. (1999). Linear and Nonlinear Analysis of Human Dynamic Cerebral Autoregulation. Am. J. Physiol. Heart Circ. Physiol..

[45] Panerai R.B., Chacon M., Pereira R., Evans D.H. (2004). Neural Network Modelling of Dynamic Cerebral Autoregulation: Assessment and Comparison with Established Methods. Med. Eng. Phys..

[46] Panerai R.B., Salinet A.S.M., Robinson T.G. (2012). Contribution of Arterial Blood Pressure and PaCO_2_ to the Cerebrovascular Responses to Motor Stimulation. Am. J. Physiol. Heart Circ. Physiol..

[47] Panerai R.B., Haunton V.J., Llwyd O., Minhas J.S., Katsogridakis E., Salinet A.S., Maggio P., Robinson T.G. (2021). Cerebral Critical Closing Pressure and Resistance-Area Product: The Influence of Dynamic Cerebral Autoregulation, Age and Sex. J. Cereb. Blood Flow. Metab..

[48] Peng T., Rowley A.B., Ainslie P.N., Poulin M.J., Payne S.J. (2008). Multivariate System Identification for Cerebral Autoregulation. Ann. Biomed. Eng..

[49] Placek M.M., Wachel P., Iskander D.R., Smielewski P., Uryga A., Mielczarek A., Szczepański T.A., Kasprowicz M. (2017). Applying Time-Frequency Analysis to Assess Cerebral Autoregulation during Hypercapnia. PLoS ONE.

[50] Saleem S., Teal P.D., Kleijn W.B., Ainslie P.N., Tzeng Y.-C. (2016). Identification of Human Sympathetic Neurovascular Control Using Multivariate Wavelet Decomposition Analysis. Am. J. Physiol. Heart Circ. Physiol..

[51] Smirl J.D., Haykowsky M.J., Nelson M.D., Tzeng Y.-C., Marsden K.R., Jones H., Ainslie P.N. (2014). Relationship Between Cerebral Blood Flow and Blood Pressure in Long-Term Heart Transplant Recipients. Hypertension.

[52] Tan Q., Zhang M., Wang Y., Zhang M., Wang B., Xin Q., Li Z. (2016). Age-Related Alterations in Phase Synchronization of Oxyhemoglobin Concentration Changes in Prefrontal Tissues as Measured by near-Infrared Spectroscopy Signals. Microvasc. Res..

[53] Wang B., Zhang M., Bu L., Xu L., Wang W., Li Z. (2016). Posture-Related Changes in Brain Functional Connectivity as Assessed by Wavelet Phase Coherence of NIRS Signals in Elderly Subjects. Behav. Brain Res..

[54] Zhang R., Zuckerman J.H., Giller C.A., Levine B.D. (1998). Transfer Function Analysis of Dynamic Cerebral Autoregulation in Humans. Am. J. Physiol. Heart Circ. Physiol..

[55] Asgari S., Adams H., Kasprowicz M., Czosnyka M., Smielewski P., Ercole A. (2019). Feasibility of Hidden Markov Models for the Description of Time-Varying Physiologic State After Severe Traumatic Brain Injury. Crit. Care Med..

[56] Caldas J.R., Panerai R.B., Bor-Seng-Shu E., Almeida J.P., Ferreira G.S.R., Camara L., Nogueira R.C., Oliveira M.L., Jatene F.B., Robinson T.G. (2017). Cerebral Hemodynamics with Intra-Aortic Balloon Pump: Business as Usual?. Physiol. Meas..

[57] Chiu C.-C., Yeh S.-J., Li T.-Y., Zhang D., Sonka M. (2010). Classification of Diabetics with Various Degrees of Autonomic Neuropathy Based on Linear and Nonlinear Features Using Support Vector Machine. Proceedings of the Medical Biometrics.

[58] Czosnyka M., Guazzo E., Whitehouse M., Smielewski P., Czosnyka Z., Kirkpatrick P., Piechnik S., Pickard J.D. (1996). Significance of Intracranial Pressure Waveform Analysis after Head Injury. Acta Neurochir..

[59] Daley M.L., Leffler C.W., Czosnyka M., Pickard J.D., Hoff J.T., Keep R.F., Xi G., Hua Y. (2006). Intracranial Pressure Monitoring: Modeling Cerebrovascular Pressure Transmission. Proceedings of the Brain Edema XIII.

[60] Elixmann I.M., Hansinger J., Goffin C., Antes S., Radermacher K., Leonhardt S. Single Pulse Analysis of Intracranial Pressure for a Hydrocephalus Implant. Proceedings of the 2012 Annual International Conference of the IEEE Engineering in Medicine and Biology Society.

[61] Farhadi A., Chern J.J., Hirsh D., Davis T., Jo M., Maier F., Rasheed K. (2019). Intracranial Pressure Forecasting in Children Using Dynamic Averaging of Time Series Data. Forecasting.

[62] Giller C., Lacopino D.G. (1997). Use of Middle Cerebral Velocity and Blood Pressure for the Analysis of Cerebral Autoregulation at Various Frequencies: The Coherence Index. Neurol. Res..

[63] Güiza F., Depreitere B., Piper I., Van den Berghe G., Meyfroidt G. (2013). Novel Methods to Predict Increased Intracranial Pressure During Intensive Care and Long-Term Neurologic Outcome after Traumatic Brain Injury: Development and Validation in a Multicenter Dataset*. Crit. Care Med..

[64] Han Q., Zhang M., Li W., Gao Y., Xin Q., Wang Y., Li Z. (2014). Wavelet Coherence Analysis of Prefrontal Tissue Oxyhaemoglobin Signals as Measured Using Near-Infrared Spectroscopy in Elderly Subjects with Cerebral Infarction. Microvasc. Res..

[65] Haubrich C., Diehl R.R., Kasprowicz M., Diedler J., Sorrentino E., Smielewski P., Czosnyka M., Ang B.-T. (2016). Increasing Intracranial Pressure After Head Injury: Impact on Respiratory Oscillations in Cerebral Blood Flow Velocity. Intracranial Pressure and Brain Monitoring XV.

[66] Hu K., Lo M.-T., Peng C.-K., Liu Y., Novak V. (2012). A Nonlinear Dynamic Approach Reveals a Long-Term Stroke Effect on Cerebral Blood Flow Regulation at Multiple Time Scales. PLoS Comput. Biol..

[67] Jachan M., Reinhard M., Spindeler L., Hetzel A., Schelter B., Timmer J. (2009). Parametric Versus Nonparametric Transfer Function Estimation of Cerebral Autoregulation from Spontaneous Blood-Pressure Oscillations. Cardiovasc. Eng..

[68] Kostoglou K., Wright A.D., Smirl J.D., Bryk K., van Donkelaar P., Mitsis G.D. Dynamic Cerebral Autoregulation in Young Athletes Following Concussion. Proceedings of the 2016 38th Annual International Conference of the IEEE Engineering in Medicine and Biology Society (EMBC).

[69] Kvandal P., Sheppard L., Landsverk S.A., Stefanovska A., Kirkeboen K.A. (2013). Impaired Cerebrovascular Reactivity after Acute Traumatic Brain Injury Can Be Detected by Wavelet Phase Coherence Analysis of the Intracranial and Arterial Blood Pressure Signals. J. Clin. Monit. Comput..

[70] Li W., Zhang M., Huo C., Xu G., Chen W., Wang D., Li Z. (2021). Time-Evolving Coupling Functions for Evaluating the Interaction between Cerebral Oxyhemoglobin and Arterial Blood Pressure with Hypertension. Med. Phys..

[71] Liu Q., Wang B., Liu Y., Lv Z., Li W., Li Z., Fan Y. (2018). Frequency-Specific Effective Connectivity in Subjects with Cerebral Infarction as Revealed by NIRS Method. Neuroscience.

[72] Mariak Z., Swiercz M., Krejza J., Lewko J., Lyson T. (2000). Intracranial Pressure Processing with Artificial Neural Networks: Classification of Signal Properties. Acta Neurochir..

[73] Martinez-Tejada I., Czosnyka M., Czosnyka Z., Juhler M., Smielewski P. (2021). Causal Relationship between Slow Waves of Arterial, Intracranial Pressures and Blood Velocity in Brain. Comput. Biol. Med..

[74] Megjhani M., Weiss M., Kwon S.B., Ford J., Nametz D., Kastenholz N., Fogel H., Velazquez A., Roh D., Agarwal S. (2022). Vector Angle Analysis of Multimodal Neuromonitoring Data for Continuous Prediction of Delayed Cerebral Ischemia. Neurocrit. Care.

[75] Miller E.C., dos Santos K.R.M., Marshall R.S., Kougioumtzoglou I.A. (2020). Joint Time-Frequency Analysis of Dynamic Cerebral Autoregulation Using Generalized Harmonic Wavelets. Physiol. Meas..

[76] Myers R.B., Lazaridis C., Jermaine C.M., Robertson C.S., Rusin C.G. (2016). Predicting Intracranial Pressure and Brain Tissue Oxygen Crises in Patients with Severe Traumatic Brain Injury. Crit. Care Med..

[77] Naraei P., Kenez M., Sadeghian A. A Hybrid Wavelet Based K-Means Clustering Approach to Detect Intracranial Hypertension. Proceedings of the 2017 IEEE Canada International Humanitarian Technology Conference (IHTC).

[78] Panerai R.B., Rennie J.M., Kelsall A.W.R., Evans D.H. (1998). Frequency-Domain Analysis of Cerebral Autoregulation from Spontaneous Fluctuations in Arterial Blood Pressure. Med. Biol. Eng. Comput..

[79] Petrov D., Miranda S.P., Balu R., Wathen C., Vaz A., Mohan V., Colon C., Diaz-Arrastia R. (2023). Prediction of Intracranial Pressure Crises after Severe Traumatic Brain Injury Using Machine Learning Algorithms. J. Neurosurg..

[80] Pinto H., Dias C., Rocha A.P. Multiscale Information Decomposition of Long Memory Processes: Application to Plateau Waves of Intracranial Pressure. Proceedings of the 2022 44th Annual International Conference of the IEEE Engineering in Medicine & Biology Society (EMBC).

[81] Porta A., Fantinato A., Bari V., Gelpi F., Cairo B., Maria B.D., Bertoldo E.G., Fiolo V., Callus E., Vincentiis C.D. (2020). Evaluation of the Impact of Surgical Aortic Valve Replacement on Short-Term Cardiovascular and Cerebrovascular Controls through Spontaneous Variability Analysis. PLoS ONE.

[82] Sammons E.L., Samani N.J., Smith S.M., Rathbone W.E., Bentley S., Potter J.F., Panerai R.B. (2007). Influence of Noninvasive Peripheral Arterial Blood Pressure Measurements on Assessment of Dynamic Cerebral Autoregulation. J. Appl. Physiol..

[83] Schäck T., Muma M., Feng M., Guan C., Zoubir A.M. (2018). Robust Nonlinear Causality Analysis of Nonstationary Multivariate Physiological Time Series. IEEE Trans. Biomed. Eng..

[84] Semenyutin V., Antonov V., Malykhina G., Salnikov V. (2022). Investigation of Cerebral Autoregulation Using Time-Frequency Transformations. Biomedicines.

[85] Shaw M., Hawthorne C., Moss L., Kommer M., O’Kane R., Piper I., Depreitere B., Meyfroidt G., Güiza F. (2021). Time Series Analysis and Prediction of Intracranial Pressure Using Time-Varying Dynamic Linear Models. Intracranial Pressure and Neuromonitoring XVII.

[86] Sourina O., Ang B.-T., Nguyen M.K. Fractal-Based Approach in Analysis of Intracranial Pressure (ICP) in Severe Head Injury. Proceedings of the 10th IEEE International Conference on Information Technology and Applications in Biomedicine.

[87] Swiercz M., Mariak Z., Lewko J., Chojnacki K., Kozlowski A., Piekarski P. (1998). Neural Network Technique for Detecting Emergency States in Neurosurgical Patients. Med. Biol. Eng. Comput..

[88] Swiercz M., Mariak Z., Krejza J., Lewko J., Szydlik P. (2000). Intracranial Pressure Processing with Artificial Neural Networks: Prediction of ICP Trends. Acta Neurochir..

[89] Thelin E.P., Raj R., Bellander B.-M., Nelson D., Piippo-Karjalainen A., Siironen J., Tanskanen P., Hawryluk G., Hasen M., Unger B. (2020). Comparison of High versus Low Frequency Cerebral Physiology for Cerebrovascular Reactivity Assessment in Traumatic Brain Injury: A Multi-Center Pilot Study. J. Clin. Monit. Comput..

[90] Tian F., Tarumi T., Liu H., Zhang R., Chalak L. (2016). Wavelet Coherence Analysis of Dynamic Cerebral Autoregulation in Neonatal Hypoxic–Ischemic Encephalopathy. NeuroImage Clin..

[91] Tsui F.-C., Sun M., Li C.-C., Sclabassi R.J. A Wavelet Based Neural Network for Prediction of ICP Signal. Proceedings of the 17th International Conference of the Engineering in Medicine and Biology Society.

[92] Turalska M., Latka M., Czosnyka M., Pierzchala K., West B.J., Steiger H.-J. (2009). Generation of Very Low Frequency Cerebral Blood Flow Fluctuations in Humans. Proceedings of the Acta Neurochirurgica Supplements.

[93] Wijayatunga P., Koskinen L.-O.D., Sundström N. (2022). Probabilistic Prediction of Increased Intracranial Pressure in Patients with Severe Traumatic Brain Injury. Sci. Rep..

[94] Zeiler F.A., Smielewski P., Stevens A., Czosnyka M., Menon D.K., Ercole A. (2019). Non-Invasive Pressure Reactivity Index Using Doppler Systolic Flow Parameters: A Pilot Analysis. J. Neurotrauma.

[95] Zeiler F.A., Aries M., Cabeleira M., van Essen T.A., Stocchetti N., Menon D.K., Timofeev I., Czosnyka M., Smielewski P., Hutchinson P. (2020). Statistical Cerebrovascular Reactivity Signal Properties after Secondary Decompressive Craniectomy in Traumatic Brain Injury: A CENTER-TBI Pilot Analysis. J. Neurotrauma.

[96] Zeiler F.A., Cabeleira M., Hutchinson P.J., Stocchetti N., Czosnyka M., Smielewski P., Ercole A., Anke A., Beer R., Bellander B.-M. (2021). Evaluation of the Relationship between Slow-Waves of Intracranial Pressure, Mean Arterial Pressure and Brain Tissue Oxygen in TBI: A CENTER-TBI Exploratory Analysis. J. Clin. Monit. Comput..

[97] Zhang F., Feng M., Pan S.J., Loy L.Y., Guo W., Zhang Z., Chin P.L., Guan C., King N.K.K., Ang B.T. Artificial Neural Network Based Intracranial Pressure Mean Forecast Algorithm for Medical Decision Support. Proceedings of the 2011 Annual International Conference of the IEEE Engineering in Medicine and Biology Society.

[98] Zhang F., Feng M., Loy L.Y., Zhang Z., Guan C. Online ICP Forecast for Patients with Traumatic Brain Injury. Proceedings of the 21st International Conference on Pattern Recognition (ICPR2012).

[99] Alexandrin V.V. (2010). Relationship between Myogenic Reaction and Autoregulation of Cerebral Circulation. Bull. Exp. Biol. Med..

[100] Doblar D.D., Min B.G., Chapman R.W., Harback E.R., Welkowitz W., Edelman N.H. (1979). Dynamic Characteristics of Cerebral Blood Flow Response to Sinusoidal Hypoxia. J. Appl. Physiol..

[101] Issam N., Raffaello S., Dafne S., Luigi C., Abdelkrim T. (2019). A Simple Approach to Studying Cerebral Blood Flow during Psychological Stress. Naunyn-Schmiedeberg’s Arch. Pharmacol..

[102] Zheng Y., Mayhew J. (2009). A Time-Invariant Visco-Elastic Windkessel Model Relating Blood Flow and Blood Volume. NeuroImage.

[103] Zhang R., Zuckerman J.H., Levine B.D. (1998). Deterioration of Cerebral Autoregulation during Orthostatic Stress: Insights from the Frequency Domain. J. Appl. Physiol..

[104] Dhrymes P.J., Dhrymes P.J. (1974). Cross-Spectral Analysis. Econometrics: Statistical Foundations and Applications.

[105] Gu M., Gu M. (2000). Transfer Function Analysis. Advanced Optical Imaging Theory.

[106] He L., Feng B., He L., Feng B. (2022). Time–Frequency Domain Analysis. Fundamentals of Measurement and Signal Analysis.

[107] Thakur G., Balan R., Begué M., Benedetto J.J., Czaja W., Okoudjou K.A. (2015). The Synchrosqueezing Transform for Instantaneous Spectral Analysis. Excursions in Harmonic Analysis, Volume 4: The February Fourier Talks at the Norbert Wiener Center.

[108] Tiecks F.P., Lam A.M., Aaslid R., Newell D.W. (1995). Comparison of Static and Dynamic Cerebral Autoregulation Measurements. Stroke.

[109] Zhao Y., Atlas L.E., Marks R.J. (1990). The Use of Cone-Shaped Kernels for Generalized Time-Frequency Representations of Nonstationary Signals. IEEE Trans. Acoust. Speech Signal Process..

[110] Likothanassis S.D., Demiris E.N., Procházka A., Uhlíř J., Rayner P.W.J., Kingsbury N.G. (1998). ARMAX Model Identification with Unknown Process Order and Time-Varying Parameters. Signal Analysis and Prediction.

[111] Geng K., Marmarelis V.Z. (2017). Methodology of Recurrent Laguerre–Volterra Network for Modeling Nonlinear Dynamic Systems. IEEE Trans. Neural Netw. Learn. Syst..

[112] Marmarelis V.Z. (1993). Identification of Nonlinear Biological Systems Using Laguerre Expansions of Kernels. Ann. Biomed. Eng..

[113] Kaur J., Parmar K.S., Singh S. (2023). Autoregressive Models in Environmental Forecasting Time Series: A Theoretical and Application Review. Environ. Sci. Pollut. Res..

[114] Lusia D.A., Ambarwati A. Multivariate Forecasting Using Hybrid VARIMA Neural Network in JCI Case. Proceedings of the 2018 International Symposium on Advanced Intelligent Informatics (SAIN).

[115] Banks D.L., Fienberg S.E., Meyers R.A. (2003). Data Mining, Statistics. Encyclopedia of Physical Science and Technology.

[116] Roebroeck A., Toga A.W. (2015). Granger Causality. Brain Mapping.

[117] Eddy S.R. (2004). What Is a Hidden Markov Model?. Nat. Biotechnol..

[118] Ge T., Smoller J.W., Sabuncu M.R., Wu G., Shen D., Sabuncu M.R. (2016). Chapter 2—Kernel Machine Regression in Neuroimaging Genetics. Machine Learning and Medical Imaging.

[119] Gifre-Renom L., Jones E.A.V. (2021). Vessel Enlargement in Development and Pathophysiology. Front. Physiol..

[120] Saeed N.P., Panerai R.B., Robinson T.G. (2012). Are Hand-Held TCD Measurements Acceptable for Estimates of CBFv?. Ultrasound Med. Biol..

