# Supplementary material for: Time-Series Modeling and Forecasting of Cerebral Pressure–Flow Physiology: A Scoping Systematic Review of the Human and Animal Literature
Source: Sensors (Basel). 2024 Feb 23;24(5):1453. doi: 10.3390/s24051453 (PMC10934638; doi:10.3390/s24051453)
Supplement: Supplementary file 1 [file sensors-24-01453-s001.zip › Supplemantary Appendix SA.pdf]

SUPPLEMANTARY APPENDIX SA – Search Strategy for BIOSIS

TS = (("time series analysis" OR "time series forecasting" OR "time series modelling" OR "autoregressive" OR "multivariate time series" OR "wavelet analysis" OR "heteroskedasticity" OR "fourier" OR "hidden markov model")

AND

("brain" OR "cerebral" OR "intracranial" OR "cerebrovascular")

AND

("arterio jugular oxygen content" OR "ajdo2" OR "arterio venous difference" OR "avdo2" OR "autoregulation index" OR "ari" OR "blood flow velocity" OR "brain autoregulation" OR " brain blood flow" OR "brain perfusion" OR "brain tissue oxygen tension" OR "pbto2" OR "cerebral artery flow velocity" OR "cerebral autoregulation" OR "ca" OR "cerebral blood flow" OR "cbf" OR "cerebral blood flow velocity" OR "cbfv" OR "cerebral blood volume" OR "cbv" OR "cerebral circulation" OR "cerebral flow metabolism" OR "cerebral flow metabolism" OR "cerebral homeostasis" OR "cerebral metabolic rate for oxygen" OR "cmro2" OR "cerebral metabolism" OR "cerebral microcirculation" OR "cerebral microdialysis" OR "cmd" OR "cerebral oximetry index" OR "cox" OR "cerebral perfusion" OR "cerebral perfusion pressure" OR "cpp" OR "cerebral pressure" OR "cerebral pressure autoregulation" OR "cerebral spinal reserve capacity" OR "rap" OR "cerebral vascular reactivity" OR "cerebral vascular resistance" OR "cerebral vasculature" OR "cerebral vasoconstriction" OR "cerebral vasodilation" OR "cerebral vasomotor responsiveness" OR "cerebral vasoreactivity" OR "cerebral vessel diameter" OR "cerebrovascular control" OR "cerebrovascular function" OR "cerebrovascular reactivity" OR "cortical blood flow" OR "cortical laser doppler" OR "cortical perfusion" OR "dense array electroencephalography" OR "deeg" OR "diastolic flow index" OR "dx" OR "diffuse correlation spectroscopy" OR "dcs" OR "diffusion weighted imaging index" OR "dwi" OR "dynamic autoregulatory index" OR "dax" OR "electrocorticography" OR "ecog" OR "electroencephalography" OR "eeg" OR "flow velocity" OR "fv" OR "hemoglobin volume index" OR "hbx" OR "intracranial pressure" OR "icp" OR "jugular bulb saturation" OR "sjo2" OR "jugular venous oxygen saturation" OR "svjo2" OR "kety schmidt technique" OR "kety-schmidt" OR "laser doppler flowmetry" OR "ldf" OR "mean flow index" OR "mx" OR "mean transit time" OR "mtt" OR "near infrared spectrometry" OR "near-

infrared spectrometry" OR "nirs" OR "neurovascular autoregulation" OR "neurovascular coupling" OR "neurovascular reactivity" OR "orthostatic hypotension test" OR "oht" OR "oxygen extraction fraction" OR "oef" OR "perfusion-weighted imaging index" OR "perfusion weighted imaging index" OR "pressure reactivity index" OR "prx" OR "projection pursuit regression" OR "ppr" OR "pulse amplitude of icp" OR "amp" OR "pulsatile reactivity index" OR "pax" OR "pulsatility index" OR "regional cerebral oxygen saturation" OR "rso2" OR "spatially resolved nirs" OR "stereo electroencephalography" OR "seeg" OR "systolic flow index" OR "sx" OR "thermal diffusion" OR "tdx" OR "time to peak" OR "ttp" OR "thigh cuff deflation technique" OR "tcdt" OR "transient hyperemic response test" OR "thrt" OR "tissue hemoglobin index" OR "thbx" OR "transcranial doppler" OR "tcdx" OR "tissue oxygenation index" OR "tissue oxygeneration index" OR "tox" OR "transcranial doppler sonography" OR "transfer function analysis" OR "tf" OR "tfa")

AND NOT

("Non English" OR "Non-English" OR "Cadaver Studies" OR "Theoretical studies"))
