# Supplementary material for: Time-Series Modeling and Forecasting of Cerebral Pressure–Flow Physiology: A Scoping Systematic Review of the Human and Animal Literature
Source: Sensors (Basel). 2024 Feb 23;24(5):1453. doi: 10.3390/s24051453 (PMC10934638; doi:10.3390/s24051453)
Supplement: Supplementary file 1 [file sensors-24-01453-s001.zip › Supplemantary Appendix SB.pdf]

SUPPLEMANTARY APPENDIX SB – Detailed Summary of the Systematic Review

Table of Contents

Table B1: Summary of articles for healthy human subjects with time-series analysis techniques..1
Table B2: Summary of articles for healthy human subjects with time-series models 12
Table B3: Summary of articles for healthy human subjects with model comparison...15
Table B4: Summary of articles for patient population with time-series analysis techniques 23
Table B5: Summary of articles for patient population with time-series models..30
Table B6: Summary of articles for patient population with machine learning models 33
Table B7: Summary of articles for patient population with model comparison 36
Table B8: Summary of studies for animal subjects 45

Table B1: Summary of articles for healthy human subjects with time-series analysis techniques

Article	Aim of the Study	Demographics & Experimental Conditions	Physiological Data & Measurement Methods	Data Resolution	Method(s) of Time-Series Modelling	Study Results and Conclusions Regarding Cerebral Physiologic Signal Modelling
**Frequency-Domain Analysis Studies**						
Brown et al., 2004 [1]	The effects of periodical gravitational stress on the CBFv regulations via application of LBNP were assessed.	*Subject Characteristics:*<br>Healthy subjects<br>Number of subjects: 16<br>Subject demographics: 8 male/8 female<br>Age range: 25-30 years<br>Mean age: 27 years<br><br>*Experimental conditions:*<br>The subjects refrained from caffeine and food 3 hours prior to the study. The subjects did not have any history of neurological or cardiovascular disorders. The data was recorded during steady state LBNP at -15 and -40 mmHg as well as during oscillating 0.1 (0 to -15 mmHg, LF) and 0.2 Hz (0 to -40 mmHg, HF) LBNP at supine position.	**Cerebral Physiology:**<br>CBFv was recorded via TCD ultrasonography.<br>ECG<br><br>*Other:*<br>BP was recorded with non-invasive radial arterial tonometry. $EtCO_2$ was recorded with infrared absorption via nasal cannula.<br>MABP	1.25 Hz,<br>LF: 0.03-0.14 Hz,<br>HF: 0.15-0.40 Hz	Cross-spectral analysis (for comparison of oscillating signal pairs)	• It was observed that during LF oscillatory LBNP, there was no difference in the phase shift of MABP-CBFv compared to steady-state LBNP indicating no change in the autoregulatory response despite reduced buffering ability of cerebral vessels (p-value <0.01).<br>• MABP-CBFV oscillations were approximately in phase, with a phase relation close to zero, whereas gain was lower during oscillatory LBNP but not significantly different in the HF range.<br>• Significant coherence between MABP and CBFv oscillations were observed in majority of the subjects at both LF and HF ranges, while the MABP-CBFv gain was significantly higher (p-value<0.01) for HF oscillatory LBNP suggesting further impairment of buffering ability of the cerebral vessels during high frequency fluctuations in BP.<br>• It was concluded that the regulatory effect of cerebral vessels to reduce physiological LF BP fluctuations during steady state was diminished with increasing orthostatic stress oscillations.
Katsogridakis et al., 2016 [2]	The effect of multivariate representation of dynamic CA modelling on the changes of CBFv oscillations was	*Subject Characteristics:*<br>Healthy subjects<br>Number of subjects: 30<br>Subject demographics: NA<br><br>*Experimental conditions:*<br>The subjects did not have a history of any known cardiovascular and	**Cerebral Physiology:**<br>CBFv was recorded with TCD from the MCA. CrCP and RAP were estimated from CBFv and ABP	5 Hz,<br>VLF: 0.02-0.07 Hz,<br>HF: >0.07 Hz	Welch method (for estimating power and cross-power spectral densities), multiple	• Statistically significant increase in power spectral densities was observed in the VLF with CO_2 administration (p-value<10^{-4}).<br>• Correlation between ABP and CBFv estimated by first- and second- partial

	analyzed in the VLF range.	neurological disorders. The testing was performed at a supine position. The thigh cuff method combined with administration of 5% CO_2 was followed. The subjects wore face masks for CO_2 administration. pseudorandom binary sequence fluctuations method was employed to increase ABP and $EtCO_2$ variability during simultaneous administration of CO_2.	using the first harmonic method.<br>ECG<br><br>*Other:*<br>ABP was recorded with a finger photoplethysmography device. $EtCO_2$ was measured with a capnograph connected to the face mask. HR was estimated from ECG signal.		coherence estimation with matrix approach (for input-to-output signal quantification)	coherence was observed to be weak in the VLF range and strong above that.<br>• First- and second- partial coherence estimates between $EtCO_2$ and CBFv showed a strong correlation only in the VLF range indicating strong frequency dependent characteristics between the two variables referred to CVR.<br>• It was concluded that CVR should be treated as a frequency dependent phenomenon similar to dynamic CA.
Kuo et al., 1998 [3]	The spontaneous fluctuations in the CBFv were analyzed for classification, and the underlying mechanism was examined.	<u>*Subject Characteristics:*</u><br>Healthy subjects<br>Number of subjects: 33<br>Subject demographics: 14 male/19 female<br>Age range: 22-59 years<br>Mean age: 36.7±9.7 years<br><br><u>*Experimental conditions:*</u><br>The subjects did not have a history of diabetes or cardiovascular diseases. The data was collected in supine position at rest.	**Cerebral Physiology:**<br>CBFv was recorded with TCD from the MCA.<br><br>*Other:*<br>ABP was monitored with finger plethysmography.	2 Hz,<br>VLF: 0.016-0.04 Hz,<br>LF: 0.04-0.15 Hz,<br>HF: 0.15-0.40 Hz	Cross-spectral analysis, TFA (for justification of CBFv classification in the frequency domain)	• Slow fluctuations of CBFv were classified into three groups with cross-spectral analysis and TFA.<br>• Cross-spectral analysis showed high coherence in the HF and LF ranges suggesting co-linearity between ABP and CBFv fluctuations in these two ranges.<br>• The transfer phase in the HF range was found to be significantly less than LF range (p-value<0.001).<br>• It was concluded that phase shift difference between LF and HF ranges suggested that CA would operate more efficiently in the LF range than in HF range.
Peng et al., 2008 [4]	The effects of beat-to-beat ABP fluctuations and breath-by-breath $EtCO_2$ and EtO_2 on beat-to-beat CBFv variations were assessed.	<u>*Subject Characteristics:*</u><br>Healthy subjects<br>Number of subjects: 13<br>Subject demographics: NA<br><br><u>*Experimental conditions:*</u><br>The subjects did not have any cardiovascular, respiratory, or cerebrovascular diseases. The data was recorded in supine position at rest with normal breathing.	**Cerebral Physiology:**<br>CBFv was recorded with TCD from the MCA.<br>ECG<br><br>*Other:*<br>$EtCO_2$ and EtO_2 were acquired with a mass spectrometer. ABP was recorded	1 Hz<br>LF: <0.05 Hz,<br>HF: >0.05 Hz	Multiple coherence function	• The three multiple coherences were observed to be significantly higher than the values obtained for univariate coherence of ABP-input in the low frequency whereas no significant difference was observed between multiple and univariate coherences at higher frequencies.<br>• It was concluded that at low frequencies, $EtCO_2$ and EtO_2 fluctuations on CBFv variability could be responsible from the low values of univariate coherence.

			noninvasively with finger photoplethysmography.			
**TFA Studies**						
Ainslie et al., 2007 [5]	The effects of acute hypoxia on CBF dynamics as well as the responses of integrative cardiorespiratory and cerebrovascular to acute hypoxia at rest and during exercise were examined.	*Subject Characteristics:*<br>Healthy subjects<br>Number of subjects: 14<br>Subject demographics: 6 male/8 female<br>Mean age: 25 years<br><br>*Experimental conditions:*<br>The subjects refrained from exercise and alcohol 12 hours prior and from consuming caffeine 4 hours prior to the study. The subjects were rested in supine positions during normoxia and hypoxia breathing through a leak-free respiratory mask. Following the resting state, the subjects exercised with and without induced hypoxia which was followed with a resting state with hypoxia and normoxic recovery.	**Cerebral Physiology:**<br>Δ[HbO], Δ[Hb] and Δ[HbTot] were measured by a NIRS system. CBFv was measured with TCD ultrasound. (CVRi= MBP/CBFv)<br><br>*Other:*<br>ABP was recorded with a finger photoplethysmography. $EtCO_2$ and EtO_2 were recorded with a gas analyzer. SaO_2 was measured at the finger with pulse oximetry. HR was calculated from ABP.	2 Hz, VLF: 0.02-0.07 Hz, LF: 0.07-0.20 Hz, HF: 0.20-0.30 Hz	TFA (for identifying dynamic CA), FFT (for calculating frequency-domain transforms)	• CBFv was observed to be at a higher level than expected for the given hypocapnia level during exercise suggesting increased CBF demand for a cerebral neurogenic activity (p-value<0.05).<br>• No relationship was observed between CBFv and $EtCO_2$ during hypoxic exercise indicating less sensitive CA to hypocapnia during hypoxic exercise (p-value<0.05).<br>• HbO was decreased during rest and exercise with hypoxia, while muscle oxygenation was maintained (p-value<0.05).<br>• The CA was maintained during hypoxic rest but not during hypoxic exercise (p-value<0.05).<br>• Phase shift was decreased in the LF range during hypoxic exercise (p-value<0.05).<br>• A strong relationship between the changes in CBFv and Δ[HbO] was detected during hypoxic rest whereas during hypoxic exercise, Δ[HbO] was reduced and CBFv was maintained.<br>• The results indicated CA and oxygenation could be compromised by the hypoxic exercise as it would outbalance the hypocapnia-induced CBFv lowering.
Claassen et al., 2009 [6]	The dynamic relation between ABP and CBFv was quantified by analyzing the oscillations in ABP and CBFv resulting from	*Subject Characteristics:*<br>Healthy subjects<br>Number of subjects: 8<br>Subject demographics: 4 male/4 female<br>Mean age: 30±4 years<br>Mean weight: 72±18 kg<br><br>*Experimental conditions:*	**Cerebral Physiology:**<br>CBFv was recorded via TCD ultrasonography in the MCA.<br>ECG<br><br>*Other:*	2 Hz, VLF: 0.02-0.07 Hz, LF: 0.07-0.20 Hz, HF: 0.20-0.35 Hz	TFA	• Repeated squat-stand maneuvers had larger coherence between ABP and CBFv than spontaneous oscillations for all subjects.<br>• Large changes in ABP and CBFv oscillations were observed in all frequency ranges during the repeated squat-stand maneuvers allowing a comparison of dynamic CA between low and high frequencies.<br>• It was concluded that the addition of squat-stand maneuvers to spontaneous oscillation

	squat-stand maneuvers.	12 hours prior to the study, the subjects refrained from consuming alcohol or caffeine. Data was recorded during rest in sitting position and repeated squat-stand maneuvers at various frequencies while maintaining normal breathing.	ABP was recorded with a finger photoplethysmography device. Intermittent BP was recorded with electrosphygmomanometry. $EtCO_2$ was measured with a capnography.			analysis could improve the information obtained from TFA to assess dynamic CA at VLF interval.
Iwasaki et al., 2007 [7]	The hypoxia-induced changes in CBF oscillations and the dynamic relationship of ABP and CBFv oscillations were analyzed.	*<u>Subject Characteristics:</u>*<br>Healthy subjects<br>Number of subjects: 15<br>Mean age: 22±2 years<br>Mean height: 172±5 cm<br>Mean weight: 52±5 kg<br><br>*<u>Experimental conditions:</u>*<br>A day prior to study, the subjects refrained from caffeinated or alcoholic beverages and heavy exercise. Doppler ultrasonography transducer was placed on the temporal window.<br>Data were collected in three protocols:<br>Stepwise protocol: O_2 concentration was decreased from 21% to 19%, 17% and then to 15% while the CO_2 concentration was kept constant in each level. Each O_2 concentration levels were maintained for 10 mins by hypoxic generator, and data were collected in the last 5 mins.<br>Time-control protocol: Stepwise protocol was repeated without changing the O_2 concentration levels and using 21% O_2 air instead of hypoxic air.<br>Single-dose protocol: O_2 concentration decreased from 21% to	**Cerebral Physiology:**<br>CBFv was recorded with TCD from the MCA.<br>ECG<br><br>*Other:*<br>$EtCO_2$ and SaO_2 were recorded by a pulse oximeter.<br>Intermittent BP was measured by oscillometer determination using sphygmomanometer. ABP was measured by tonometry at the right radial artery.<br>HR was measured by ECG.<br>MABP	2 Hz,<br>VLF: 0.02-0.07 Hz,<br>LF: 0.07-0.20 Hz,<br>HF: 0.20-0.35 Hz	TFA	• CBFv variability didn't change at LF but increased significantly at VLF (p-value=0.002) and HF (p-value=0.006) with hypoxia at 15% O_2.<br>• MABP variability increased at LF and HF (p-value=0.049) and increased significantly at VLF (p-value=0.008).<br>• Coherence (p-value=0.028) and transfer function gain (p-value=0.035) increased significantly in the VLF range but did not change in the LF and HF ranges with hypoxia at 15% O_2.<br>• It was concluded that ABP oscillations and the CBF oscillation dependence on the ABP oscillation were affected by the normobaric hypoxia.<br>• It was also concluded that the CBF fluctuations increased in the VLF range while the changes were significant only with hypoxia at 15% O_2 indicating a possibility of threshold for such changes.

		15% directly and data were measured for 9 of the 15 subjects.				
Oudegeest-Sander et al., 2014 [8]	The effects of ABP and $EtCO_2$ changes in different age groups on dynamic CBFv and cortical oxygenation responses were assessed.	<u>*Subject Characteristics:*</u><br>Young (Y), elderly (E), and older elderly (OE) subjects<br>Number of subjects: 20 (Y), 20 (E), 18 (OE)<br>Subject demographics: 9 male/11 female (Y), 13 male/7 female (E), 15 male/3 female (OE)<br>Age range: 21-28 years (Y), 65-69 years (E), 74-86 years (OE)<br>Mean age: 24±2 years (Y), 66±1 years (E), 78±3 years (OE)<br><br><u>*Experimental conditions:*</u><br>The subjects refrained from exercise, caffeine, and alcohol 24 hours prior to the study. The data was recorded in baseline at sitting position at rest, during repeated sit-squad maneuvers, during hypocapnia induced by heavy breathing and 5% CO_2 induced hypercapnia.	**Cerebral Physiology:**<br>CBFv was recorded with TCD from the MCA. [HbO] was recorded with NIRS.<br>(CVRi = MABP/CBFv)<br>ECG<br><br>*Other:*<br>$EtCO_2$ was acquired by a capnograph via nasal cannula. ABP was measured noninvasively with finger plethysmography. MABP	2 Hz, VLF: 0.02-0.07 Hz, LF: 0.07-0.20 Hz, HF: 0.20-0.35 Hz	TFA	• Decreased CBFv and increased CVR were observed with age (p-value<0.05 for both).<br>• In hypocapnia and hypercapnia, significant changes of $EtCO_2$ were observed in all groups (p-value<0.01) causing significant changes in MABP, CBFv, CVRi and HbO.<br>• Percentage changes in CBFv, CVRi and HbO were similar in all age groups whereas absolute changes CBFv and CVRi were higher in the young group during measurements.<br>• No differences were observed between different age groups for phase and coherence, while the gain and normalized gain observed higher in elderly compared to older elderly, and for normalized gain compared to young (p-value=0.05) in the VLF range.<br>• For the elderly group, phase shift between CBFv and HbO was found to be lower compared to young elderly (p-value=0.003).<br>• For the older elderly group, the normalized gain was slightly higher compared to the elderly group young (p-value=0.05).<br>• It was concluded that dynamic CA and CVR retained normal function while CBFv and HbO were not compromised in the older elderly group.
Panerai et al., 2021 [9]	The effects of dynamic CA and subject demographics on step responses of CrCP and RAP were examined.	<u>*Subject Characteristics:*</u><br>Healthy subjects<br>Number of subjects: 194<br>Subject demographics: 104 male/90 female<br>Age range: 20-82 years<br>Mean age: 51.7±15.2 years<br><br><u>*Experimental conditions:*</u><br>Data was extracted retrospectively from a database. The subjects did not have any cardiovascular, respiratory, or neurological diseases. The	**Cerebral Physiology:**<br>CBFv was recorded with TCD from the MCA. CrCP and RAP were estimated from BP-CBFv relationship for each cardiac cycle.<br>ECG<br><br>*Other:*	5 Hz	TFA	• A decrease in ARI was observed with increasing age in men but not in women.<br>• ARI had a strong influence (p-value<0.0001) on the temporal patterns of step responses of ABP-CBFv, ABP-CrCP, and ABP-RAP which were not influenced by sex.<br>• Step responses of CBFv and RAP were also influenced by age.<br>• It was concluded that the age affected dynamic ARI with men but not with women while the dynamic responses of RAP and CrCP to a step change in MABP were strongly influenced by ARI but not by gender.

		subjects were asked to refrain from heavy exercise and consuming alcohol caffeine, or nicotine 4 hours prior to the study. The data was recorded in supine position at rest with normal breathing.	ABP was measured by arterial volume clamping of the left middle finger. HR was derived from ECG. $EtCO_2$ was measured via nasal prongs with capnography. SBP and DBP were obtained by sphygmomanometer.			
Smirl et al., 2014 [10]	The dynamic relationship between CBF and ABP were assessed for heart transplant recipients under spontaneous conditions and during squat-stand maneuvers and compared with healthy and donor control groups.	*Subject Characteristics:*<br>Healthy group (control), heart transplant recipients (HTR), donor controls (donor)<br>Number of subjects: 9 (control), 8 (HTR), 10 (donor)<br>Subject demographics: all male<br>Mean age: 63±8 years (control), 62±8 years (HTR), 27±5 years (donor)<br><br>*Experimental conditions:*<br>The data was recorded in baseline in a seated position during resting state, and during repeated squat-stand maneuver performance at 0.05 Hz and 0.10 Hz frequencies.	**Cerebral Physiology:**<br>CBFv was recorded with TCD from the MCA.<br>(CVRi = MABP/CBFv)<br>ECG<br><br>*Other:*<br>HR was obtained from ECG. ABP was recorded with a finger plethysmography. $EtCO_2$ was measured with an online gas analyzer. MABP	4 Hz,<br>VLF: 0.02–0.07 Hz,<br>LF: 0.07–0.20 Hz	Linear TFA, power spectrum analysis (for the cross-spectrum between MABP and CBFv)	• LF gain was significantly higher for donor group compared to HTR (p-value<0.01) and control (p-value<0.01) groups.<br>• MABP and CBFv power spectrum were not different between the groups whereas significantly increased power spectrum density of MABP and CBFv was observed for all groups.<br>• In both squat-stand frequencies, there was no significant difference in TFA phase and normalized gain of any groups.<br>• It was concluded that there was no relation between cerebrovascular complications and cerebral pressure-flow dynamics after heart transplant.
Zhang et al., 1998 [11]	The effect of ABP on the spontaneous changes in CBF and the frequency-dependency of CA were assessed.	*Subject Characteristics:*<br>Healthy subjects<br>Number of subjects: 10<br>Subject demographics: 4 male/6 female<br>Mean age: 33±7 years<br>Mean height: 171±12 cm<br>Mean weight: 69±14 kg<br><br>*Experimental conditions:*	**Cerebral Physiology:**<br>CBFv was recorded with TCD from the MCA.<br>ECG<br><br>*Other:*<br>ABP was recorded with a finger	1 Hz,<br>VLF: 0.02–0.07 Hz,<br>LF: 0.07–0.20 Hz,<br>HF: 0.20–0.30 Hz	TFA	• Substantial increase in gain and gradual decrease in phase was observed with increasing frequency from LF to HF.<br>• Similar measured and predicted CBFv was observed during thigh cuff deflation implying a strong relation between the changes in CBFv and ABP in the 0.07-0.30 Hz frequency range.<br>• It was concluded that the TFA could model the short-term regulation of CBF as a result of

		The subjects did not have any cardiovascular, pulmonary, and cerebrovascular diseases. The subjects refrained from consuming caffeine or alcohol 12 hours prior to the study. The data was recorded in supine position during spontaneous uncontrolled breathing, and during two inflated and deflated thigh pressure cuffs. Only three subjects were given 5% CO_2 to induce hypercapnia during steady-state data collection.	plethysmography. Intermittent blood pressure was collected with electrosphygmomanometry. $EtCO_2$ was monitored with a mass spectrometer.			changes in ABP in the 0.07-0.30 Hz frequency range.
**Wavelet Analysis Studies**						
Addison, 2015 [12]	The stable phase coupling behavior of ABP and rSO_2 was analyzed in the time-frequency domain.	*Subject Characteristics:*<br>Healthy subject<br>Number of subjects: 1<br>Subject demographics: NA<br><br>*Experimental conditions:*<br>No information was given regarding the experimental conditions.	**Cerebral Physiology:**<br>rSO_2 was recorded with NIRS system. COx<br><br>*Other:*<br>ABP was recorded with a finger photoplethysmography device.	0.0033 Hz	Synchro-squeezed cross-wavelet transform (CWT) method (for obtaining energy related to phase term), wavelet analysis (Morlet, for obtaining phase difference map)	• Relatively constant phase difference between ABP and rSO_2 signals were indicated in the synchro-CWT plot.<br>• It was concluded that the proposed wavelet model could be utilized in analyzing relationships of complex signals to illustrate the strength of correlation between them.
Bu et al., 2016 [13]	The effect of long-term offshore work on the cerebral oxygenation oscillations were assessed.	*Subject Characteristics:*<br>Healthy sailors and control group<br>Number of subjects: 30 (sailor), 30 (control)<br>Mean age: 26.3±6.8 years (sailors), 26.1±6.4 years (control)<br>Mean height: 174.5±8.5 cm (sailor), 172.1±6.1 cm (control)	**Cerebral Physiology:**<br>Δ[HbO] was recorded by NIRS system.	2 Hz,<br>I: 0.6-2 Hz,<br>II: 0.145-0.6 Hz,<br>III: 0.052-0.145 Hz,<br>IV: 0.021-0.052 Hz,	Wavelet analysis (for evaluation of the correlation of a signal pairs), amplitude-adjusted	• The results showed that the sailors had significantly lower wavelet amplitude in the I (p-value=0.004) and III (p-value=0.034) frequency intervals compared to control group.<br>• The WPCO values in the III (p-value=0.039), IV (p-value=0.036) and V (p-value=0.03)

		Mean weight: 71.6±9.9 kg (sailor), 70.3±10.2 kg (control)<br><br>_Experimental conditions:_<br>24 hours prior to study, the subjects refrained from consuming alcohol. The subjects were free of hypertension, diabetes, subarachnoid hemorrhage and other symptoms of stroke and any disease of heart, kidney, liver, lung, etc. The fatigue level of the subjects was assessed with a short questionnaire. The data was collected in sitting position at rest.		V: 0.0095-0.021 Hz,<br>VI: 0.005-0.0095 Hz	Fourier transform (AAFT) (for calculation of mean surrogate WPCO value)	frequency intervals were significantly lower for sailors.<br>• It was concluded that the sailor fatigue could be caused by long-term offshore work leading to the insufficient oxygen supply to the brain.
Bu et al., 2017 [14]	The effects of sleep deprivation on the phase synchronization were examined to assess the physiological mechanism behind the decline in the cognitive function.	_Subject Characteristics:_<br>Healthy subjects<br>Number of subjects: 20<br>Subject demographics: 10 male/ 10 female<br>Mean age: 25.5±3.5 years<br>Mean height: 167.8±7.2 cm<br>Mean weight: 58.7±11.2 kg<br>Right-handed<br><br>_Experimental conditions:_<br>The subjects did not have any history of neurological or psychiatric diseases and were not currently on any drug treatments. 24 hours prior to the study, the subjects refrained from consuming caffeine or alcohol. Data was collected at resting, task and post-task periods for each subject as group A (without sleep deprivation) and group B (24 hours sleep deprivation). A week was allocated between the two test sessions. The subjects kept their eyes closed and relaxed during the resting state recordings. Following the task period, the subjects performed a	**Cerebral Physiology:**<br>Δ[HbO] was recorded by NIRS system.	2 Hz,<br>I: 0.6-2 Hz,<br>II: 0.145-0.6 Hz,<br>III: 0.052-0.145 Hz,<br>IV: 0.021-0.052 Hz,<br>V: 0.0095-0.021 Hz,<br>VI: 0.005-0.0095 Hz	Wavelet analysis (for evaluation of the correlation of a signal pairs), AAFT (for calculation of mean surrogate WPCO value)	• WPCO was lower in Group B in III (p-value=0.02) and V (p-value=0.037) intervals in the rest period, in III (p-value=0.029) and IV (p-value=0.039) intervals in the task period and in III (p-value=0.02) interval in the post-task recovery period.<br>• Within the vigilance task stage, the longer the reaction time and a lower accuracy rate was observed.<br>• A decline in the phase synchronization between left and right prefrontal oxyhemoglobin oscillations was observed after sleep deprivation indicating diminished cognitive function.

		vigilance task in the task stage. In the post-task recovery stage, the subjects again kept their eyes closed and remained relaxed.				
Bu et al., 2018 [15]	The phase synchronization in the resting and task states was examined with Δ[HbO] data analyzing the effects of poor sleep quality.	*Subject Characteristics:*<br>Elderly group with poor sleep quality (PSQ) and elderly healthy control group<br>Number of subjects: 15 (PSQ), 14 (control)<br>Subject demographics: 6 male (PSQ), 7 male (control)<br>Mean age: 64.67±1.72 years (PSQ), 63.36±1.74 years (control)<br><br>*Experimental conditions:*<br>The subjects did not have any neurological or psychiatric diseases. The subjects were seated comfortably with eyes closed and relaxed during the resting state recordings. Following the resting state recordings, 1-back task performances were recorded.	**Cerebral Physiology:**<br>Δ[HbO] was recorded by NIRS system.	0.08 Hz, 0.01-0.08 Hz	Wavelet analysis (Morlet)	• PSQ group had significantly lower WPCO of LPFC-RPFC (p-value=0.002), LMC-RMC (p-value=0.024), LPFC-RMC (p-value=0.017), LPFC-LOL (p-value=0.012), RPFC-LOL (p-value=0.018), LMC-LOL (p-value=0.045) and RMC–LOL (p-value=0.022) in the resting state and LPFC-RPFC (p-value<0.001), LPFC-RMC (p-value=0.022), LPFC-ROL (p-value=0.04), RPFC-LMC (p-value=0.009), RPFC-RMC (p-value=0.003) and RPFC-ROL (p-value=0.018) in the task state.<br>• In the PSQ group, the wavelet amplitude of LPFC (p-value=0.033), RPFC (p-value=0.005) and LOL (p-value=0.005) were significantly higher in the resting state. Similarly, the wavelet amplitude of LPFC (p-value=0.028), LOL (p-value=0.001) and LMC (p-value=0.01) were significantly higher in the PSQ group in the task state.<br>• The study findings indicated that the reduced phase synchronization resulting in diminished cognitive function of the subject group was caused by poor sleep quality.
Cui et al., 2014 [16]	The effects of aging on the dynamic changes in Δ[HbO] and ABP oscillations were analyzed.	*Subject Characteristics:*<br>Healthy elderly and young subjects<br>Number of subjects: 33 (elderly), 27 (young)<br>Subject demographics: 27 male/7 female (elderly), 20 male/7 female (young)<br>Mean age: 70.7±7.9 years (elderly), 25.2±3.7 years (young)<br><br>*Experimental conditions:*<br>The subjects did not have any heart diseases or smoking and drinking habits. The data were recorded in sitting position.	**Cerebral Physiology:**<br>Δ[HbO] was recorded with NIRS at the frontal lobe.<br><br>*Other:*<br>ABP was recorded by a transducer attached to the wrist.	2 Hz,<br>I (0.4-2 Hz),<br>II (0.15-0.4 Hz),<br>III (0.05-0.15 Hz),<br>IV (0.02-0.05 Hz),<br>V (0.0095-0.02 Hz),<br>VI (0.005-0.0095 Hz)	Wavelet analysis	• The study results identified significant WCPO in the I frequency interval for the elderly group (p-value=0.015).<br>• The WCO was significantly different between the elderly and young subjects in the frequency intervals I and V.<br>• The WPCO of ABP and Δ[HbO] was significant in the I, II and IV frequency intervals for the elderly subjects and in the III and VI intervals for the young subjects. It was significantly different between the two subject

						groups in the IV frequency interval (p-value=0.028).<br>• It was concluded that the difference in WPCO between the young and elderly subjects indicated an altered CA resulted by aging.
Li et al., 2014 [17]	The coherence between Δ[HbO] signals of healthy and elderly subjects with hypertension was analyzed during resting state.	<u>*Subject Characteristics:*</u><br>Normotensive control group, elderly group with hypertension<br>Number of subjects: 26 (control), 24 (elder)<br>Subject demographics: 20 male/6 female (control), 17 male/7 female (elder)<br>Mean age: 70.6±7.9 years (control), 70.7±8.4 years (elder)<br><br><u>*Experimental conditions:*</u><br>The data was collected in comfortable sitting position.	**Cerebral Physiology:**<br>Δ[HbO] and Δ[Hb] were calculated from Beer-Lambert law using signals from NIRS.	2 Hz,<br>I: 0.4 - 2 Hz,<br>II: 0.15 - 0.4 Hz,<br>III: 0.05 - 0.15 Hz,<br>IV: 0.02 - 0.05 Hz	Wavelet analysis	• Significantly high WCO of Δ[HbO] oscillations were found in intervals I (p-value=0.000) and III (p-value=0.014) for the control group, and significant variations in WCO were observed between control and elder groups in interval III (p-value=0.014).<br>• WPCO of Δ[HbO] oscillations were significant for control group in intervals from I to IV, and significant difference was observed between the subject groups in interval III (p-value=0.007).<br>• It was concluded that the lower WCO values in interval III implied a decreased neural control synchronization between left and right PFC implying weakened brain functional connectivity in the elderly group with hypertension.
Saleem et al., 2016 [18]	The role of sympathetic neurovascular control on cerebral autoregulatory dynamics was identified by assessing cerebral pressure-flow relations.	<u>*Subject Characteristics:*</u><br>Healthy subjects<br>Number of subjects: 18<br>Subject demographics: 7 male/11 female<br>Age range: 21-26 years<br><br><u>*Experimental conditions:*</u><br>The subjects advised to refrain from caffeine and heavy exercise 12 hours prior to the study. The subjects were randomly divided into two groups: control group with an oral placebo pill or active treatment group with oral 0.05 mg/kg Prazosin. The data was collected in supine position.	**Cerebral Physiology:**<br>CBFv was recorded with TCD from the MCA.<br>ECG<br><br>*Other:*<br>HR was obtained from ECG. ABP was recorded noninvasively with a finger plethysmography. $EtCO_2$ was acquired from a nasal line.	2 Hz,<br>VLF: 0.02-0.07 Hz,<br>LF: 0.07-0.20 Hz,<br>HF: 0.2-0.4 Hz	Wavelet phase synchronization analysis, TFA (to characterize cerebral BP-CBF dynamic), multiple coherence function	• A significant increase was observed with admission of sympathetic blockade in gain in the VLF range (p-value<0.05), and in coherence in the VLF and LF ranges (p-value<0.05) whereas there was no significant change between pre- and post-sympathetic blockade for phase and power spectral densities.<br>• Wavelet phase synchronization index values were increased with sympathetic blockade at VLF range (p-value<0.05) for both single- and dual-input systems whereas no change was observed with placebo administration. Additionally, it was observed that treatment responses dependent on frequency and $EtCO_2$ corrected phase synchronization index values.<br>• It was concluded that fluctuations in CBF was altered strongly at VLF by sympathetic activity.

Tan et al., 2016 [19]	The phase synchronization of Δ[HbO] signals in the left and right prefrontal tissues were analyzed.	<u>*Subject Characteristics:*</u><br>Healthy elderly and young subjects<br>Number of subjects: 43 (elderly), 40 (young)<br>Subject demographics: 22 male/21 female (elderly), 27 male/13 female (youth)<br>Mean age: 69.6±8.4 years (elderly), 24.5±1.7 years (youth)<br><br><u>*Experimental conditions:*</u><br>Subjects did not have smoking or drinking habits. Subjects were seated in a comfortable position that minimized the head and wrist movements during data measurements. Sensors were placed on the participants' forehead.	**Cerebral Physiology:**<br>The left and right prefrontal Δ[HbO] signals were recorded through NIRS.	2 Hz,<br>I: 0.6 - 2 Hz,<br>II: 0.145 - 0.6 Hz,<br>III: 0.052 - 0.145 Hz,<br>IV: 0.021 - 0.052 Hz,<br>V: 0.0095 - 0.021 Hz,<br>VI: 0.005 - 0.0095 Hz	Wavelet analysis	• The wavelet amplitude of the elderly was significantly lower in intervals III (p-value=0.001) and V (p-value=0.023) in the left prefrontal cortex, and interval III (p-value=0.028) in the right prefrontal cortex.<br>• The results showed that WPCO of the Δ[HbO] oscillations were significantly lower in the left and right prefrontal regions in the intervals 1-IV in both subject groups. In intervals I (p-value=0.010) and III (p-value=0.016), the WPCO in the elderly group was significantly lower.<br>• It was concluded that a declined cognitive performance could be induced by the weakened prefrontal functional connectivity as a result of normal aging.
Wang et al., 2016 [20]	The changes in brain functional connectivity as a result of posture change was assessed in elderly subjects.	<u>*Subject Characteristics:*</u><br>Young subjects (control), elderly subjects (elderly)<br>Number of subjects: 22 (control), 39 (elderly<br>Subject demographics: 14 male/8 female (young), 13 male/16 female (elderly)<br>Mean age: 24.4±1.6 years (young), 70.5±7.7 years (elderly)<br><br><u>*Experimental conditions:*</u><br>The data was collected in sitting position with subjects' eyes closed at rest and at standing position.	**Cerebral Physiology:**<br>Δ[HbO] was measured by a NIRS system.	2 Hz,<br>I: 0.6–2 Hz,<br>II: 0.145–0.6 Hz,<br>III: 0.052–0.145 Hz,<br>IV: 0.021–0.052 Hz,<br>V: 0.0095–0.021 Hz	Wavelet analysis	• Significant relation between posture change and age with PFC and LMC connectivity in V frequency interval (p-value=0.028).<br>• Significant variation was observed between left and RPFC in I frequency range, LMC and RMC connectivity in IV frequency ranges, and connectivity between RPFC and RMC, LPFC and LMC, RPFC and LMC in the V frequency range for elderly subjects (p-value<0.05) as a result of posture change.<br>• It was concluded that the results could be useful in examining the risk of postural instability in elderly people.

AAFT, amplitude-adjusted Fourier transform; ABP, arterial blood pressure; ARI, autoregulation index; BP, blood pressure; CA, cerebral autoregulation; CBF, cerebral blood flow; CBFv, cerebral blood flow velocity; CrCP, critical closing pressure; COx, cerebral oximetry; CVR, cerebrovascular reactivity; CVRi, cerebrovascular reactivity index; CWT, cross-wavelet transform; DBP, diastolic blood pressure; E, elderly ; ECG, electrocardiography; $EtCO_2$, end-tidal carbon dioxide; EtO_2, end-tidal oxygen; FFT, fast Fourier transform; HbO, oxyhemoglobin concentration; HF, high frequency; HR, heart rate; HTR, heart transplant recipients; Hz, Hertz; LBNP, lower body negative pressure; LF, low frequency; LMC, left sensorimotor cortical; LOL, left occipital lobe; LPFC, left prefrontal cortex; MABP, mean arterial blood pressure; MCA, middle cerebral arterial; NA, not available; NIRS, near-infrared spectroscopy; O, older; OE, older elderly; PSQ, poor sleep quality; RAP, resistance-area product; RMC, right sensorimotor cortical; RPFC, right prefrontal cortex; ROL, right occipital lobe; rSO_2, regional cerebral oxygenation; SaO_2, arterial oxygen saturation; SBP, systolic blood pressure; TCD, transcranial Doppler; TFA, transfer function analysis; VLF, very low frequency; WCO, wavelet coherence; WPCO, wavelet phase coherence; Y, young; Δ[Hb], change in deoxyhemoglobin concentration; Δ[HbO], change in oxyhemoglobin concentration; Δ[HbTot], change in total hemoglobin

Table B2: Summary of articles for healthy human subjects with time-series models

Article	Aim of the Study	Demographics & Experimental Conditions	Physiological Data & Measurement Methods	Data Resolution	Method(s) of Time-Series Modelling	Study Results and Conclusions Regarding Cerebral Physiologic Signal Modelling
**ARMA Studies**						
Clough et al., 2022 [21]	The changes in CBF and dynamic CA were analyzed with respect to step responses of CrCP and RAP during PHPV.	*Subject Characteristics:*<br>Healthy subjects<br>Number of subjects: 75<br>Subject demographics: 39 male/36 female<br>Age range: 21-82 years<br>Mean age: 52.3±17.2 years<br><br>*Experimental conditions:*<br>The data was collected following two protocols. For one of the protocols, the recordings included baseline and PHPV. In the second protocol, the recordings were applied only for baseline and hypocapnia phases.	**Cerebral Physiology:**<br>CBFv was recorded via TCD ultrasonography in bilateral MCA. CrCP and RAP were estimated from the first harmonic method via BP-CBFv relationship.<br>ECG<br><br>*Other:*<br>$EtCO_2$ was recorded with an infrared capnography. BP was recorded with a finger photoplethysmograph y device.	5 Hz	ARMA	• There was a change in CBFv, $EtCO_2$, HR and RAP with PHPV (p-value<0.01 for all) but MABP, CrCP and SBP and DBP were not affected.<br>• Time-varying ARI was significantly different in PHPV compared to poikilocapnia (p-value<0.0001).<br>• Step-responses of percent changes of RAP (SRV_{RAP}) greatly increased during PHPV compared to poikilocapnia (p-value=0.0026). The change in SRV_{CrCP} was not significant between the PHPV and poikilocapnia (p-value=0.6).<br>• The study results showed that the dynamics of RAP controlled the changes in CBFv and dynamic CA during hypocapnia.
Edwards et al., 2004 [22]	The simultaneous effects of BP_{MCA} and $EtCO_2$ on the dynamic CA and CO_2 responsiveness were analyzed.	*Subject Characteristics:*<br>Healthy subjects<br>Number of subjects: 8<br>Subject demographics: 4 male/4 female<br>Age range: 21-24 years<br><br>*Experimental conditions:*<br>24 hours prior to study, the subjects refrained from caffeine, alcohol, and heavy exercises. The data was collected at baseline state, and during hypocapnia, normocapnia and hypercapnia	**Cerebral Physiology:**<br>CBFv was recorded with TCD from the MCA. BP_{MCA} was estimated from ABP signal.<br>(CVRi = BP_{MCA}/CBFv)<br>ECG<br><br>*Other:*<br>HR was determined from ECG. ABP was recorded with an arterial tonometry	1.25 Hz	ARMA	• A reduction in magnitude of $EtCO_2$-CVRi response as well as a slowed CVRi response to BP_{MCA} were observed from hypocapnia to hypercapnia.<br>• It was found that a small reduction in $EtCO_2$ resulted in a more rapid response of CVRi to change in BP_{MCA}.<br>• It was concluded that the two-breath method with ARMA was able to detect small but important changes in CA.

		maintained with a computerized dynamic end-tidal forcing system.	non-invasively. $EtCO_2$ was continuously recorded with a mass spectrometry from a face mask.			
Panerai et al., 2012 [23]	The effect of ABP and $PaCO_2$ on CBFv response to motor stimulation was examined.	*Subject Characteristics:*<br>Healthy subjects<br>Number of subjects: 10<br>Subject demographics: 9 male/1 female<br>Age range: >45 years<br>Mean age: 62.7±7.8 years<br>Right-handed<br><br>*Experimental conditions:*<br>The subjects did not have any cardiovascular or neurological diseases. The subjects refrained from consuming alcohol, caffeine, or nicotine 12 hours prior to the study. The data was recorded in supine position during rest and during two active motor stimulation of repetitive elbow flexion and extension.	**Cerebral Physiology:**<br>CBFv was recorded with TCD from the MCA.<br>ECG<br><br>Other: ABP was measured by arterial volume clamping of the left middle finger. EtCO2 was measured by an infrared capnography.	5 Hz	ARMA	• ABP and $EtCO_2$ had similar variance contribution on CBFv responses in ipsi- (p-$value_{ABP}$=0.007 and p-$value_{EtCO2}$=0.008) and contralateral hemispheres (p-$value_{ABP}$=0.01 and p-$value_{EtCO2}$=0.03). It was concluded from the synchronized population averages that the ABP resulted in the initial sudden change in CBFv whereas influence of $EtCO_2$ was irregular.<br>• CBFv step responses to ABP were similar in data obtained during motor stimulation and baseline.<br>• The possibility of detecting and removing ABP and $PaCO_2$ from response of CBFv to motor stimulation was shown in the study in order to improve non-invasive assessment of NVC.
**ARX Studies**						
Gehalot et al., 2005 [24]	The effectiveness of utilizing beat-to-beat blood pressure time sequences as input stimuli for deriving linear model estimates of CA.	*Subject Characteristics:*<br>Healthy subjects<br>Number of subjects: 11<br>Subject demographics: 9 male/2 female<br>Mean age: 29±6 years<br><br>*Experimental conditions:*<br>The subjects were non-smokers and did not have any known medical problems. The data was collected from the subjects in supine position at rest.	**Cerebral Physiology:**<br>CBFv was recorded with TCD from the MCA.<br>ECG<br><br>*Other:*<br>MABP was measured with photo-plethysmography.	2 Hz	ARX	• Mean square error values for each subject were same regardless of the data duration that were 0.0200 for 6 min, 0.0235, 0.0263, 0.0278 and 0.0255 for all four 1.5-min datasets in all three ARX models created where CBFv was output, and inputs were either MABP or pseudo random binary input.<br>• P-values between 6-min and 1.5-min data were 0.2767 (6-min and 1st 1.5-min), 0.1790 (6-min and 2nd), 0.1486 (6-min and 3rd) and 0.2459 (6-min and 4th).<br>• P-values between the 1.5-min datasets were 0.5459 (1st and 2nd), 0.4178 (1st and 3rd),

						0.6742 (1st and 4th), 0.8069 (2nd and 3rd), 0.8829 (2nd and 4th) and 0.7060 (3rd and 4th).<br>• It was concluded that MABP would be as effective as binary input signal an input stimulus indicating its significance in estimation of linear models of CA.
Liu and Allen, 2002 [25]	CA was assessed by analyzing the relationship between variations in ABP and noise in recordings.	<u>*Subject Characteristics:*</u><br>Healthy subjects<br>Number of subjects: 11<br>Subject demographics: NA<br><br><u>*Experimental conditions:*</u><br>The subjects were free of cerebrovascular disorders. The thigh cuff technique where (negative) step change in ABP was stimulated was used in the study.	**Cerebral Physiology:**<br>CBFv was recorded with TCD from the MCA.<br><br>*Other:*<br>ABP was monitored with finger plethysmography	1 Hz	ARX	• The study findings showed that the ARX model predicted the step response successfully in the noise-free condition with high variation in ABP (R5%=92) and with low variation of ABP (R5%=98) as well as in a noisy condition with high variation in ABP (R5%=97±8) condition for normal CA.<br>• It was suggested that under noisy conditions, the accuracy and the reliability of the model predictions could be improved by manipulation of higher ABP variations.
Liu et al., 2003 [26]	The effect of manipulated spontaneous ABP changes on CBFv was examined.	<u>*Subject Characteristics:*</u><br>Healthy subjects<br>Number of subjects: 8<br>Subject demographics: 6 male/2 female<br><br><u>*Experimental conditions:*</u><br>The subjects did not have any cerebrovascular and cardiovascular diseases. The data was recorded at rest, thigh cuff and LBNP tests in supine position. Hypercapnia was induced by 5% CO_2.	**Cerebral Physiology:**<br>CBFv was recorded with TCD from the MCA.<br><br>*Other:*<br>ABP was measured non0invasively with finger plethysmography. $EtCO_2$ was acquired with an infrared capnograph connected to a face mask.	1 Hz	ARX	• Significantly different gradient of the step responses was observed between normocapnia and hypercapnia (p-value<0.001) in each and across different experiments (p-value=0.003).<br>• Between gradient and $EtCO_2$ and between $EtCO_2$ and phase shift there were strong linear relationships (p-value<0.0001 for both) suggesting that ARX model fitted to ABP and CBFv data could be used to assess CA.<br>• It was concluded that ARX model showed a strong relationship between CBFv and ABP in healthy subjects proving useful for assessment of dynamic CA status.

ABP, arterial blood pressure; ARI, autoregulation index; ARMA, autoregressive moving average; ARX, autoregressive with exogenous input; BP, blood pressure; BP_{MCA}, blood pressure corrected at the middle cerebral artery; CA, cerebral autoregulation; CBF, cerebral blood flow; CBFv, cerebral blood flow velocity; CrCP, critical closing pressure; CVRi, cerebrovascular reactivity index; DBP, diastolic blood pressure; ECG, electrocardiography; $EtCO_2$, end-tidal carbon dioxide; HR, heart rate; Hz, Hertz; LBNP, lower body negative pressure; MABP, mean arterial blood pressure; MCA, middle cerebral arterial; NA, not available; NVC, neurovascular coupling; $PaCO_2$, arterial oxygen partial pressure; PHPV, paced hyperventilation; RAP, resistance-area product; SBP, systolic blood pressure; SRV_{CrCP}, percent changes of CrCP; SRV_{RAP}, percent changes of RAP; TCD, transcranial Doppler

Table B3: Summary of articles for healthy human subjects with model comparison

Article	Aim of the Study	Demographics & Experimental Conditions	Physiological Data & Measurement Methods	Data Resolution	Method(s) of Time-Series Modelling	Model comparison	Study Results and Conclusions Regarding Cerebral Physiologic Signal Modelling
Chacon et al., 2011 [27]	The simultaneous effect of ABP and arterial CO_2 fluctuations on CBFv was modelled to assess CVR.	<u>*Subject Characteristics:*</u><br>Healthy subjects<br>Number of subjects: 16<br>Mean age: 31.8±8.5 years<br><br><u>*Experimental conditions:*</u><br>12 hours prior to the study, the subjects refrained from consuming caffeine or alcohol. The subjects were in supine position with a head elevation of 30°. The recordings included baseline measurements and measurements of breathing 5% CO_2 in air.	**Cerebral Physiology:**<br>CBFv was recorded via TCD ultrasonography.<br><br>*Other:*<br>ABP was recorded by arterial volume clamping of the digital artery non-invasively. $EtCO_2$ was measured by an infrared capnography with a face mask.	0.6-sec	Linear and non-linear AR SVM and FIR SVM models	AR SVM models performed better in describing the dynamic CA both during baseline and at hypoxia than FIR models.	• The linear models performed well in the baseline state (FIR p-value=0.044, AR p-value=0.040) while better performances were observed with non-linear models (FIR p-value=0.234, AR p-value=0.030) during 5% CO_2 breathing state.<br>• The results showed that in overall, the AR SVM models performed better than the FIR SVR models describing the dynamic CA (AR baseline linear p-value=0.0022 and non-linear p-value=0.0027, AR 5% CO_2 linear p-value=0.00098 and non-linear p-value=0.00042).
Chacón et al., 2018 [28]	The effect of step change in BP on CBFv response was modelled with linear and non-linear models to assess the dynamic CA	<u>*Subject Characteristics:*</u><br>Healthy subjects<br>Number of subjects: 45<br>Mean age: 31±12 years<br><br><u>*Experimental conditions:*</u>	**Cerebral Physiology:**<br>CBFv was recorded via TCD ultrasonography.<br>ECG<br><br>*Other:*<br>BP was recorded by arterial volume clamping of the digital artery non-invasively.<br>$EtCO_2$ was measured	2 Hz,<br>VLF: 0.02-0.07 Hz,<br>LF: 0.07-0.2 Hz	TFA, NAR SVM and NFIR SVM models	Non-linear SVM models showed higher performance in detecting the deterioration of dynamic CA than the linear TFA models.	• The NAR and NFIR SVM models (p-value<0.001) showed significantly better ability to detect the hypercapnia-induced changes in dynamic CA.<br>• Model-free ARI with NAR models (p-value=0.022) detected the hypercapnia-induced changes in dynamic CA significantly better than

		12 hours prior to the study, the subjects refrained from consuming caffeine or alcohol. The subjects were free of any neurological or cardiovascular diseases. The recordings included rest state, baseline measurements and hypercapnia state induced by breathing 5% CO_2 all in supine position.	by an infrared capnography with a face mask. HR was estimated from ECG.				ARI but not with NFIR models (p-value=0.431).<br>• Although NFIR models were faster between the non-linear models, the best performance was achieved with model-free ARI extracted with NAR SVM models.
Chacón et al., 2022 [29]	The dynamic CA was modelled via the responses of CBFv to variations in ABP to describe the changes in cerebral hemodynamics with body posture changes.	*Subject Characteristics:*<br>Healthy subjects<br>Number of subjects: 18<br>Age range: 22-44 years<br>Mean age: 27.0±6.3 years<br><br>*Experimental conditions:*<br>The subjects were free of any cardiovascular and neurological diseases. The data were recorded in standing, sitting, and laying positions.	**Cerebral Physiology:**<br>CBFv was recorded via TCD ultrasonography in both hemispheres.<br><br>*Other:*<br>ABP was measured with a finger photoplethysmography non-invasively.	0.4 Hz	FIR SVM, NFIR SVM, NARX SVM and ARX SVM	In all models, there was no significant difference in performances in any positions.	• The study results showed no significant difference between the models in any of the three positions.<br>• The p-values of the models were found as FIR ARI=0.2522, NFIR ARI=0.3201, ARX ARI=0.9683 and NARX ARI= 0.6991.<br>• The study results showed that body postures had an effect on the cerebral hemodynamics system beyond ABP-CBFv relationship while the CBF autoregulation was not affected by different postures.
Edwards et al., 2001 [30]	The independent effects of breath-by-breath changes in $EtCO_2$ and beat-	*Subject Characteristics:*<br>Healthy subjects<br>Number of subjects: 8	**Cerebral Physiology:**<br>CBFv was recorded via TCD ultrasonography in bilateral MCA. CPP was determined with	0.3 Hz, LF: <0.07 Hz, HF: 0.07-0.3 Hz	Cross-spectral analysis (one-input and one-	The ARMAX model allowed for a simultaneous solution of two	• Within the HF range, CPP and mean CBFv were observed to have strong

	by-beat changes during breathing at rest on CBF were assessed.	Subject demographics: 6 male/2 female<br><br>*Experimental conditions:*<br>24 hours prior to study, the subjects refrained from caffeine, alcohol, and heavy exercises. The data was collected in sitting position.	arterial tonometry non-invasively.<br>(CVRi = CPP/CBFv)<br><br>*Other:* $EtCO_2$ was measured with a mass spectrometry.		output) and ARMAX model (two-input and one-output)	inputs, whereas cross-spectral analysis could not discriminate between multiple input-output relationships.	coherence which was poorer in the LF range.<br>• Negative phase shift was observed suggesting that the changes in CPP affected CVRi.<br>• Cross-spectral analysis showed correlation between CPP and $EtCO_2$ in three subjects at the LF and in all subjects at the HF ranges.<br>• ARMAX illustrated that the magnitude of $EtCO_2$ to CBFv gain was significantly smaller than that of CPP to CBFv.<br>• A high coherence between $EtCO_2$ and CPP was observed suggesting the significance of their interaction in CBF regulation.
Kostoglou et al., 2014 [31]	Nonstationary characteristics of CA and cerebral hemodynamics during step hypercapnic stimulus were investigated.	*Subject Characteristics:*<br>Healthy subjects<br>Number of subjects: 8<br>Subject demographics: 0 male/8 female<br>Mean age: 27±7.1 years<br><br>*Experimental conditions:*<br>The subjects did not have any history of cardiovascular, cerebrovascular, or respiratory diseases. The subjects were tested in the follicular phase of their menstrual cycle. The subjects were positioned in semi-supine position. Data	**Cerebral Physiology:** CBFv was recorded with TCD from the MCA.<br><br>*Other:*<br>ABP was measured by finger photoplethysmography.<br>$EtCO_2$, MABP	1 Hz,<br>VLF: <0.04 Hz,<br>LF: 0.04-0.15 Hz,<br>HF: 0.15-0.30 Hz	One-input and two-input discrete-time Laguerre function model	One-input (MABP) model was shown to exhibit more time varying characteristics and had smaller forgetting factors than two-input (MABP and $EtCO_2$) model.	• $EtCO_2$ kernel gain showed an increase during hypercapnia with two-input model.<br>• It was shown that the addition of $EtCO_2$ as input resulted in reduced non-stationarity of the single-input model estimation indicating the significance of $EtCO_2$ as an input in the assessment of dynamic CA.

		was recorded in three periods; when EtO_2 was held at 88 mmHg and EtCO2 at 1.0 mmHg above subject's natural resting value, followed by hypercapnia when $EtCO_2$ was 8 mmHg above resting levels, and lastly, when $EtCO_2$ was held at its pre-hypercapnic value.					
Marmarelis et al., 2012 [32]	The effect of changes in perfusion pressure on the dynamic CFA was examined via nonlinear modelling.	<u>*Subject Characteristics:*</u><br>Healthy subjects<br>Number of subjects: 12<br>Mean age: 37±9 years<br><br><u>*Experimental conditions:*</u><br>The data was collected in supine position at rest.	**Cerebral Physiology:**<br>CBFv was recorded with TCD from the MCA.<br>ECG<br><br>*Other:*<br>ABP was recorded non-invasively with a finger plethysmography.<br>$EtCO_2$ was acquired by a mass spectrometer via nasal cannula. HR was obtained from ECG.<br>MABP	2 Hz	Nonlinear and linear one-input and two-input PDM-based model, linear Laguerre-based model, linear single-input TFA	Nonlinear two-input PDM model achieved lower prediction error.	• Significantly lower prediction error was obtained with nonlinear two-input PDM model (NMSE=40.4% over all subject cohort) compared to nonlinear single-input PDM model (NMSE=53.8%), linear single-input PDM model (NMSE=64.94%), TFA (NMSE=63.94%), and linear Laguerre-based model (NMSE=55.47%).<br>• The PDM-based models demonstrated the ability to predict the mean CBFV as output for any given set of inputs consisting of MABP and $EtCO_2$ within the scope of this study.<br>• It was concluded that robustness and physiological interpretation of nonlinear models were improved with PDMs. The importance of inclusion of $EtCO_2$ as an input for dynamic CFA analysis and utilization of nonlinear models was also highlighted.
Marmarelis et al., 2016 [33]	The effect of inclusion of HR changes as an additional input to cerebral hemodynamics	<u>*Subject Characteristics:*</u><br>Healthy subjects<br>Number of subjects: 18	**Cerebral Physiology:**<br>CBFv was recorded with TCD from the MCA.<br>ECG	2 Hz	PDMs (three-input and two-input linear and	Nonlinear three-input models achieved lower prediction error.	• Statistically significant output prediction error reduction was observed with inclusion of HR to MABP and $EtCO_2$ as an input in linear (p-value=0.005) and nonlinear (p-

	model in addition to ABP and $EtCO_2$ on CA was examined.	Subject demographics: 9 male/9 female<br>Mean age: 66.8±7.4 years<br><br>*Experimental conditions:*<br>The data was collected in supine position at rest.	*Other:*<br>ABP was recorded non-invasively with a finger plethysmography.<br>$EtCO_2$ was acquired by a capnograph via nasal cannula. HR was obtained from ECG.		nonlinear) models		value=0.00012) models compared to two-input models.<br>• Similarly, inclusion of nonlinearities resulted in further statistically significant reduction error of output prediction for both two-input (p-value=0.00016) and three-input (p-value=0.00002) models implying functional connection between HR changes and CBFv.<br>• It was concluded that the proposed models could produce subject-specific measures quantitively and therefore could serve in personalized diagnostic purposes.
Mitsis et al., 2004 [34]	The effect of ABP and $mPEtCO_2$ on CBFv variations was analyzed with multiple input-LVN methodology.	*Subject Characteristics:*<br>Healthy subjects<br>Number of subjects: 10<br>Mean age: 30.4±20.1 years<br>Mean height: 179.6±8.9 cm<br>Mean weight: 76.6±14.0 kg<br><br>*Experimental conditions:*<br>The subjects refrained from consuming food and caffeine 4 hours prior to the study.	**Cerebral Physiology:**<br>CBFv was recorded with TCD from the MCA.<br><br>*Other:*<br>$EtCO_2$ was recorded with a nasal catheter.<br>ABP was recorded noninvasively with a finger plethysmography.<br>MABP	1 Hz	LVN (one-input and two-input linear and non-linear first, second and third order models)	Two-input nonlinear third order LVN model achieved the lowest output prediction error.	• Output prediction error reduced by 6% with inclusion of $EtCO_2$ as an input in addition to MABP.<br>• Additionally, significant reduction was observed in output prediction error when nonlinear models were used by 16% with one-input models and 18% with two-input models.<br>• The third order had smaller outcome prediction error compared to the first and second order models.<br>• The lowest output prediction error was obtained with the 3rd order two-input model (NMSE(±s.d%.)=20.2±5.4%).<br>• It was concluded that $EtCO_2$ fluctuations and nonlinear interactions between MABP and $EtCO_2$ had significant effect on the CBFv variations mainly in the LF range (<0.04 Hz).
Mitsis et al., 2006 [35]	The effects of nonlinear interactions in MABP and $EtCO_2$	*Subject Characteristics:*<br>Healthy subjects<br>Number of subjects: 10	**Cerebral Physiology:**<br>CBFv was recorded with TCD from the MCA.<br>ECG	1 Hz, VLF: <0.04 Hz, LF: 0.04 – 0.15 Hz, HF:	LVN model (one-input and two-input linear and non-	Two-input nonlinear LVN model achieved the lowest output	• A significant reduction was observed in output prediction error with nonlinear models by 15%-20%

	variations on mean CBFv variations in different frequency ranges were examined.	Subject demographics: 5 male/5 female<br>Age range: years<br>Mean age: 32.1±7.3 years<br>Mean height: 169.6±11.1 cm<br>Mean weight: 68.9±13.9 kg<br><br><u>*Experimental conditions:*</u><br>The subjects did not have any cardiovascular, cerebrovascular, or pulmonary diseases. The data were recorded at baseline and during LBNP at -15, -30 and -10 mmHg in supine position.	*Other:*<br>HR was obtained from ECG. ABP was recorded noninvasively with a finger plethysmography.<br>$EtCO_2$ was acquired by a mass spectrometer via nasal cannula.<br>MABP	0.15– 0.30 Hz	linear models)	prediction errors at baseline and during LBNP.	especially at VLF range for both baseline and during LBNP.<br>• Output prediction error was further reduced by 12% to 30% with inclusion of $EtCO_2$ as an input in addition to MABP in nonlinear models.<br>• The lowest prediction error was achieved with nonlinear, two-input model at baseline (NMSE=17.3) and in different LBNP ($NMSE_{-15mmHg}$=32.6, $NMSE_{-30mmHg}$=21.1, $NMSE_{-40mmHg}$=23.2 and $NMSE_{-50mmHg}$=23.7).<br>• Significant increase of linear and nonlinear magnitude of Volterra kernels of MABP and mean CBFv was observed above -30 mmHg LBNP in the VLF range implying an impaired dynamic CA whereas reduction in the magnitude of $EtCO_2$ and mean CBFv kernels was observed in all frequencies during LBNP indicating a weakened CVR during dynamic conditions.<br>• It was concluded that the orthostatic stress resulted in impaired dynamic CA of VLF MABP variations and reduced vasomotor reactivity.
Panerai et al., 1999 [36]	The limitations of linear assumptions in modelling dynamic relationship between ABP and CBFv was analyzed, and performances of different modelling options were compared.	<u>*Subject Characteristics:*</u><br>Healthy subjects<br>Number of subjects: 47<br>Subject demographics: 27 male/20 female<br>Age range: 44-80 years<br>Mean age: 66.9±9.2 years<br><br><u>*Experimental conditions:*</u>	**Cerebral Physiology:**<br>CBFv was recorded with TCD from the MCA.<br>ECG<br><br>*Other:*<br>ABP was measured noninvasively with finger plethysmography.	5 Hz	Linear and non-linear Laguerre-Wiener method, FFT and Aaslid-Tiecks model	Among the linear models, Volterra-Wiener model had the best results.	• In training set, nonlinear Volterra-Wiener method had better performance than linear Volterra-Wiener, FFT and Aaslid-Tiecks methods (p-value<10^{-6}) while linear Volterra-Wiener method was superior to FTT and Aaslid-Tiecks model (p-value<10^{-6}).<br>• During the thigh cuff test, nonlinear Volterra-Wiener model had the worst performance while linear Volterra-Wiener model was slightly better than FFT and Aaslid-Tiecks models.<br>• It was concluded that the poor performance of nonlinear model could be related to temporal pattern of

		The subjects refrained from consuming alcohol and caffeine 12 hours prior to the study. The subjects did not have any cardiovascular diseases. The data was recorded during normal breathing rest and during thigh cuff test.					fluctuations of MABP, and the significance of linear models in routine applications was pointed out.
Panerai et al., 2004 [37]	The dynamic relationship between ABP and CBFv were modelled with TLRN, and the performances of different models were compared.	<u>*Subject Characteristics:*</u><br>Healthy subjects<br>Number of subjects: 15<br>Age range: 23-47 years<br>Mean age: 30±7 years<br><br><u>*Experimental conditions:*</u><br>The subjects did not have any cardiovascular, neurological, or autonomic nervous system diseases. The data was recorded in supine position with 30° elevated head at rest and during repeated thigh cuff maneuvers.	**Cerebral Physiology:**<br>CBFv was recorded with TCD from the MCA.<br><br>*Other:*<br>ABP was measured noninvasively with blood pressure monitor.	2 Hz	TLRN, Aaslid-Tiecks model, Laguerre-Wiener method, TFA and simple linear regression	TLRN had the lowest validation error.	• TLRN (0.64) had slightly lower validation error than Volterra-Wiener (0.66) and Aaslid-Tiecks models (0.65) and significantly lower validation error than TFA (0.69) and simple linear regression (0.81) models.<br>• It was found that step responses of CBFv from TLRN showed nonlinear behavior in ABP-CBFv relationship involving both amplitude factor and possible directional effect.
Placek et al., 2017 [38]	Non-stationarity of CA and phase shift between ABP and CBFv oscillations were	<u>*Subject Characteristics:*</u><br>Healthy subjects<br>Number of subjects: 50	**Cerebral Physiology:**<br>CBFv was recorded with TCD from the MCA.<br>ECG	5 Hz,<br>VLF: 0.02-0.07 Hz,<br>LF: 0.07-0.20 Hz,	ZAMD, TFA and ARMA	The ARMA model offered superior temporal resolution	• The stationarity hypothesis of the signals ABP, CBFv and phase shift between them was rejected in both normocapnia and hypercapnia for most of the cases. However, rejection rate

	analyzed in the time-frequency domain.	Subject demographics: 21 male/29 female<br>Age range: 18-31 years<br>Mean age: 23 years<br><br>*Experimental conditions:*<br>12 hours prior to the study, the subjects refrained from consuming alcohol or caffeine. Subjects wore a face mask for $EtCO_2$ concentration increase to achieve hypercapnia. Data were recorded at normocapnia and hypercapnia.	*Other:*<br>ABP was monitored by a cuff placed around the left middle finger at heart level. $EtCO_2$ and continuous respiratory rate were measured with the face mask connected to a standard capnograph.	HF: 0.20-0.35 Hz		compared to the ZAMD-based approach.	was lower during hypercapnia suggesting the relation between ABP and CBFv to become more stationary with disturbed CA.<br>• There was a significant increase of time frequency coherence particularly in the LF (p-value<10^{-7}) and HF (p-value<10^{-5}) and decrease of phase shift in the VLF (p-value=0.0005) and LF (p-value<10^{-7}) and unchanged phase shift in HF (p-value=0.22) with hypercapnia.<br>• Spectral phase shift derived by TFA was significantly lower in the VLF (p-value<10^{-5}) and LF (p-value<10^{-6}) and was higher in the HF (p-value=0.017) during hypercapnia.<br>• Compared to spectral estimates, phase shift estimates with ZAMD were significantly lower in the VLF (p-value=0.009) during normocapnia and in the HF (p-value=0.0012) during hypercapnia while it was higher in the LF (p-value=0.007) during hypercapnia.<br>• It was concluded that ZAMD did not perform as intended possibly due to exclusion of the nonlinear properties of CA.

ABP, arterial blood pressure; AR, autoregressive; ARI, autoregulation index; ARMAX, autoregressive moving average with exogenous input; ARX, autoregressive with exogenous input; BP, blood pressure; CA, cerebral autoregulation; CBF, cerebral blood flow; CBFv, cerebral blood flow velocity; CFA, cerebral flow autoregulation; CPP, cerebral perfusion pressure; CVR, cerebrovascular reactivity; CVRi, cerebrovascular reactivity index; ECG, electrocardiography; $EtCO_2$, end-tidal carbon dioxide; FFT, fast Fourier transform; FIR, finite impulse response; HF, high frequency; HR, heart rate; Hz, Hertz; LBNP, lower body negative pressure; LF, low frequency; LVN; Laguerre-Volterra network; MABP, mean arterial blood pressure; NA, not available; NAR, non-linear autoregressive; NARX, non-linear autoregressive with exogenous input; NFIR, non-linear finite impulse response; NMSE, normalized mean square error; PDM, principal dynamic mode; SVM, support vector machine; TCD, transcranial Doppler; TFA, transfer function analysis; TLRN, time lagged recurrent neural network; VLF, very low frequency, ZAMD, Zhao-Atlas-Marks distribution

Table B4: Summary of articles for patient population with time-series analysis techniques

Article	Aim of the Study	Demographics & Experimental Conditions	Physiological Data & Measurement Methods	Data Resolution	Method(s) of Time-Series Modelling	Study Results and Conclusions Regarding Cerebral Physiologic Signal Modelling
**Dynamic/ Frequency Domain Analysis Studies**						
Czosnyka et al., 1996 [39]	The time-dependent relationship between amplitude of the ICP pulse wave, mean values of ICP and CPP was analyzed.	*Subject Characteristics:*<br>Severe TBI patients<br>Number of subjects: 56<br>Subject demographics: 40 male/16 female<br>Age range: 6-75 years<br>Mean age: 36 years<br><br>*Experimental conditions:*<br>All subjects were mechanically ventilated.	**Cerebral Physiology:**<br>ICP was monitored either with a fiber-optic transducer or a subdural catheter.<br>CPP<br><br>*Other:* ABP was measured from the radial or dorsalis pedis artery.<br>HR	1-min	Moving correlation coefficient	• A clear correlation between the amplitude and mean ICP relationship and the correlation between amplitude and pressure coefficient for severe head injury patients was observed.<br>• The study results showed that the high ICP and low mean CPP as well as impaired tolerance to intracranial hypertension determined poor outcome.
Elixmann et al., 2012 [40]	Patient state determination was modelled through extracting and categorizing ICP signals into predefined waveforms.	*Subject Characteristics:*<br>Idiopathic normal pressure hydrocephalus patients<br>Number of subjects: 13<br>Subject demographics: NA<br><br>*Experimental conditions:*<br>No information was given regarding the experimental conditions.	**Cerebral Physiology:**<br>ICP was recorded with a tip catheter.	5.12-sec	Single pulse analysis	• The model was able to identify 5 different waveforms from increasing pressure and decreasing compliance.<br>• It was concluded that the algorithm could be suitable for future hydrocephalus implant as it did not depend on the pressure drift.
Giller and Gerardo lacopino, 1997 [41]	To assess the CA, coherence between CBFv and BP was examined.	*Subject Characteristics:*<br>SAH patients, healthy group<br>Number of subjects: 8 (patients), 6 (healthy)<br>Subject demographics: NA<br><br>*Experimental conditions:*<br>The healthy group did not have any history of cardiovascular diseases. EtCO2 monitoring was available for one patient and one normal subject.	**Cerebral Physiology:**<br>CBFv was recorded with TCD from the MCA.<br><br>*Other:*<br>BP was recorded either with an arterial catheter or with a non-invasive plethysmography device.	1 Hz	FFT	• Overall, it was observed that the random error was determined by the coherence magnitude. The random error was high for low coherence values and low for high coherence.<br>• High coherence was observed in some of the data segments in SAH patients as well as two healthy subjects suggesting a small amounts of correlation in the autoregulatory mechanisms.

Li et al., 2021 [42]	The changes in the coupling interaction between ABP and oxyhemoglob in concentration oscillations was investigated in hypertensive subjects with DBI.	*Subject Characteristics:*<br>Healthy volunteers (control), hypertensive individuals (HS)<br>Number of subjects: 30 (control), 32 (HS)<br>Subject demographics: 16 male/14 female (control), 18 male/14 female (HS)<br>Age range: years<br>Mean age: 55.1±10.6 years (control), 58.9±8.7 years (HS)<br>Right-handed<br><br>*Experimental conditions:*<br>The subjects refrained from excessive exercise and alcohol 12 hours prior to the study. The subjects overall cognitive function was unimpaired, and they did not experience subjective memory problems. fNIRS and ABP data recordings took place in resting state.	**Cerebral Physiology:**<br>Δ[HbO] and Δ[Hb] were measured with multi-channel fNIRS device in the PFC, motor cortex and occipital lobe.<br><br>*Other:*<br>ABP was recorded non-invasive blood pressure device.	2 Hz,<br>I: 0.6–2 Hz,<br>II: 0.145–0.6 Hz,<br>III: 0.01–0.08 Hz	DBI (for investigating coupling interactions between ABP and oxyhemoglobin concentration), wavelet transform.	• HS group had significantly higher coupling strength from ABP to Δ[HbO] in interval I in LMC (p-value=0. 0007), RMC (p-value=0.0008), LOL (p-value=0.00001), and ROL (p-value=0.00004) indicating a more direct response of cardiac activity in cerebral hemoglobin oscillations to the changes in systemic ABP in hypertensive individuals.<br>• Similarly, HS group had significantly higher coupling strength from ABP to Δ[HbO] in interval III in LPFC (p-value=0.016), RPFC (p-value=0.003), LMC (p-value=0.00008), RMC (p-value=0.0008), LOL (p-value=0.0007), and ROL (p-value=0.001) indicating the susceptibility of the cerebral hemoglobin oscillations to ABP changes in hypertensive subjects.<br>• No significant difference in the coupling strength from ABP to Δ[Hb] was found between the two groups in interval I while it was significantly higher for HS group in interval III in LMC (p-value=0.012) and RMC (p-value=0.008).<br>• HS group had coupling strength from ABP to Δ[HbO] negatively related to DBP in LMC (interval II and III: p-value=0.007) and RMC (interval II: p-value=0.015; interval III: p-value=0.012) and positively related to pulse pressure n LMC (interval II: p-value=0.007) and RMC (interval II and III: p-value=0.012).<br>• The study results illustrated that hypertension caused impairment of dynamic CA.
Liu et al., 2018 [43]	The effective connectivity in CI patients were analyzed in various frequency ranges through NIRS method.	*Subject Characteristics:*<br>healthy subjects (control), cerebral infarction patients (CI)<br>Number of subjects: 11 (control), 11 (CI)<br>Subject demographics: 6 male/5 female (control), 5 male/6 female (CI)<br>Mean age: 72±7.6 years (control), 65±6.3 years (CI)<br><br>*Experimental conditions:*	**Cerebral Physiology:**<br>Δ[HbO] and Δ[Hb] were calculated from Beer-Lambert law using signals from NIRS.	2 Hz,<br>I: 0.6–2 Hz,<br>II: 0.145–0.6 Hz,<br>III: 0.052–0.145 Hz,<br>IV: 0.021–0.052 Hz,<br>V: 0.0095–0.021 Hz,	DBI (for investigating coupling strength), wavelet transform	• The main coupling direction was altered significantly in interval II from the direction of RPFC to LMC (p-value=0.0036) and RMC (p-value=0.0017) and from direction of LPFC to LMC (p-value=0.0017) and RMC (p-value=0.0025), in interval IV from the direction of RPFC to LMC (p-value=0.0047) and RMC (p-value=0.0041), in interval VI in between LMC and RMC (p-value=0.032).<br>• A significant decreased coupling strength of the effective connectivity was observed in CI group

		The patients had CI for more than 12 months and no other serious physiological diseases.		VI: 0.005–0.0095 Hz		which was the most significant in intervals IV (p-value=0.0006) and VI (p-value=0.0028).<br>• A greatly decreased coupling strength in CI group as well as a shift in the coupling direction of motor section were suggested with the results of the study.
Martinez-Tejada et al., 2021 [44]	The causal relationship between oscillatory modes of ICP, ABP and CBFv was examined in hydrocephalus patients.	*<u>Subject Characteristics:</u>*<br>CSF infusion patients<br>Number of subjects: 45<br>Subject demographics: 28 male/17 female<br>Age range: 25-78 years<br>Mean age: 54 years<br><br>*<u>Experimental conditions:</u>*<br>Data was extracted retrospectively from a database. After a baseline recording, the infusion was started and terminated once a ICP plateau was observed, or the pressure exceeded 40 mmHg.	**Cerebral Physiology:**<br>CBFv was monitored with TCD.<br>ICP<br><br>*Other:*<br>ABP was recorded with a finger plethysmography.	1 Hz, IMF_6: 0.095-0.155 Hz, IMF_7: 0.052-0.094 Hz, IMF_8: 0.027-0.054 Hz, IMF_9: 0.013-0.030 Hz	Granger causality method with EEMD	• From baseline to infusion stage, the power of slow waves was observed to be increasing.<br>• Between the IMFs, no causalities were recorded during baseline phase.<br>• The most significant connection was found from CBFv to ICP in IMF_6 as 0.038 during infusion study indicating the influence of slow waves of CBFv on ICP.<br>• It was concluded that EEMD could be use in assessment of cerebral and systemic signal nonlinearity and non-stationarity.
**<u>TFA Studies</u>**						
Caldas et al., 2017 [45]	The cerebral hemodynamics of post-surgery patients with IABP, during its removal and post-removal were assessed through continuous estimates of dynamic CA and TFA.	*<u>Subject Characteristics:</u>*<br>Coronary artery bypass graft surgery patients<br>Number of subjects: 14<br>Subject demographics: 10 male/4 female<br>Mean age: 63.9±7.9 years<br><br>*<u>Experimental conditions:</u>*<br>The data recordings were performed after the surgery while the subjects were resting in a supine position. Measurements included IABP operating at one inflation every three cardiac cycle (1:3 ratio) and for IABP ON and through removal of IABP without pumping assistance (IABP OFF).	**Cerebral Physiology:**<br>CBFv was recorded with TCD from the MCA. CrCP and RAP for each cardiac cycle were obtained from the first harmonic method.<br><br>*Other:*<br>BP was recorded with invasive intra-arterial line. $EtCO_2$ was recorded with an infrared capnograph. MABP and HR were detected from the BP signals.	5 Hz	TFA	• CBFv step-response plots illustrated that the ARI values in IABP ON and OFF was not significantly different from each other (p-value = 0.42) indicating a relatively stable transition from IABP ON to OFF.<br>• During removal of IABP, the dynamic CA altered slightly, with p-value of 0.052.<br>• The findings of the study suggested that the cerebral hemodynamics of post-surgical patients with IABP operating at 1:3 mode assessed with TCD resulted in similar CBFv, ARI, CrCP and RAP values to the values obtained at baseline recordings after removal of the balloon.

Haubrich et al., 2016 [46]	The interaction between increasing ICP and R-waves in CBF in brain injury patients were examined.	*Subject Characteristics:*<br>TBI patients<br>Number of subjects: 22<br>Subject demographics: NA<br><br>*Experimental conditions:*<br>The patients had subsequently increasing ICP by at least 5 mmHg and CPP above 70 mmHg with normocapnia.	**Cerebral Physiology:**<br>ICP was measured with ICP transducer. CBFv was recorded with TCD from the MCA. RAP was calculated from ICP data.<br><br>*Other:*<br>$PaCO_2$ was monitored with a blood gas analyzer.<br>ABP	10-sec	TFA	• Diminishing R-waves transfer function gains were observed while the ICP were increasing in every patient while no correlation between R-waves and CA, CPP, and ABP was found.<br>• It was found that for RAP higher than 0.85, ICP had higher impact on transmission of R-waves to CBF.<br>• The study results showed a direct correlation between vascular and perivascular intracranial compartments indicating impact of increasing ICP on the CBFv before the alterations in cerebrovascular pulsatility or CA.
Panerai et al., 1998 [47]	The CA of term and premature neonates was characterized by analyzing the dynamic relationship between spontaneous ABP fluctuations and resulting changes in CBFv.	*Subject Characteristics:*<br>Neonates with normal or impaired CA<br>Number of subjects: 83<br>Subject demographics: NA<br><br>*Experimental conditions:*<br>The infants were not included in the study if they were admitted to neonatal ICU more than 12 hours after birth or had lethal malformation. The neonates were grouped into normal or impaired CA groups by linear regression and the coherent average method.5	**Cerebral Physiology:**<br>CBFv was measured with a TCD probe.<br><br>*Other:*<br>ABP was measured through either a peripheral or umbilical arterial catheter.	0.2-sec	TFA	• Impaired CA group had significantly higher coherences between CBFv and ABP in 0.02-0.10 Hz and 0.33-0.49 Hz frequency ranges (p-value$<10^{-6}$).<br>• Group with the normal CA had significantly more positive phase coherence between CBFv and ABP than impaired CA group.<br>• Impaired CA group had significantly higher frequency response between CBFv and ABP in 0.02-0.50 Hz frequency range (p-value=0.0009).<br>• It was concluded that different components of CA could be identified by TFA.
Sammons et al., 2007 [48]	Non-invasive and invasive recordings of ABP estimates of dynamic CA were compared.	*Subject Characteristics:*<br>Coronary intervention patients<br>Number of subjects: 27<br>Subject demographics: 26 male/1 female<br>Mean age: 61.4±11.2 years<br>Mean height: 174±5 cm<br><br>*Experimental conditions:*<br>The patients were scheduled for routine elective percutaneous coronary interventions.	**Cerebral Physiology:**<br>CBFv was measured with TCD probe.<br>ECG<br><br>*Other:*<br>ABP was recorded both noninvasively with finger plethysmography and invasively with a catheter-tip pressure transducer from aorta.	5 Hz,<br>VLF: 0.00 – 0.10 Hz,<br>LF: 0.10–0.25 Hz,<br>HF: 0.25–0.40 Hz	TFA	• There was no significant difference between total mean ABP power of non-invasive ABP and invasive ABP whereas non-invasive ABP power was significantly higher in VLF range (p-value=0.014) and significantly lower in LF range (p-value=0.02).<br>• Significantly greater non-invasive estimates of ARI index and CBFv step-response was observed.<br>• Non-invasive estimates had significantly smaller gain at frequencies <0.1 Hz and

						significantly greater phase frequency response only at frequencies >0.1 Hz.<br>• It was concluded that finger plethysmography resulted in higher values for the dynamic CA efficiency compared to aortic ABP. However, the small amplitude of the biases of gain, phase, CBFv step-response and ARI index were concluded to imply a good level agreement between the indexes of CA.
**<u>Wavelet Analysis Studies</u>**						
Han et al., 2014 [49]	The prefrontal functional connectivity in the elderly subjects with CI during resting state was assessed by analyzing Δ[HbO] data.	*<u>Subject Characteristics:</u>*<br>Elderly people with cerebral infarction (CI), healthy control<br>Number of subjects: 28 (10 elderly, 18 healthy)<br>Subject demographics: 7 male/3 female (elderly), 7 male/11 female (healthy)<br>Mean age: 74.4±9.0 years (elderly), 69.9±7.3 years (healthy)<br>Age range: 59-83 years (elderly)<br><br>*<u>Experimental conditions:</u>*<br>The subjects were made up of 10 elderly people with CI and 28 healthy people. The healthy subjects did not have any history of neurological and vascular diseases. Elderly subjects did not have diabetes mellitus, insufficiency of the heart, lungs, kidneys, and liver, smoking or drinking habits, subarachnoid hemorrhage, hypertension, and additional medications usage. The data was collected in supine position with NIRS system.	**Cerebral Physiology:**<br>Δ[HbO] and Δ[Hb] were calculated from Beer-Lambert law using signals from NIRS.	2 Hz,<br>I (0.6–2Hz), II (0.145–0.6 Hz),<br>III (0.052–0.145 Hz)<br>IV (0.021–0.052 Hz)	Wavelet analysis	• P-values for WCO in the left and right PFC were 0.290 (I), 0.081 (II), 0.003 (III) and 0.375 (IV).<br>• P-values for WPCO in the left and right PFC 0.114 (I), 0.205 (II), 0.003 (III) and 0.171 (IV).<br>• Δ[HbO] signals illustrated high coherence in the intervals I and III in the left and right PFC for the healthy subjects indicating a significant linear relationship.<br>• WCO and WPCO was significantly lower in interval III (p-value=0.003) for the elderly subjects with CI.<br>• It was concluded that the lower WCO and WPCO suggested disruption of the NVC and weakening of the resting state connectivity of the left and right PFC in the elderly subjects with CI.
Kvandal et al., 2013 [50]	The wavelet spectral energy of ICP signal	*<u>Subject Characteristics:</u>*<br>Acute TBI patients<br>Number of subjects: 22	**Cerebral Physiology:**<br>ICP was recorded with strain gauge transducer in the right frontal lobe.	2 Hz	Wavelet analysis (Morlet)	• A phase shift was observed in the patients with positive PRx in the 0.006-0.14 Hz frequency range in which CVR was found altered from

	oscillations were evaluated by analyzing ICP and ABP signals for evaluation of CA in TBI patients.	Subject demographics: 14 male/8 female<br>Mean age: 41.6±9.9 years<br><br>*Experimental conditions:*<br>The data recordings were performed in the supine position with elevated upper body between 20° and 30°. The recordings were taken on 4.3±3.7 days post-injury.	PRx was calculated from ABP and ICP.<br>ECG<br><br>*Other:*<br>ABP was recorded with a radial arterial line connected to a pressure transducer.<br>MABP			0.006 to 0.07 Hz and normal from 0.07 to 0.14 Hz.<br>• In the 0.006-2 Hz frequency interval, statistically significant WPCO was found between ICP and ABP signals (p-value<0.05).<br>• In the 0.14-1.0 Hz frequency interval, high WPCO and no phase shift was found between the ICP and ABP signals indicating the effect of heartbeat and respiration on the hydrostatic transmission to the ICP.<br>• For all patients, three district peaks were observed in 0.06-0.14 Hz frequency range as frequency peak (0.03 Hz), and in 0.14-2.0 Hz frequency range as cardiac peak (1.0 Hz) and respiratory peak (0.25 Hz).<br>• It was concluded that the spectral peaks at the cardiac, respiratory and 0.03 Hz frequencies were observed with wavelet transform of the ICP signals.
Tian et al., 2016 [51]	CA of newborns suffering from moderate to severe hypoxic ischemic encephalopat hy was assessed.	*Subject Characteristics:*<br>Neonates<br>Number of subjects: 9<br>Age range: >36 weeks<br>Mean age: 39±2 weeks<br><br>*Experimental conditions:*<br>The newborn infants had moderate to severe hypoxic ischemic encephalopathy (HIE).	**Cerebral Physiology:**<br>$SctO_2$ was recorded via an oximetry on the frontoparietal side of the head.<br><br>*Other:*<br>MABP was continuously recorded with an indwelling umbilical arterial catheter.	30-sec	Wavelet analysis	• Newborns with abnormal outcomes showed either higher in-phase coherence (p-value=0.15) or higher anti-phase coherence (p-value=0.27), and either higher in-phase gain (p-value=0.03) or anti-phase gain (p-value=0.39) than the newborns with normal outcomes.<br>• It was concluded that both in- and anti-phase coherence was related to worse clinical outcomes.<br>• The results showed that for assessment of dynamic CA of newborns with HIE during hypothermia, the wavelet coherence analysis could be used clinically.
Turalska et al., 2009 [52]	The nature of CBF at very low frequency was examined.	*Subject Characteristics:*<br>Healthy control, TBI patients<br>Number of subjects: 17 (control), 38 (patient)<br>Subject demographics: 7 male/10 female (control)<br>Mean age: 24±3 years (control)<br><br>*Experimental conditions:*	**Cerebral Physiology:**<br>CBFv was recorded with a TCD probe.<br>ICP<br><br>*Other:*<br>ABP was monitored either by finger photoplethysmography (control) or by radial	2 Hz, VLF: 0.02-0.07 Hz	Wavelet analysis	• Significantly smaller variability of ICP was observed in VLF for TBI patients (p-value<0.0001).<br>• CBFv variability was found comparable for both cohorts (p-value=0.11).<br>• Spontaneous generation of VLF oscillations within intracranial volume to compensate for reduction in ABP variability was suggested for TBI patients without cerebral hypertension.

		The control group did not have any cardiovascular, cerebrovascular, or pulmonary diseases.	artery cannulation (patients).			

ABP, arterial blood pressure; ARI, autoregulation index; BP, blood pressure; CA, cerebral autoregulation; CBF, cerebral blood flow; CBFv, cerebral blood flow velocity; CI, cerebral infarction; CPP, cerebral perfusion pressure; CrCP, critical closing pressure; CVR, cerebrovascular reactivity; DBI, dynamical Bayesian inference; ECG, electrocardiography; EEMD, ensemble empirical mode decomposition; $EtCO_2$, end-tidal carbon dioxide; fNIRS, functional near-infrared spectroscopy; HF, high frequency; HIE, hypoxic ischemic encephalopathy; HR, heart rate; HS, hypertensive; Hz, Hertz; IABP, intra-aortic balloon pump; ICP, intracranial pressure; ICU, intensive care unit; IMF, intrinsic mode functions; LF, low frequency; LPFC, left prefrontal cortex; LMC, left sensorimotor cortical; LOL, left occipital lobe; MABP, mean arterial blood pressure; MCA, middle cerebral arterial; NA, not available; NVC, neurovascular coupling; $PaCO_2$, arterial oxygen partial pressure; PFC, prefrontal cortex; RAP, resistance-area product; RMC, right sensorimotor cortical; ROL, right occipital lobe; RPFC, right prefrontal cortex; SAH, subarachnoid hemorrhage; $SctO_2$, cerebral tissue oxygen saturation; TBI, traumatic brain injury; TCD, transcranial Doppler; TFA, transfer function analysis; VLF, very low frequency; WCO, wavelet coherence; WPCO, wavelet phase coherence; Δ[Hb], change in deoxyhemoglobin concentration; Δ[HbO], change in oxyhemoglobin concentration

Table B5: Summary of articles for patient population with time-series models

Article	Aim of the Study	Demographics & Experimental Conditions	Physiological Data & Measurement Methods	Data Resolution	Method(s) of Time-Series Modelling	Study Results and Conclusions Regarding Cerebral Physiologic Signal Modelling
Daley et al., 2006 [53]	The effect of ABP and ICP on the changes on the cerebrovascular pressure transmission was examined.	*Subject Characteristics:*<br>Severe TBI patients who showed a plateau wave (Group A) and who had intracranial hypertension and hypoperfusion (Group B)<br>Number of subjects: 4 (Group A), 5 (Group B)<br>Subject demographics: NA<br><br>*Experimental conditions:*<br>No information was given regarding the experimental conditions.	**Cerebral Physiology:**<br>ICP was measured with an intraparenchymal probe.<br>CPP<br><br>*Other:*<br>ABP was recorded invasively from the radial or dorsalis pedis artery.	2 Hz	ARMA	• It was found that the highest modal frequency values of Group A decreased with increasing CPP whereas for Group B, HMF and CPP showed direct relationship.<br>• According to the study findings, pressure regulation of CBF would be intact when highest modal frequency varied opposite to CPP, and CBF would be impaired when HMF varied directly with CPP.
Pinto et al., 2022 [54]	Multivariate and simultaneous analysis of cardiocerebrovascular oscillations of R-R intervals, MABP and pulse amplitude of ICP were modelled to understand the interconnection between these signals.	*Subject Characteristics:*<br>Severe TBI patients<br>Number of subjects: 18<br>Subject demographics: 16 male/2 female<br>Mean age: 42 years<br><br>*Experimental conditions:*<br>Propofol and/or midazolam and fentanyl were used to sedate the patients who were normoventilated.	**Cerebral Physiology:**<br>ICP, CPP, ECG<br><br>*Other:* ABP, MABP, $EtCO_2$	10-sec	VARFI model	• It was shown that baseline R-R could provide information regarding an arising plateau wave possibility.<br>• All recorded phases illustrated positive values indicating the simultaneous interaction between MABP and amplitude of ICP.<br>• Long-term correlations illustrated the synergistic relation of MABP and pulse amplitude of ICP as well as giving better heart rate variability during plateau wave.<br>• It was concluded that the estimation of Transfer Entropy through VARFI model was effective in assessing the global role of long-term correlations and working reliably on short-time series.
Thelin et al., 2020 [55]	Statistical time-series relationship between ICP,	*Subject Characteristics:*<br>Mild to severe TBI patients<br>Number of subjects: 31	**Cerebral Physiology:**<br>ICP was recorded with either an intraparenchymal	10-sec and 1-min	ARIMA, VARIMA, univariate logistic	• The ARIMA model showed that ICP and MABP varied among patients whereas no significant variances were observed between 10-sec and 1-min intervals implying similar time-series behavior.

	MABP and PRx of adult TBI patients was examined.	Subject demographics: 23 male/8 female<br>Age range: 30-55 years<br>Mean age: 41.7+13.6 years<br><br>*Experimental conditions:*<br>Data was extracted retrospectively from a database.	strain gauge probe, parenchymal fiber optic pressure sensor or external ventricular drain. Long-PRx (L-PRx), which was a low frequency PRx, was derived from ICP and MABP data.<br><br>*Other:*<br>ABP was recorded with pressure transducers from either radial or femoral arterial lines.		regression analysis, Granger causality	Similarly, PRx and L-PRx were observed to be patient dependent whereas ARIMA structure of both indices were similar in individual patients.<br>• Granger causality method revealed that the MABP impacting ICP was favored by the directional nature of the relationship regardless of the time interval for most patients.<br>• Similar absolute ICP standard error changes were observed with VARIMA in overall with minute interval indicating the loss of some information on slow-wave relationships between ICP and MABP and preservation of overall shape of ICP response to MABP changes implying the retention of some information regarding CVR by the mean minute data.<br>• Univariate logistic regression analysis illustrated that there was no statistical difference between AUC of all PRx and L-PRx indices implying statistically significant relation between mortality at 6 months and L-PRx and PRx indices.
Zeiler et al., 2020 [56]	The effect of craniectomy on PRx as well as the relationship between vasogenic slow waves of ICP and MABP were assessed.	*Subject Characteristics:*<br>Moderate to severe TBI patients<br>Number of subjects: 10<br>Subject demographics: 8 male/2 female<br>Mean age: 34.0±18.0 years<br><br>*Experimental conditions:*<br>Data was extracted retrospectively from a database. The data were included from patients who underwent secondary decompressive craniectomy (DC) with high-frequency data recordings.	**Cerebral Physiology:**<br>ICP was monitored with an intraparenchymal strain gauge probe, parenchymal fiber optic pressure sensor, or external ventricular drain.<br>(CPP = MABP – ICP)<br><br>*Other:*<br>ABP was recorded with pressure transducers through either radial or femoral arterial lines. MABP	10-sec	VARIMA with IRF plots, Granger causality	• Time-series analysis and VARIMA IRF plots showed that there was no variation in the PRx time-series structure between pre- and post-DC, suggesting that secondary DC did not affect CVR.<br>• Granger causality revealed that there was no significant change in F-test value for MABP and ICP between pre- and post-DC (p-value=0.280 within the first 48-hours after DC and p-value=0.248 for beyond 48-hours after DC) indicating the minimal effect of DC on the slow-waves of ICP and MABP.<br>• It was concluded that DC did not significantly affect the PRx metrics and statistical behavior of time-series, and the change in the ICP and MABP slow-waves were also small.
Zeiler et al., 2021 [57]	The relationship between slow wave	*Subject Characteristics:*<br>Moderate to severe TBI patients<br>Number of subjects: 47<br>Mean age: 45 years	**Cerebral Physiology:**<br>ICP was monitored with an intra-parenchymal strain	10-sec	ARIMA, VARIMA with IRF,	• ICP and MABP slow waves were observed to have similar ARIMA structure whereas $PbtO_2$ displayed different optimal model structure

	fluctuations in ICP, MABP and $PbtO_2$ were investigated for derivation of CVR metrics for TBI patients.	<u>*Experimental conditions:*</u><br>Data was extracted retrospectively from a database.	gauge probe or parenchymal fiber optic pressure sensor. $PbtO_2$ was monitored with an invasive parenchymal probe.<br><br>*Other:*<br>ABP was recorded with pressure transducers through arterial lines.<br>MABP		Granger causality	implying that PbtO2 slow waves had very different behavior than that of MABP and ICP.<br>• VARIMA generated IRF plots showed that the directional relation between MABP and ICP was strong suggesting definite ICP response to MABP and limited response of $PbtO_2$ response to slow wave fluctuations of MABP or ICP.<br>• It was concluded that the ICP and MABP slow wave fluctuations were reproducible whereas there was no reliable $PbtO_2$ response to slow wave fluctuations in MABP implying that $PbtO_2$ would not be useful for deriving CVR metrics in TBI.

ABP, arterial blood pressure; ARIMA, autoregressive integrative moving average; ARMA, autoregressive moving average; AUC, area under the curve; CBFv, cerebral blood flow velocity; CPP, cerebral perfusion pressure; CVR, cerebrovascular reactivity; DC, decompressive craniectomy; ECG, electrocardiography; $EtCO_2$, end-tidal carbon dioxide; HMF, highest modal frequency; Hz, Hertz, ICP, intracranial pressure; IRF, impulse response function; L-PRx, long pressure reactivity index; MABP, mean arterial blood pressure; MCA, middle cerebral arterial; NA, not available; $PbtO_2$, cerebral tissue oxygen; PRx, pressure reactivity index; TBI, traumatic brain injury; TCD, transcranial Doppler; VARFI, vector autoregressive fractionally integrated; VARIMA, vector autoregressive integrative moving average

Table B6: Summary of articles for patient population with machine learning models

Article	Aim of the Study	Demographics & Experimental Conditions	Physiological Data & Measurement Methods	Data Resolution	Method(s) of Time-Series Modelling	Study Results and Conclusions Regarding Cerebral Physiologic Signal Modelling
Asgari et al., 2019 [58]	Cerebral dynamic states were determined from combined ICP, CPP, PRx and RAP.	<u>*Subject Characteristics:*</u><br>TBI adult patients<br>Number of subjects: 379<br>Subject demographics: 299 male/80 female<br>Mean age: 39±17 years<br><br><u>*Experimental conditions:*</u><br>Data was extracted retrospectively from a database.	**Cerebral Physiology:** ICP, CPP, PRx, RAP<br><br>*Other:* ABP	1-h	HMM	• The model predicted that lower ICP, higher CPP, intact autoregulation and preserved compensatory reserve were associated with 'good' state, while higher ICP, lower CPP, loss of autoregulation and reduced compensatory reserve were associated with 'poor' state and 'intermediate' state was associated with values between the two states (p-values<0.0001).<br>• It was observed that the HMM model the CPP values for the 'poor' state was within the published CPP management guidance despite the unsupervised learning.<br>• The results showed that the HMM model could identify the clinically relevant states unsupervised as well as its ability to identify clinically meaningful critical thresholds.
Chiu et al., 2010 [59]	Dynamic CA was assessed through the linear and non-linear features extracted from mean CBFv and MABP to classify the degrees of autonomic neuropathy in diabetic patients.	<u>*Subject Characteristics:*</u><br>Diabetics with severe autonomic neuropathy (DSN), diabetics with mild autonomic neuropathy (DMN), diabetics without autonomic neuropathy (DWN), healthy control group (control)<br>Number of subjects: 18 (DSN), 25 (DMN), 15 (DWN), 14 (control)<br>Subject demographics: 12 male/6 female (DSN), 15 male/10 female (DMN), 10 male/5 female (DWN), 4 male/10 female (control)<br>Mean age: 61.6±10.9 years (DSN), 67.5±8.8 years (DMN), 52.6±16.27 years (DWN), 30.3±8 years (control)<br><br><u>*Experimental conditions:*</u><br>The control group did not suffer from any neurological or cerebrovascular diseases. The data were recorded in supine and tilt-up positions.	**Cerebral Physiology:** CBFv was recorded with TCD from the MCA.<br><br>*Other:* ABP was measured with a finger photoplethysmography device from the right middle finger. MABP was calculated from ABP.	2 Hz, VLF: 0.015-0.07 Hz, LF: 0.07-0.15 Hz, HF: 0.15-0.40 Hz	Linear CCF-SVM, nonlinear CD-SVM	• CFF results illustrated that the correlation values for control group was generally higher than the subjects with diabetics.<br>• For DWN, DMN and control groups, the CD values of mean CBFv and MABP were significantly different in supine position compared to tilt-up position. For DSN, CD values of mean CBFv was not significantly different between the two positions.<br>• The SVM classification results showed that it was able to distinguish between the control, DSN, DMN and DWN groups with high accuracy.<br>• The overall results suggested that using SVM classifier with linear CCF features and nonlinear CD features would provide a simple, easy and non-invasive method to classify dynamic CA in diabetic patients with autonomic neuropathy.

Mariak et al., 2000 [60]	Automatic classification of the ICP waveforms in certain scale of risk classes was modelled.	*Subject Characteristics:*<br>Intracerebral hemorrhage patients<br>Number of subjects: >60<br>Subject demographics: NA<br><br>*Experimental conditions:*<br>Data was extracted retrospectively from a database.	**Cerebral Physiology:**<br>ICP was recorded before and after the surgical removal of intraparenchymal brain hematoma.	10-sec	ANN	• Unambiguous classifications were observed for some parts of the ICP signals with online classification.<br>• With classification of global properties of ICP signals, it was concluded that the uneven represented classes made the ANN classification ineffective.<br>• Classification based on global parameter extraction from ICP signals was found to be more promising than the on-Line classification of ICP signals.<br>• The authors assumed their model as not fully developed and stressed the need for further training with sufficient number of ICP samples.
Megjhani et al., 2022 [61]	The prediction of DCI using continuously updated multimodal neuromonitoring and CA analyses were examined.	*Subject Characteristics:*<br>Aneurysmal SAH patients<br>Number of subjects: 131<br>Subject demographics: 37 male/94 female<br>Mean age: 54 years<br><br>*Experimental conditions:*<br>Data was extracted retrospectively from a database. 64 of the patients had delayed cerebral ischemia (DCI).	**Cerebral Physiology:**<br>ICP, CPP, $PbtO_2$, PRx ($\Delta CPP = CPP - CPP_{Opt}$)<br><br>*Other:* ABP, MABP	60-min	TSAM algorithm	• The presented model achieved 67.3% balanced accuracy.<br>• Performance of the model was over 60% consistently after 105 hours since bleed date.<br>• It was concluded the TSAM algorithm showed potential for DCI classification using multimodal neuromonitoring and CA calculations.
Naraei et al., 2017 [62]	Normal ICP levels were predicted and distinguished from higher level ICP levels, i.e., hypertension onset and intracranial hypertension.	*Subject Characteristics:*<br>TBI patients<br>Number of subjects: 20<br>Subject demographics: NA<br><br>*Experimental conditions:*<br>The data was collected from the TBI patients during the first 24 hours of their hospitalization.	**Cerebral Physiology:**<br>ICP	1 Hz	Wavelet-based k-means clustering	• Each sample has been divided into 3 sections as normal status, intracranial hypertension onset and intracranial hypertension each in 90 second time frames. This method allowed the model to be able to differentiate the normal status from intracranial hypertension onset and intracranial hypertension.<br>• The study showed that hybrid approach of wavelet analysis and k-means clustering could effectively find intracranial hypertension without invasive measurements using.
Porta et al., 2020 [63]	Spectral and complexity analysis of characterization of cardiovascular and cerebrovascular controls as	*Subject Characteristics:*<br>Patients undergoing surgical aortic valve replacement (SAVR)<br>Number of subjects: 11<br>Subject demographics: 7 male/4 female<br>Mean age: 76±5 years<br><br>*Experimental conditions:*	**Cerebral Physiology:**<br>CBFv was measured with TCD device.<br>ECG<br><br>*Other:*	0.5 Hz, LF: 0.04-0.15 Hz, HF: 0.15-0.4 Hz	k-NN	• Vagal autonomic and baroreflex controls were found to be depressed pre-surgery and impaired post-surgery

	well as the presence of nonlinear patterns in pre- and post-surgical aortic valve replacement were examined.	The patients did not have atrial fibrillation, cerebrovascular diseases, or overt autonomic nervous system pathologies. The data was recorded a day prior to the surgery and 7 days after, in supine position and during active standing.	ABP was monitored with volume-clamp photoplethysmography.			whereas cerebrovascular variability and CA were found to be less affected by the SAVR.<br>• Complexities of SBP, DBP and MABP variabilities and nonlinear dynamics of SBP after surgery were increased as a result of autonomic control impairment.<br>• Observation of nonlinear dynamics decreased in stand position.<br>• Neither surgery nor orthostatic challenge caused a change in respiratory rate.
Shaw et al., 2021 [64]	Prediction of ICP time-series data was examined with a novel time-varying DLM.	_Subject Characteristics:_<br>TBI patients<br>Number of subjects: 106 (+155 for training)<br>Subject demographics: 80 male/26 female<br>Age range: 17.9-54.7 years<br>Mean age: 32 years<br><br>_Experimental conditions:_<br>Data was extracted retrospectively from a database.	**Cerebral Physiology:** ICP<br><br>*Other:* ABP	1-min	FASSTER time varying DLM	• The model had an overall median absolute error of 2.98 mmHg with 95% confidence intervals.<br>• An adequate accuracy was achieved with FASSTER time varying DLM illustrating its potential for ICP forecasting.<br>• Further optimizations were suggested for clinical usability of the model.
Sourina et al., 2010 [65]	For prediction of changes in health status of a patient, ICP time-series data of before and after surgery was analyzed via dynamic fractal-based method.	_Subject Characteristics:_<br>Severe TBI patients<br>Number of subjects: 9<br>Subject demographics: NA<br><br>_Experimental conditions:_<br>The data measurement methods were not mentioned. The data was recorded pre- and post-surgery.	**Cerebral Physiology:** ICP	5-sec (for 3 patients), 10-sec (for 6 patients)	Fractal analysis with box-counting and Higuchi algorithms	• Significant variability was observed in FD values of the ICP using the box-counting algorithm, both before and after decompressive craniectomy signaling the need for more aggressive clinical interventions.<br>• It was determined that critical FD values could predict changes in the clinical management stage which was validated with real-world stepwise clinical protocols in severe TBI patients.<br>• It was concluded that changes in fractal dimension values could serve as early warnings for future changes in patients' conditions, potentially aiding surgical decisions.

ABP, arterial blood pressure; ANN, artificial neural network; CA, cerebral autoregulation; CBFv, cerebral blood flow velocity; CFF, cross correlation function; CP, correlation dimension; CPP, cerebral perfusion pressure; CPP_{Opt}, optimal cerebral perfusion pressure; DBP, diastolic blood pressure; DCI, delayed cerebral ischemia; DLM, dynamic linear model; DMN, diabetics with mild autonomic neuropathy; DSN, diabetics with severe autonomic neuropathy; DWN, diabetics without autonomic neuropathy; ECG, electrocardiography; FASSTER, forecasting with additive switching of seasonality, trend and exogenous regressors; FD, fractal dimension; HF, high frequency; HMM; hidden Markov model; Hz, Hertz, ICP, intracranial pressure; k-NN, k-nearest neighbor; LF, low frequency; MABP, mean arterial blood pressure; MCA, middle cerebral arterial; NA, not available; $PbtO_2$, cerebral tissue oxygen; PRx, pressure reactivity index; RAP, resistance-area product; SAVR, surgical aortic valve replacement; SBP, systolic blood pressure; SVM, support vector machine; TBI, traumatic brain injury; TCD, transcranial Doppler; TSAM, time-varying temporal signal angle measurement; VLF, very low frequency

Table B7: Summary of articles for patient population with model comparison

Article	Aim of the Study	Demographics & Experimental Conditions	Physiological Data & Measurement Methods	Data Resolution	Method(s) of Time-Series Modelling	Model Evaluation	Study Results and Conclusions Regarding Cerebral Physiologic Signal Modelling
Farhadi et al., 2019 [66]	ICP episodes based on dynamic features of ICP, vitals and medications were forecasted with data-driven models.	<u>*Subject Characteristics:*</u><br>Pediatric ICU patients<br>Number of subjects: 78<br>Subject demographics: 42 male/36 female<br>Age range: 0-18 years<br><br><u>*Experimental conditions:*</u><br>Data was extracted retrospectively from a database.	**Cerebral Physiology:** ICP, CPP<br>Transducers were implanted in the brain parenchyma.<br><br>*Other:* MABP, HR, BP	1-min	ARIMA, ETS model, linear regression, Lasso regression, SVM and random forest	Overall, random forest had the highest accuracy.	• The results showed that the linear regression performed the worst (NMSE=4.13), the Lasso regression was the most accurate (NMSE=2.76) model by selecting and estimating the effects of relevant variables.<br>• ARIMA and ETS models performed poorly due to irregular ICP fluctuations.<br>• Random forest had the highest accuracy for forecasting ICP achieving 0.99 correlation between predicted and experimental ICP values (NMSE=0.89, and RRSE=5.7%).
Güiza et al., 2013 [67]	Prediction of increased ICP episodes and early prediction of unfavorable neurological outcome in TBI patients was assessed using dynamic characteristics of ICP and MABP.	<u>*Subject Characteristics:*</u><br>TBI patients<br>Number of subjects: 264<br>Subject demographics: 211 male/53 female<br>Age range: 19-48 years<br>Mean age: 31 years<br><br><u>*Experimental conditions:*</u><br>The patients were ICP-monitored.	**Cerebral Physiology:** ICP, CPP<br><br>*Other:* MABP	1-min	GP algorithm and logistic regression	For prediction of ICP episodes, GP model had the best overall performance compared to logistic regression which performed poorly.	• The models using dynamic information outperformed the static predictors, i.e., corticosteroid randomization after significant head injury (CRASH) and international mission for prognosis and clinical trial (IMPACT), in early neurological outcome prediction.<br>• The GP model achieved an overall good model performance in both development cohort and validation cohort (classification accuracy=77%, sensitivity=82%, and specificity=75%) for prediction of ICP episodes. The p-value of development and validation cohorts were 0.175 and 0.12, respectively.
Hu et al., 2012 [68]	The relationship between CBFv and spontaneous BP fluctuations in old adults were established	<u>*Subject Characteristics:*</u><br>Stroke patients and non-stroke subjects<br>Number of subjects: 79 (39 stroke, 40 non-stroke)	**Cerebral Physiology:**<br>CBFv was recorded with TCD from the MCA.	50 Hz	IMPFA, MMPF and TFA	IMPFA model more accurately presented the relationship	• The results showed that compared to non-stroke group, the CBFv-BP phase shift was consistently smaller for the stroke group (p-value<0.0001).<br>• The results of IMPFA suggested an active CBF regulation at multiple time

	at multiple time scales.	Subject demographics: 20 male/19 female (stroke), 17 male/23 female (non-stroke)<br>Age range: 50-80 years (stroke), 51-80 years (non-stroke)<br>Mean age: 64.6±1.4 years (stroke), 68.0±1.0 years (non-stroke)<br><br>*Experimental conditions:*<br>Subjects were studied in a supine position during the data collection. A finger photoplethysmography device was placed on the subjects' finger. Doppler probes were placed in the left and right middle cerebral arteries.	*Other:*<br>Beat-to-beat BP waveforms were measured with the finger photoplethysmograph y device. $EtCO_2$ was recorded via a face mask.			between BP and CBFv oscillations and accounted better for the non-stationarities and noise in the data recordings compared to MMPF and TFA.	scales by presenting the CBFv oscillation phase being advanced compared to BP oscillation at 0.02-0.38 Hz.<br>• It was discovered that the multiscale regulation was affected by ischemic stroke in a long-term altering the CBF regulation in infracted and non-fracted hemispheres.<br>• The results of this study pointed out that the importance of a reliable and non-invasive CBF regulation monitoring for management and daily care of stroke patients due to the long-term effect of stroke.
Jachan et al., 2009 [69]	The dynamic CA assessment using parametric transfer function estimation with non-invasively recorded spontaneous oscillations was examined.	*Subject Characteristics:*<br>Unilateral (set 1) and bilateral (set 2) impaired patients<br>Number of subjects: 91 (set 1), 44 (set 2)<br>Subject demographics: 77 male/14 female (set 1), 38 male/6 female (set 2)<br>Age range: 35-85 years (set 1), 43-84 years (set 2)<br>Mean age: 65±10 years (set 1), 71±10 years (set 2)	**Cerebral Physiology:**<br>CBFv was recorded with TCD from the MCA.<br><br>*Other:*<br>ABP was recorded with finger plethysmograph.	2.5 Hz	ARMAX model, VAR model, and non-parametric transfer function estimator	No difference between performance s of the three methods were observed while ARMAX model showed the lowest complexity on average.	• In set 1, there was no significant difference between the ARMAX, VAR and nonparametric models in the mean phase estimates for healthy (p-value=0.279) and impaired (p-value=0.450) sides. The healthy side had significantly higher phase parameter in all methods than impaired side (p-value<0.0002).<br>• Significant correlations between first and second half measurements in set 2 were found in all three methods (p-value<.0016). A very low variability between the measurements were found for set 1, while the higher variability was

		Experimental conditions:<br>The patients had severe unilateral or bilateral internal carotid artery stenosis or occlusion. The data recordings were performed in the supine position with 50° inclination of the upper body.					present for set 2 similar for coefficients of variation.<br>• It was concluded that the proposed parametric approaches could be alternative to the nonparametric method to assess CA automatically with lower model complexity.
Kostoglou et al., 2016 [70]	CA of the young athletes who experienced concussion was examined through modeling the relationship between MABP, $EtCO_2$, CBFv and visual stimulation.	*Subject Characteristics:*<br>Players who experienced concussion<br>Number of subjects: 7<br>Subject demographics: 7 male/0 female<br><br>*Experimental conditions:*<br>The subjects were tested on tasks involving two separate visuals paradigms which were reading an article relevant to the subject cohort and on-screen subject search. MCA and PCA were insonated on the right side and left sides of the brain, respectively. The testing was repeated at 72 hours, 2 weeks, and 1-month post-injury.	**Cerebral Physiology:**<br>CBFv was monitored with TCD.<br>PCAv, ECG<br><br>*Other:*<br>$EtCO_2$ was recorded with a mouthpiece.<br>BP was recorded with finger photoplethysmography.<br>MABP	1 Hz,<br>VLF: 0.005-0.04 Hz,<br>LF: 0.04-0.15 Hz,<br>HF: 0.15-0.30 Hz	ARX, and impulse response model based on LET	Significantly higher predictive performance was observed with LET models with infinite-step ahead prediction whereas with one-step ahead prediction, performances of both models were comparable.	• Two models had consistent gain and phase of MABP indicating more accurate estimation compared to those from $EtCO_2$ and visual stimulation.<br>• ARX (NMSE=0.077) and LET (NMSE=0.090) showed similar performance when their output was PCAv as visual cortex was affected directly by PCA.<br>• Statistically significant changes were observed between the testing in 72 hours and 1-month (p-value<0.0358) indicating a recovery in the CA function for most of the subjects.<br>• The study results illustrated that changes in CA of young athletes who experienced concussion was successfully detected with features from the LET and ARX models.
Miller et al., 2020 [71]	Joint time-frequency domain analysis was examined to quantify dynamic CA performance.	*Subject Characteristics:*<br>Healthy group, unilateral impaired patients<br>Number of subjects: 55 (healthy), 35 (patients)	**Cerebral Physiology:**<br>CBFv was monitored with a TCD probe.<br><br>*Other:*	0.15 Hz.	TFA, GHW and wavelet transform (Morlet)	The GHW model achieved higher sensitivity compared to other models	• In the healthy group, both GHW and TFA showed similar outcomes.<br>• In patient group, GHW achieved much higher sensitives as 74% in coherence threshold approach (p-value=0.0027) and 71.4% in coherence-weighted approach (p-value=0.0009) compared to

		Subject demographics: NA<br><br>*Experimental conditions:*<br>The patients had unilateral internal carotid artery stenosis or occlusion. The data recordings took place in supine position.	ABP was recorded with a finger plethysmography.			for impaired dynamic CA identification.	TFA and wavelet-based models in identifying expected side-to-side differences.<br>• It was concluded that the GHW analysis had better performance in terms of identifying asymmetry of dynamic CA in two cerebral hemispheres with unilateral carotid stenosis patients.
Myers et al., 2016 [72]	Predictions of intracranial hypoxia and tissue hypoxia crises were assessed for development of early warning algorithm in cases of impending crises in TBI patients on 30-min interval.	*Subject Characteristics:*<br>Severe TBI patients<br>Number of subjects: 817 (ICP prediction), 242 (brain hypoxia prediction)<br>Subject demographics: 694 male/123 female (ICP prediction), 206 male/36 female (brain hypoxia prediction)<br>Age range: 22-42 years (ICP prediction), 23-44 years (brain hypoxia prediction)<br>Mean age: 30 years<br><br>*Experimental conditions:*<br>Data was extracted retrospectively from a database.	**Cerebral Physiology:**<br>ICP was recorded with either an extra-ventricular drain or an intraparenchymal fiberoptic probe. $PbtO_2$ was measured using Licox.<br><br>*Other:*<br>MABP, $EtCO_2$, SaO_2	36-sec	GP, logistic regression, and AR-OR model	The best performances were achieved by two-state AR-OR model in both ICP and $PbtO_2$ crisis predictions.	• ICP and changes in ICP were found to be the most associated signals for elevated ICP prediction (AUC=0.85), whereas on their own CPP, EtCO2, and MABP were the least associated signals (AUC = 0.49, 0.57, and 0.58, respectively).<br>• Similarly, $PbtO_2$ and changes in $PbtO_2$ were the most associated signals in depressed $PbtO_2$ prediction (AUC=0.91) while the SaO_2 and CPP, in isolation, were the least associated signals (AUC=0.53).<br>• The two-state AR-OR model achieved AUC of 0.85 and 0.91 for ICP and $PbtO_2$ crisis predictions, respectively, with 30-min advance warning.<br>• It was concluded that the presented algorithms could provide predictions of intracranial hypertension and tissue hypoxia crises accurately and timely using the relevant signal and the time since last crisis.
Petrov et al., 2023 [73]	Prediction of onset ICP crises based on time-series data of ICP signals was modelled to be applied in preventative	*Subject Characteristics:*<br>Severe TBI patients<br>Number of subjects: 36<br>Subject demographics: NA<br><br>*Experimental conditions:*	**Cerebral Physiology:**<br>ICP was monitored invasively.	1-sec	Random forest, XGBoost and LGBM	The highest performance was achieved by the random forest model on both	• The study results showed that the random forest achieved the highest performance on all data sets with accuracy range of 0.82 to 0.88.<br>• Validation test also showed that random forest had high precision (0.76) and overall strong predictive

	therapies using machine learning algorithms in 10-min and 20-min intervals.	Data was extracted retrospectively from a database.				training and testing sets.	performance (F1-score=0.57, accuracy=0.86).<br>• It was concluded that an accurate and precise prediction of ICP crisis events from ICP time-series data was achieved.
Scalzo et al., 2012 [74]	The effectiveness of ensemble classifiers in temporal prediction of intracranial hypertension were tested on 1-min to 10-min intervals.	<u>Subject Characteristics:</u><br>Patients with ICP related conditions<br>Number of subjects: 30<br>Subject demographics: NA<br><br><u>*Experimental conditions:*</u><br>Data was extracted retrospectively from a database. The patients were treated for various ICP related conditions	**Cerebral Physiology:**<br>ECG, ICP	40 Hz	Multiple linear regression, AdaBoost and ExtraTrees	The best performance was achieved by ExtraTrees followed by AdaBoost and multilinear classifier.	• ExtraTrees showed improved results with increased input length ($AUC_{1\text{-}min}$=0.96, $AUC_{3\text{-}min}$=0.91, $AUC_{6\text{-}min}$=0.87) followed by AdaBoost ($AUC_{1\text{-}min}$=0.93, $AUC_{3\text{-}min}$=0.84, $AUC_{6\text{-}min}$=0.80) and linear classifier ($AUC_{1\text{-}min}$=0.87, $AUC_{3\text{-}min}$=0.78, $AUC_{6\text{-}min}$=0.71). ExtraTrees also had the highest sensitivities for time-to-onset of 1-min (0.93), 3-min (0.83) and 6-min (0.75).<br>• The sensitivity and specificity results obtained by linear model were significantly improved with ensemble classifiers.
Schäck et al., 2018 [75]	A new method was proposed for nonlinear causality analysis of multivariate time-series of physiological data.	<u>*Subject Characteristics:*</u><br>TBI patients<br>Number of subjects: 10<br>Subject demographics: NA<br><br><u>*Experimental conditions:*</u><br>Data was extracted retrospectively from a database.	**Cerebral Physiology:**<br>ICP, $PbtO_2$<br><br>*Other:*<br>MABP	0.1 Hz	Robust time-varying generalized partial directed coherence with Kalman filter and DEKF	DEKF and the proposed model had similar accuracy, however, proposed model had shorter computation time.	• The proposed model was shown to simultaneously detect linear and nonlinear causality between time-series signals.<br>• Although both models had similar accuracy, the proposed model had far less computation time compared to DEKF.<br>• The proposed model shown robustness against artifacts and outliers while reconstructing causality spectrum patterns in TBI data.
Semenyutin et al., 2022 [76]	State of CA was aimed to be determined in real-time with CWT and STFT models.	<u>*Subject Characteristics:*</u><br>Healthy group (control), patients with brain arteriovenous malformations (AVM), patients with arterial stenosis (AS)<br>Number of subjects: 9 (control), 6 (AVM), 6 (AS)	**Cerebral Physiology:**<br>CBFv was recorded in both MCAs with a TCD probe.<br><br>*Other:*<br>ABP was monitored with a finger plethysmography.	0.01-sec	CWT and STFT	CWT achieved higher sensitivity to changes in CA and localized the time and frequency changes	• CWT method was found more sensitive according to the sensitivity analysis of the changes of phase shift.<br>• A greater relative decrease of phase shift was observed on the left (p-value=0.0222) and the right (p-value=0.014) for CWT in hypercapnia.<br>• A greater relative increase of phase shift was observed on the left (p-

		Age range: 19-35 years (control), 41-63 years (AVM), 52-72 years (AS)<br><br>*Experimental conditions:*<br>The patients either had brain with arteriovenous malformations or brachiocephalic artery stenosis. The data measurements took place in supine position under normocapnia (5% CO_2) and hypocapnia.				better than SFTF.	value=0.035) and the right (p-value=0.041) for CWT in hypocapnia.<br>• It was concluded that the CWT model enabled enhancing non-invasiveness in real time, assessing state of a patient in the norm and identifying CA disorders in group of patients.
Swiercz et al., 1998 [77]	Prediction of ICP trends and detection of unfavorable symptom configuration in neurosurgical patients was assessed.	*Subject Characteristics:*<br>Patients with intracerebral hemorrhage or brain tumor<br>Number of subjects: >60<br>Subject demographics: NA<br><br>*Experimental conditions:*<br>Data was extracted retrospectively from a database.	**Cerebral Physiology:** ICP	10-sec	ANN, ARX and Kalman filtering	ANN was observed to have better prediction accuracy than traditional ARX predictors and Kalman filtering.	• ANN was proved to be a good tool for modelling nonlinear and non-stationary processes generating the ICP signal.<br>• It was suggested that the accuracy of the ANN predictor could be improved with further ICP recordings.
Swiercz et al., 2000 [78]	The efficiency of the neural models combined with newer signal processing algorithms was assessed in prediction of on-line ICP values	*Subject Characteristics:*<br>Patients with intracerebral hemorrhage or TBI<br>Subject demographics: NA<br><br>*Experimental conditions:*	**Cerebral Physiology:** ICP	10-sec	ANN with wavelet decompositio n and AR with Kalman filtering	ANN combined with wavelet decompositi on to pre-process the ICP data achieved the best prediction accuracy.	• ANN had better performance (arv coefficient of 0.62, MAE=3.21%) than AR with Kalman filtering (arv coefficient of 0.75, MAE=3.72%).<br>• The results showed that the ANN model was able to predict changes in ICP quite well except for the rapid changes in ICP.

	on 10-sec intervals.	Data was extracted retrospectively from a database.					
Tsui et al., 1995 [79]	ICP was predicted in both long term and short term in 32-sec, 64-sec and 5-min intervals.	<u>*Subject Characteristics:*</u><br>TBI patients<br>Number of subjects: NA<br>Subject demographics: NA<br><br><u>*Experimental conditions:*</u><br>Data was extracted retrospectively from a database.	**Cerebral Physiology:**<br>ICP	2-sec	MDP and RNN	ICP prediction by MDP provided better prediction performance and prediction accuracy than RNN.	• MDP model predicted ICP dynamics and trained in 20 minutes with 32-sec and 64-sec resolutions compared to the RNN model which couldn't capture the natural dynamics in ICP data and completed training with 5-min data resolution approximately in a day.
Wijayatunga et al., 2022 [80]	For prediction of individual ICU patient's future ICP levels within each 10-min interval of the past hour, a probabilistic model was developed.	<u>*Subject Characteristics:*</u><br>Severe TBI patients<br>Number of subjects: 29<br>Subject demographics: 22 male/7 female<br>Age range: 20-80 years<br>Mean age: 56 years<br><br><u>*Experimental conditions:*</u><br>The patients who had pre-existing neurological disorders were excluded from the study. The patients were sedated and mechanically ventilated.	**Cerebral Physiology:**<br>ICP	1-min	Probabilistic Markov model and six different AR models	Probabilistic model and AR models had similar performance to an extent, however, probabilistic model had better performance at predicting dangerously high ICP values.	• The model was able to predict future ICP values of 20 mmHg or more with high specificity (0.94-0.95) and good to high sensitivity (0.73-0.87).<br>• Similar specificity (0.90-0.95) and sensitivity (0.73-0.89) values were obtained when leave-one-out cross-validation was applied, and the model was evaluated with individual patient data.<br>• Six different AR models were applied to six different intervals and achieved 0.84-0.98 specificity and 0.65-0.81 sensitivity, achieving similar performance as the probabilistic model to an extent.
Zeiler et al., 2018 [81]	For estimation of PRx using TCD based indices over a minute-by-minute interval, it was aimed to derive ARIMA based LME models generated for	<u>*Subject Characteristics:*</u><br>Mild to severe TBI patients<br>Number of subjects: 347<br>Subject demographics: 250 male/97 female<br>Mean age: 33.7±16.4 years	**Cerebral Physiology:**<br>ICP was monitored with an intraparenchymal strain gauge probe. CBFv was monitored with a TCD probe. DFv and MFv were calculated from CBFv. SFv was	10-sec	LME with ARIMA for Sx_a, Mx_a, and Dx_a models	PRx~Sx_a and PRx~Sx_a+ Mx_a LME models had the best performance among all generated ARIMA models	• Best correlation between estimated and observed PRx values were obtained by PRx~Sx_a LME model with a correlation of 0.794 (p-value<0.0001, CInt=0.788 to 0.799) and PRx~Sx_a+Mx_a LME model with a correlation of 0.814 (p-value<0.0001, CIint=0.809 to 0.819).<br>• It was concluded that PRx could be estimated by ARIMA based LME models using TCD based indices.

	each patient and for entire cohort.	*Experimental conditions:*<br>Data was extracted retrospectively from a database.	determined from maximum flow velocity.<br>(CPP = MABP - ICP)<br><br>*Other:*<br>ABP was recorded with pressure transducers through either radial or femoral arterial lines.			embedded in LME.	
Zeiler et al., 2019 [82]	PRx was aimed to be predicted using TCD based indices over a minute-by-minute interval with separate ARIMA based LME models generated for each patient.	*Subject Characteristics:*<br>Moderate to severe TBI patients<br>Number of subjects: 10<br>Subject demographics: 8 male/2 female<br>Mean age: 34.5±17.0 years<br><br>*Experimental conditions:*<br>Data was extracted retrospectively from a database. The patients depending on the severity of their TBI were intubated and sedated.	**Cerebral Physiology:**<br>ICP was monitored with an intraparenchymal strain gauge probe. CBFv was monitored with a TCD probe. SFv and MFv were calculated from CBFv. PRx was calculated from MABP and ICP. Mx_a was correlated from MFv and MABP. Sx_a was correlated from SFv and MABP.<br>(CPP = MABP – ICP)<br><br>*Other:*<br>ABP was recorded with pressure transducers through either radial or femoral arterial lines.	10-sec	LME with ARIMA model	PRx~Sx_a and PRx~Sx_a+ Mx_a LME models had the best performance among 16 generated ARIMA models embedded in LME.	• Strong correlation between the estimated and observed values of PRx was found with the LME models of PRx~Sx_a with a value of 0.998 (95% CInt = 0.990 – 0.999; p-value<0.0001) and PRx~Sx_a+Mx_a with a value of 0.997 (95% CInt = 0.988 – 0.999; p-value<0.0001) in the training set.<br>• Moderate correlation between the predicted and observed PRx values was also found with the PRx~Sx_a with a value of 0.797 (95% CInt = 0.336 – 0.949; p-value=0.006) and PRx~Sx_a+Mx_a with a value of 0.763 (95% CInt = 0.258 – 0.941; p-value=0.011) LME models with testing set.<br>• It was concluded that PRx prediction using TCD derived indices was attainable using ARIMA and LME modelling.
Zhang et al., 2011 [83]	Prediction of ICP on 15-min, 30-min and 45-min future intervals	*Subject Characteristics:*<br>Severe TBI patients<br>Number of subjects: 53	**Cerebral Physiology:**<br>ICP was measured with a fibre-optic	100 Hz	ANN_{NARX}-MFA, ANN_{NAR} and ARMA	ANN_{NARX}-MFA outperformed ANN_{NAR}	• It was observed that ANN_{NARX}-MFA showed the best performance in all future prediction intervals (R^2; $T_{15\text{-}min}$=0.93, $T_{30\text{-}min}$=0.81, $T_{45\text{-}min}$=0.56)

	were examined with a proposed ANN_{NARX}-MFA model and compared to ANN_{NAR} and ARMA models.	Subject demographics: 42 male/11 female<br><br>*Experimental conditions:*<br>Data was extracted retrospectively from a database.	intraparenchymal gauge			consistently in performance.	compared to ANN_{NAR} (R^2; $T_{15\text{-}min}$=0.88, $T_{30\text{-}min}$=0.73, $T_{45\text{-}min}$=0.43) and ARMA (R^2; $T_{15\text{-}min}$=0.76, $T_{30\text{-}min}$=0.61, $T_{45\text{-}min}$=0.28).
Zhang et al., 2012 [84]	For continuous trend prediction of ICP, an ARIMA method with orders selection predicted on PACF and ACF were examined and compared with ARIMA based on AIC and ANN.	*Subject Characteristics:*<br>TBI patients<br>Number of subjects: 27<br>Subject demographics: NA<br><br>*Experimental conditions:*<br>Data was extracted retrospectively from a database.	**Cerebral Physiology:**<br>ICP was measured with a fiber-optic intraparenchymal gauge.	1-sec	ARIMA based on PACF and ACF, ARIMA based on AIC, and ANN	Order selection predicted on PACF and ACF significantly improved the accuracy of ARIMA model with shorter prediction processing time compared to other two models.	• The accuracy of ARIMA based on PACF and ACF was higher (mean R^2=0.898) than ANN (mean R^2=0.804) and ARIMA based on AIC (mean R^2=0.712). ARIMA based on PACF and ACF also had shorter processing time for ICP forecasting.

ABP, arterial blood pressure; ACF, autocorrelation function; AdaBoost, adaptive boosting; AIC, Akaike information criterion; ANN, artificial neural network; ANN_{NARX}, nonlinear autoregressive with exogenous input artificial neural network, ANN_{NARX}-MFA, nonlinear autoregressive with exogenous input artificial neural network based mean forecast algorithm; AR, autoregressive; ARIMA, autoregressive integrative moving average; ARMA, autoregressive moving average; ARMAX, autoregressive moving average with exogenous input; AR-OR, autoregressive ordinal-regression; ARX, autoregressive with exogenous input; AS, arterial stenosis; AUC, area under the curve, AVM, arteriovenous malformations; BP, blood pressure; CA, cerebral autoregulation; CBFv, cerebral blood flow velocity; CInt, confidence interval; CPP, cerebral perfusion pressure; CRASH, corticosteroid randomisation after significant head injury; CWT, cross-wavelet transform; DEKF, dual extended Kalman filter; DFv, diastolic flow velocity; Dx_a, diastolic flow index; ECG, electrocardiography; EtCO2, end-tidal carbon dioxide; ETS, exponential smoothing; ExtraTrees, extremely randomized decision trees; GHW, generalized harmonic wavelets; GP, Gaussian processes; HF, high frequency; HR, heart rate; Hz, Hertz, ICP, intracranial pressure; ICU, intensive care unit; IMPACT, international mission for prognosis and clinical trial; IMPFA, intrinsic multiscale pressure-flow analysis; LET, Laguerre expansion technique; LF, low frequency; LGBM, light gradient boosting model; LME, linear mixed effects; MABP, mean arterial blood pressure; MCA, middle cerebral arterial; MDP, multiresolution dynamic predictor; MFv, mean flow velocity; MMPF, multimodal pressure-flow analysis; Mx_a, mean flow index; NA, not available; NMSE, normalized mean square error; PACF, partial autocorrelation function; $PbtO_2$, cerebral tissue oxygen; PCA, posterior cerebral artery; PCAv, posterior cerebral artery velocity; PRx, pressure reactivity index; R^2, coefficient of determination; RNN, recurrent neural network; RRSE, root relative squared error; SaO_2, arterial oxygen saturation; SFv, systolic flow velocity; STFT, short-time Fourier transform; SVM, support vector machine; Sx_a, systolic flow index; T, time; TBI, traumatic brain injury; TCD, transcranial Doppler; TFA, transfer function analysis; VLF, very low frequency; VAR, vector autoregressive; XGBoost; extreme gradient boosting

Table B8: Summary of studies for animal subjects

Article	Aim of the Study	Demographics & Experimental Conditions	Physiological Data & Measurement Methods	Data Resolution	Method(s) of Time-Series Modelling	Model Evaluation	Study Results and Conclusions Regarding Cerebral Physiologic Signal Modelling
Alexandrin, 2010 [85]	The myogenic response of pial arterioles in situ was examined during CBF autoregulation.	<u>*Subject Characteristics:*</u><br>Albino mature outbred male rats<br>Weight: 260-300 g<br>Number of subjects: NA<br><br><u>*Experimental conditions:*</u><br>The rats were narcotized with chloral hydrate, intraperitoneally. Both femoral arteries of the rats are isolated and catheterized after administration of heparin. Negative images were used to measure the inner diameter of the arterioles. Pial arteries through the dura matter were monitored.	**Cerebral Physiology:**<br>CBF were measured with a laser Doppler flowmetry before and after blood loss.<br><br>*Other:*<br>SBP, blood loss	600-sec	Wavelet analysis	No model performance was evaluated.	• It was observed the arterioles diameter significantly increased with drop in BP whereas a significant change in the relative tension of the vessel walls were observed only after systemic BP drop to 38 mmHg which resulted in failure of autoregulation of CBF.<br>• No change in myogenic component while the CBF oscillation increased with respect to decrease in the systemic BP with blood loss proved the effect of reduced perfusion pressure on the vessel wall tension.<br>• Wavelet analysis of CBF oscillation revealed an augmentation in oscillation amplitude while the myogenic response in autoregulation remained unaltered.<br>• The study results suggested that the myogenic reaction plays an important role in autoregulation of CBF.
Doblar et al., 1979 [86]	The impact of hypoxia on the dynamic characteristics of the cerebrovascular response was analyzed with Fourier analysis.	<u>*Subject Characteristics:*</u><br>Goats<br>Weight: 25-40 kg<br>Number of subjects: 3<br><br><u>*Experimental conditions:*</u><br>The goats were anesthetized and paralyzed with thiopental sodium and pancuronium bromide, respectively, and intubated using endotracheal tubes.<br>The applied stimuli frequencies ranged between 0.001 to 0.05 Hz.	**Cerebral Physiology:**<br>CBF was measured by an electromagnetic flow probe placed on the internal maxillary artery.<br><br>*Other:*<br>SaO_2 was measured by dual flow cuvette oximeter placed in	0.05 Hz	Fourier analysis	No model performance was evaluated.	• The findings suggested a possibility for some relation between phasic changes in blood pressure and phasic CBF response to hypoxia in mid-frequencies and no relation at lower and higher frequencies.<br>• Using normalized data, it was suggested that the cerebrovascular response to hypoxia could be considered a first-order function considering the limitations of the analysis.<br>• Results of the harmonic analysis suggested that at low and high frequencies, observed sinusoidal responses in CBF were unlikely to be result of the changes in blood pressure.<br>• It was concluded the main cause of the CBF response to sinusoidal hypoxia to be unrelated to the changes in blood pressure.

			arteriovenous shunt.				
Issam et al., 2019 [87]	The influence of the psychosomatic factor on the regulation of CBFv in response to emotional stress was analyzed.	*Subject Characteristics:*<br>Wistar male rats<br>Weight: 230-250 g<br>Number of subjects: 15<br><br>*Experimental conditions:*<br>The rats were anesthetized with isoflurane for the catheter implantation. A total of three catheters were implanted: one in the distal abdominal aorta and two in the inferior vena cava. Air stream was sent to the cage to generate emotional stress in the rats.	**Cerebral Physiology:**<br>CBFv was recorded with a TCD probe.<br><br>*Other:*<br>BP was measured by the arterial catheter. HR was calculated with FFT.	20 Hz	Cross-spectral analysis	No model performance was evaluated.	• The study results showed that the air jet causing high-intensity emotional stress significantly resulted in rising blood pressure and vasoconstriction of brain circulation.<br>• An increase in the variability of carotid blood flow and carotid vascular conductance during stress was observed with cross-spectral analysis compared to baseline state.<br>• It was concluded that the air-jet stress could cause hypertensive overload in the case of a failure in the baroreflex mechanism compromising the brain circulation mechanism and could consequently result in a stroke.
Zheng and Mayhew, 2009 [88]	The CBF-CBV coupling with respect to visco-elastic properties of the blood vessels was modelled.	*Subject Characteristics:*<br>Female hooded Lister rats<br>Weight: 250-400 g<br>Number of subjects: 5<br><br>*Experimental conditions:*<br>The rats were anesthetized with urethane and atropine. A slit spectrograph mounted camera and a laser-Doppler flowmeter probe were placed over the whisker barrel of a rat.	**Cerebral Physiology:**<br>CBF was measured by the laser Doppler flowmeter. CBV was obtained via optical imaging spectroscopy.	7.5 Hz	VW model and EW model	The VW model outperformed the EW model at predicting the CBV time-series.	• The variation of the difference between time-series of CBF and CBV were captured by the values of the two compliance parameters in VW model.<br>• The VW model was shown to successfully simulate the relationship between the CBF and CBV time-series via a first order nonlinear time-invariant dynamic system.

BP, blood pressure; CBF, cerebral blood flow; CBV, cerebral blood volume; EW, elastic windkessel; FFT, fast Fourier transform; HR, heart rate; Hz, Hertz; NA, not available; SaO_2, arterial oxygen saturation; SBP, systemic blood pressure; TCD, transcranial Doppler; VW, visco-elastic windkessel

REFERENCES

1. Brown, C.M.; Dütsch, M.; Öhring, S.; Neundörfer, B.; Hilz, M.J. Cerebral Autoregulation Is Compromised during Simulated Fluctuations in Gravitational Stress. *Eur J Appl Physiol* **2004**, *91*, 279–286, doi:10.1007/s00421-003-0965-5.
2. Katsogridakis, E.; Simpson, D.M.; Bush, G.; Fan, L.; Birch, A.A.; Allen, R.; Potter, J.F.; Panerai, R.B. Revisiting the Frequency Domain: The Multiple and Partial Coherence of Cerebral Blood Flow Velocity in the Assessment of Dynamic Cerebral Autoregulation. *Physiol. Meas.* **2016**, *37*, 1056, doi:10.1088/0967-3334/37/7/1056.
3. Kuo, T.B.-J.; Chern, C.-M.; Sheng, W.-Y.; Wong, W.-J.; Hu, H.-H. Frequency Domain Analysis of Cerebral Blood Flow Velocity and Its Correlation with Arterial Blood Pressure. *J Cereb Blood Flow Metab* **1998**, *18*, 311–318, doi:10.1097/00004647-199803000-00010.
4. Peng, T.; Rowley, A.B.; Ainslie, P.N.; Poulin, M.J.; Payne, S.J. Multivariate System Identification for Cerebral Autoregulation. *Ann Biomed Eng* **2008**, *36*, 308–320, doi:10.1007/s10439-007-9412-9.
5. Ainslie, P.N.; Barach, A.; Murrell, C.; Hamlin, M.; Hellemans, J.; Ogoh, S. Alterations in Cerebral Autoregulation and Cerebral Blood Flow Velocity during Acute Hypoxia: Rest and Exercise. *American Journal of Physiology-Heart and Circulatory Physiology* **2007**, *292*, H976–H983, doi:10.1152/ajpheart.00639.2006.
6. Claassen, J.A.H.R.; Levine, B.D.; Zhang, R. Dynamic Cerebral Autoregulation during Repeated Squat-Stand Maneuvers. *Journal of Applied Physiology* **2009**, *106*, 153–160, doi:10.1152/japplphysiol.90822.2008.
7. Iwasaki, K.; Ogawa, Y.; Shibata, S.; Aoki, K. Acute Exposure to Normobaric Mild Hypoxia Alters Dynamic Relationships between Blood Pressure and Cerebral Blood Flow at Very Low Frequency. *J Cereb Blood Flow Metab* **2007**, *27*, 776–784, doi:10.1038/sj.jcbfm.9600384.
8. Oudegeest-Sander, M.H.; van Beek, A.H.E.A.; Abbink, K.; Olde Rikkert, M.G.M.; Hopman, M.T.E.; Claassen, J.A.H.R. Assessment of Dynamic Cerebral Autoregulation and Cerebrovascular CO2 Reactivity in Ageing by Measurements of Cerebral Blood Flow and Cortical Oxygenation. *Experimental Physiology* **2014**, *99*, 586–598, doi:10.1113/expphysiol.2013.076455.
9. Panerai, R.B.; Haunton, V.J.; Llwyd, O.; Minhas, J.S.; Katsogridakis, E.; Salinet, A.S.; Maggio, P.; Robinson, T.G. Cerebral Critical Closing Pressure and Resistance-Area Product: The Influence of Dynamic Cerebral Autoregulation, Age and Sex. *J Cereb Blood Flow Metab* **2021**, *41*, 2456–2469, doi:10.1177/0271678X211004131.
10. Smirl, J.D.; Haykowsky, M.J.; Nelson, M.D.; Tzeng, Y.-C.; Marsden, K.R.; Jones, H.; Ainslie, P.N. Relationship Between Cerebral Blood Flow and Blood Pressure in Long-Term Heart Transplant Recipients. *Hypertension* **2014**, *64*, 1314–1320, doi:10.1161/HYPERTENSIONAHA.114.04236.
11. Zhang, R.; Zuckerman, J.H.; Giller, C.A.; Levine, B.D. Transfer Function Analysis of Dynamic Cerebral Autoregulation in Humans. *American Journal of Physiology-Heart and Circulatory Physiology* **1998**, *274*, H233–H241, doi:10.1152/ajpheart.1998.274.1.H233.
12. Addison, P.S. Identifying Stable Phase Coupling Associated with Cerebral Autoregulation Using the Synchrosqueezed Cross-Wavelet Transform and Low Oscillation Morlet Wavelets. In Proceedings of the 2015 37th Annual International Conference of the IEEE Engineering in Medicine and Biology Society (EMBC); August 2015; pp. 5960–5963.
13. Bu, L.; Li, J.; Li, F.; Liu, H.; Li, Z. Wavelet Coherence Analysis of Cerebral Oxygenation Signals Measured by Near-Infrared Spectroscopy in Sailors: An Exploratory, Experimental Study. *BMJ Open* **2016**, *6*, e013357, doi:10.1136/bmjopen-2016-013357.
14. Bu, L.; Zhang, M.; Li, J.; Li, F.; Liu, H.; Li, Z. Effects of Sleep Deprivation on Phase Synchronization as Assessed by Wavelet Phase Coherence Analysis of Prefrontal Tissue Oxyhemoglobin Signals. *PLOS ONE* **2017**, *12*, e0169279, doi:10.1371/journal.pone.0169279.
15. Bu, L.; Wang, D.; Huo, C.; Xu, G.; Li, Z.; Li, J. Effects of Poor Sleep Quality on Brain Functional Connectivity Revealed by Wavelet-Based Coherence Analysis Using NIRS Methods in Elderly Subjects. *Neuroscience Letters* **2018**, *668*, 108–114, doi:10.1016/j.neulet.2018.01.026.
16. Cui, R.; Zhang, M.; Li, Z.; Xin, Q.; Lu, L.; Zhou, W.; Han, Q.; Gao, Y. Wavelet Coherence Analysis of Spontaneous Oscillations in Cerebral Tissue Oxyhemoglobin Concentrations and Arterial Blood Pressure in Elderly Subjects. *Microvascular Research* **2014**, *93*, 14–20, doi:10.1016/j.mvr.2014.02.008.
17. Li, Z.; Zhang, M.; Cui, R.; Xin, Q.; Liqian, L.; Zhou, W.; Han, Q.; Gao, Y. Wavelet Coherence Analysis of Prefrontal Oxygenation Signals in Elderly Subjects with Hypertension. *Physiol. Meas.* **2014**, *35*, 777, doi:10.1088/0967-3334/35/5/777.

18. Saleem, S.; Teal, P.D.; Kleijn, W.B.; Ainslie, P.N.; Tzeng, Y.-C. Identification of Human Sympathetic Neurovascular Control Using Multivariate Wavelet Decomposition Analysis. *American Journal of Physiology-Heart and Circulatory Physiology* **2016**, *311*, H837–H848, doi:10.1152/ajpheart.00254.2016.
19. Tan, Q.; Zhang, M.; Wang, Y.; Zhang, M.; Wang, B.; Xin, Q.; Li, Z. Age-Related Alterations in Phase Synchronization of Oxyhemoglobin Concentration Changes in Prefrontal Tissues as Measured by near-Infrared Spectroscopy Signals. *Microvascular Research* **2016**, *103*, 19–25, doi:10.1016/j.mvr.2015.10.002.
20. Wang, B.; Zhang, M.; Bu, L.; Xu, L.; Wang, W.; Li, Z. Posture-Related Changes in Brain Functional Connectivity as Assessed by Wavelet Phase Coherence of NIRS Signals in Elderly Subjects. *Behavioural Brain Research* **2016**, *312*, 238–245, doi:10.1016/j.bbr.2016.06.037.
21. Clough, R.H.; Minhas, J.S.; Haunton, V.J.; Hanby, M.F.; Robinson, T.G.; Panerai, R.B. Dynamics of the Cerebral Autoregulatory Response to Paced Hyperventilation Assessed Using Subcomponent and Time-Varying Analyses. *Journal of Applied Physiology* **2022**, *133*, 311–319, doi:10.1152/japplphysiol.00100.2022.
22. Edwards, M.R.; Devitt, D.L.; Hughson, R.L. Two-Breath CO2 Test Detects Altered Dynamic Cerebrovascular Autoregulation and CO2 Responsiveness with Changes in Arterial Pco2. *American Journal of Physiology-Regulatory, Integrative and Comparative Physiology* **2004**, *287*, R627–R632, doi:10.1152/ajpregu.00384.2003.
23. Panerai, R.B.; Salinet, A.S.M.; Robinson, T.G. Contribution of Arterial Blood Pressure and PaCO2 to the Cerebrovascular Responses to Motor Stimulation. *American Journal of Physiology-Heart and Circulatory Physiology* **2012**, *302*, H459–H466, doi:10.1152/ajpheart.00890.2011.
24. Gehalot, P.; Mathew, A.; Behbehani, K.; Zhang, R. Efficacy of Using Mean Arterial Blood Pressure Sequence for Linear Modeling of Cerebral Autoregulation. In Proceedings of the 2005 IEEE Engineering in Medicine and Biology 27th Annual Conference; January 2005; pp. 5619–5622.
25. Liu, Y.; Allen, R. Analysis of Dynamic Cerebral Autoregulation Using an ARX Model Based on Arterial Blood Pressure and Middle Cerebral Artery Velocity Simulation. *Med. Biol. Eng. Comput.* **2002**, *40*, 600–605, doi:10.1007/BF02345461.
26. Liu, Y.; Birch, A.A.; Allen, R. Dynamic Cerebral Autoregulation Assessment Using an ARX Model: Comparative Study Using Step Response and Phase Shift Analysis. *Medical Engineering & Physics* **2003**, *25*, 647–653, doi:10.1016/S1350-4533(03)00015-8.
27. Chacon, M.; Araya, C.; Panerai, R.B. Non-Linear Multivariate Modeling of Cerebral Hemodynamics with Autoregressive Support Vector Machines. *Medical Engineering & Physics* **2011**, *33*, 180–187, doi:10.1016/j.medengphy.2010.09.023.
28. Chacón, M.; Jara, J.L.; Miranda, R.; Katsogridakis, E.; Panerai, R.B. Non-Linear Models for the Detection of Impaired Cerebral Blood Flow Autoregulation. *PLOS ONE* **2018**, *13*, e0191825, doi:10.1371/journal.pone.0191825.
29. Chacón, M.; Rojas-Pescio, H.; Peñaloza, S.; Landerretche, J. Machine Learning Models and Statistical Complexity to Analyze the Effects of Posture on Cerebral Hemodynamics. *Entropy* **2022**, *24*, 428, doi:10.3390/e24030428.
30. Edwards, M.R.; Lin, D.C.; Hughson, R.L. Modeling the Interaction Between Perfusion Pressure and CO2 on Cerebral Blood Flow. In *Frontiers in Modeling and Control of Breathing: Integration at Molecular, Cellular, and Systems Levels*; Poon, C.-S., Kazemi, H., Eds.; Advances in Experimental Medicine and Biology; Springer US: Boston, MA, 2001; pp. 285–290 ISBN 978-1-4615-1375-9.
31. Kostoglou, K.; Debert, C.T.; Poulin, M.J.; Mitsis, G.D. Nonstationary Multivariate Modeling of Cerebral Autoregulation during Hypercapnia. *Medical Engineering & Physics* **2014**, *36*, 592–600, doi:10.1016/j.medengphy.2013.10.011.
32. Marmarelis, V.Z.; Shin, D.C.; Zhang, R. Linear and Nonlinear Modeling of Cerebral Flow Autoregulation Using Principal Dynamic Modes. *Open Biomedical Engineering Journal* **2012**, *6*, 42–55, doi:10.2174/1874230001206010042.
33. Marmarelis, V.Z.; Mitsis, G.D.; Shin, D.C.; Zhang, R. Multiple-Input Nonlinear Modelling of Cerebral Haemodynamics Using Spontaneous Arterial Blood Pressure, End-Tidal CO2 and Heart Rate Measurements. *Philosophical Transactions of the Royal Society A: Mathematical, Physical and Engineering Sciences* **2016**, *374*, 20150180, doi:10.1098/rsta.2015.0180.
34. Mitsis, G.D.; Poulin, M.J.; Robbins, P.A.; Marmarelis, V.Z. Nonlinear Modeling of the Dynamic Effects of Arterial Pressure and CO/Sub 2/ Variations on Cerebral Blood Flow in Healthy Humans. *IEEE Transactions on Biomedical Engineering* **2004**, *51*, 1932–1943, doi:10.1109/TBME.2004.834272.
35. Mitsis, G.D.; Zhang, R.; Levine, B.D.; Marmarelis, V.Z. Cerebral Hemodynamics during Orthostatic Stress Assessed by Nonlinear Modeling. *Journal of Applied Physiology* **2006**, *101*, 354–366, doi:10.1152/japplphysiol.00548.2005.

36. Panerai, R.B.; Dawson, S.L.; Potter, J.F. Linear and Nonlinear Analysis of Human Dynamic Cerebral Autoregulation. *American Journal of Physiology-Heart and Circulatory Physiology* **1999**, *277*, H1089–H1099, doi:10.1152/ajpheart.1999.277.3.H1089.
37. Panerai, R.B.; Chacon, M.; Pereira, R.; Evans, D.H. Neural Network Modelling of Dynamic Cerebral Autoregulation: Assessment and Comparison with Established Methods. *Medical Engineering & Physics* **2004**, *26*, 43–52, doi:10.1016/j.medengphy.2003.08.001.
38. Placek, M.M.; Wachel, P.; Iskander, D.R.; Smielewski, P.; Uryga, A.; Mielczarek, A.; Szczepański, T.A.; Kasprowicz, M. Applying Time-Frequency Analysis to Assess Cerebral Autoregulation during Hypercapnia. *PLOS ONE* **2017**, *12*, e0181851, doi:10.1371/journal.pone.0181851.
39. Czosnyka, M.; Guazzo, E.; Whitehouse, M.; Smielewski, P.; Czosnyka, Z.; Kirkpatrick, P.; Piechnik, S.; Pickard, J.D. Significance of Intracranial Pressure Waveform Analysis after Head Injury. *Acta neurochir* **1996**, *138*, 531–542, doi:10.1007/BF01411173.
40. Elixmann, I.M.; Hansinger, J.; Goffin, C.; Antes, S.; Radermacher, K.; Leonhardt, S. Single Pulse Analysis of Intracranial Pressure for a Hydrocephalus Implant. In Proceedings of the 2012 Annual International Conference of the IEEE Engineering in Medicine and Biology Society; August 2012; pp. 3939–3942.
41. Giller, C.; Gerardo lacopino, D. Use of Middle Cerebral Velocity and Blood Pressure for the Analysis of Cerebral Autoregulation at Various Frequencies: The Coherence Index. *Neurological Research* **1997**, *19*, 634–640, doi:10.1080/01616412.1997.11740873.
42. Li, W.; Zhang, M.; Huo, C.; Xu, G.; Chen, W.; Wang, D.; Li, Z. Time-Evolving Coupling Functions for Evaluating the Interaction between Cerebral Oxyhemoglobin and Arterial Blood Pressure with Hypertension. *Medical Physics* **2021**, *48*, 2027–2037, doi:10.1002/mp.14627.
43. Liu, Q.; Wang, B.; Liu, Y.; Lv, Z.; Li, W.; Li, Z.; Fan, Y. Frequency-Specific Effective Connectivity in Subjects with Cerebral Infarction as Revealed by NIRS Method. *Neuroscience* **2018**, *373*, 169–181, doi:10.1016/j.neuroscience.2018.01.007.
44. Martinez-Tejada, I.; Czosnyka, M.; Czosnyka, Z.; Juhler, M.; Smielewski, P. Causal Relationship between Slow Waves of Arterial, Intracranial Pressures and Blood Velocity in Brain. *Computers in Biology and Medicine* **2021**, *139*, 104970, doi:10.1016/j.compbiomed.2021.104970.
45. Caldas, J.R.; Panerai, R.B.; Bor-Seng-Shu, E.; Almeida, J.P.; Ferreira, G.S.R.; Camara, L.; Nogueira, R.C.; Oliveira, M.L.; Jatene, F.B.; Robinson, T.G.; et al. Cerebral Hemodynamics with Intra-Aortic Balloon Pump: Business as Usual? *Physiol. Meas.* **2017**, *38*, 1349, doi:10.1088/1361-6579/aa68c4.
46. Haubrich, C.; Diehl, R.R.; Kasprowicz, M.; Diedler, J.; Sorrentino, E.; Smielewski, P.; Czosnyka, M. Increasing Intracranial Pressure After Head Injury: Impact on Respiratory Oscillations in Cerebral Blood Flow Velocity. In *Intracranial Pressure and Brain Monitoring XV*; Ang, B.-T., Ed.; Acta Neurochirurgica Supplement; Springer International Publishing: Cham, 2016; pp. 171–175 ISBN 978-3-319-22533-3.
47. Panerai, R.B.; Rennie, J.M.; Kelsall, A.W.R.; Evans, D.H. Frequency-Domain Analysis of Cerebral Autoregulation from Spontaneous Fluctuations in Arterial Blood Pressure. *Med. Biol. Eng. Comput.* **1998**, *36*, 315–322, doi:10.1007/BF02522477.
48. Sammons, E.L.; Samani, N.J.; Smith, S.M.; Rathbone, W.E.; Bentley, S.; Potter, J.F.; Panerai, R.B. Influence of Noninvasive Peripheral Arterial Blood Pressure Measurements on Assessment of Dynamic Cerebral Autoregulation. *Journal of Applied Physiology* **2007**, *103*, 369–375, doi:10.1152/japplphysiol.00271.2007.
49. Han, Q.; Zhang, M.; Li, W.; Gao, Y.; Xin, Q.; Wang, Y.; Li, Z. Wavelet Coherence Analysis of Prefrontal Tissue Oxyhaemoglobin Signals as Measured Using Near-Infrared Spectroscopy in Elderly Subjects with Cerebral Infarction. *Microvascular Research* **2014**, *95*, 108–115, doi:10.1016/j.mvr.2014.08.001.
50. Kvandal, P.; Sheppard, L.; Landsverk, S.A.; Stefanovska, A.; Kirkeboen, K.A. Impaired Cerebrovascular Reactivity after Acute Traumatic Brain Injury Can Be Detected by Wavelet Phase Coherence Analysis of the Intracranial and Arterial Blood Pressure Signals. *J Clin Monit Comput* **2013**, *27*, 375–383, doi:10.1007/s10877-013-9484-z.
51. Tian, F.; Tarumi, T.; Liu, H.; Zhang, R.; Chalak, L. Wavelet Coherence Analysis of Dynamic Cerebral Autoregulation in Neonatal Hypoxic–Ischemic Encephalopathy. *NeuroImage: Clinical* **2016**, *11*, 124–132, doi:10.1016/j.nicl.2016.01.020.
52. Turalska, M.; Latka, M.; Czosnyka, M.; Pierzchala, K.; West, B.J. Generation of Very Low Frequency Cerebral Blood Flow Fluctuations in Humans. In Proceedings of the Acta Neurochirurgica Supplements; Steiger, H.-J., Ed.; Springer: Vienna, 2009; pp. 43–47.
53. Daley, M.L.; Leffler, C.W.; Czosnyka, M.; Pickard, J.D. Intracranial Pressure Monitoring: Modeling Cerebrovascular Pressure Transmission. In Proceedings of the Brain Edema XIII; Hoff, J.T., Keep, R.F., Xi, G., Hua, Y., Eds.; Springer: Vienna, 2006; pp. 103–107.

54. Pinto, H.; Dias, C.; Rocha, A.P. Multiscale Information Decomposition of Long Memory Processes: Application to Plateau Waves of Intracranial Pressure. In Proceedings of the 2022 44th Annual International Conference of the IEEE Engineering in Medicine & Biology Society (EMBC); July 2022; pp. 1753–1756.
55. Thelin, E.P.; Raj, R.; Bellander, B.-M.; Nelson, D.; Piippo-Karjalainen, A.; Siironen, J.; Tanskanen, P.; Hawryluk, G.; Hasen, M.; Unger, B.; et al. Comparison of High versus Low Frequency Cerebral Physiology for Cerebrovascular Reactivity Assessment in Traumatic Brain Injury: A Multi-Center Pilot Study. *J Clin Monit Comput* **2020**, *34*, 971–994, doi:10.1007/s10877-019-00392-y.
56. Zeiler, F.A.; Aries, M.; Cabeleira, M.; van Essen, T.A.; Stocchetti, N.; Menon, D.K.; Timofeev, I.; Czosnyka, M.; Smielewski, P.; Hutchinson, P.; et al. Statistical Cerebrovascular Reactivity Signal Properties after Secondary Decompressive Craniectomy in Traumatic Brain Injury: A CENTER-TBI Pilot Analysis. *Journal of Neurotrauma* **2020**, *37*, 1306–1314, doi:10.1089/neu.2019.6726.
57. Zeiler, F.A.; Cabeleira, M.; Hutchinson, P.J.; Stocchetti, N.; Czosnyka, M.; Smielewski, P.; Ercole, A.; Anke, A.; Beer, R.; Bellander, B.-M.; et al. Evaluation of the Relationship between Slow-Waves of Intracranial Pressure, Mean Arterial Pressure and Brain Tissue Oxygen in TBI: A CENTER-TBI Exploratory Analysis. *J Clin Monit Comput* **2021**, *35*, 711–722, doi:10.1007/s10877-020-00527-6.
58. Asgari, S.; Adams, H.; Kasprowicz, M.; Czosnyka, M.; Smielewski, P.; Ercole, A. Feasibility of Hidden Markov Models for the Description of Time-Varying Physiologic State After Severe Traumatic Brain Injury. *Critical Care Medicine* **2019**, *47*, e880, doi:10.1097/CCM.0000000000003966.
59. Chiu, C.-C.; Yeh, S.-J.; Li, T.-Y. Classification of Diabetics with Various Degrees of Autonomic Neuropathy Based on Linear and Nonlinear Features Using Support Vector Machine. In Proceedings of the Medical Biometrics; Zhang, D., Sonka, M., Eds.; Springer: Berlin, Heidelberg, 2010; pp. 42–51.
60. Mariak, Z.; Swiercz, M.; Krejza, J.; Lewko, J.; Lyson, T. Intracranial Pressure Processing with Artificial Neural Networks: Classification of Signal Properties. *Acta Neurochir (Wien)* **2000**, *142*, 407–412, doi:10.1007/s007010050450.
61. Megjhani, M.; Weiss, M.; Kwon, S.B.; Ford, J.; Nametz, D.; Kastenholz, N.; Fogel, H.; Velazquez, A.; Roh, D.; Agarwal, S.; et al. Vector Angle Analysis of Multimodal Neuromonitoring Data for Continuous Prediction of Delayed Cerebral Ischemia. *Neurocrit Care* **2022**, *37*, 230–236, doi:10.1007/s12028-022-01481-8.
62. Naraei, P.; Kenez, M.; Sadeghian, A. A Hybrid Wavelet Based K-Means Clustering Approach to Detect Intracranial Hypertension. In Proceedings of the 2017 IEEE Canada International Humanitarian Technology Conference (IHTC); July 2017; pp. 21–25.
63. Porta, A.; Fantinato, A.; Bari, V.; Gelpi, F.; Cairo, B.; Maria, B.D.; Bertoldo, E.G.; Fiolo, V.; Callus, E.; Vincentiis, C.D.; et al. Evaluation of the Impact of Surgical Aortic Valve Replacement on Short-Term Cardiovascular and Cerebrovascular Controls through Spontaneous Variability Analysis. *PLOS ONE* **2020**, *15*, e0243869, doi:10.1371/journal.pone.0243869.
64. Shaw, M.; Hawthorne, C.; Moss, L.; Kommer, M.; O'Kane, R.; Piper, I. Time Series Analysis and Prediction of Intracranial Pressure Using Time-Varying Dynamic Linear Models. In *Intracranial Pressure and Neuromonitoring XVII*; Depreitere, B., Meyfroidt, G., Güiza, F., Eds.; Acta Neurochirurgica Supplement; Springer International Publishing: Cham, 2021; pp. 225–229 ISBN 978-3-030-59436-7.
65. Sourina, O.; Ang, B.-T.; Nguyen, M.K. Fractal-Based Approach in Analysis of Intracranial Pressure (ICP) in Severe Head Injury. In Proceedings of the Proceedings of the 10th IEEE International Conference on Information Technology and Applications in Biomedicine; November 2010; pp. 1–4.
66. Farhadi, A.; Chern, J.J.; Hirsh, D.; Davis, T.; Jo, M.; Maier, F.; Rasheed, K. Intracranial Pressure Forecasting in Children Using Dynamic Averaging of Time Series Data. *Forecasting* **2019**, *1*, 47–58, doi:10.3390/forecast1010004.
67. Güiza, F.; Depreitere, B.; Piper, I.; Van den Berghe, G.; Meyfroidt, G. Novel Methods to Predict Increased Intracranial Pressure During Intensive Care and Long-Term Neurologic Outcome After Traumatic Brain Injury: Development and Validation in a Multicenter Dataset*. *Critical Care Medicine* **2013**, *41*, 554, doi:10.1097/CCM.0b013e3182742d0a.
68. Hu, K.; Lo, M.-T.; Peng, C.-K.; Liu, Y.; Novak, V. A Nonlinear Dynamic Approach Reveals a Long-Term Stroke Effect on Cerebral Blood Flow Regulation at Multiple Time Scales. *PLOS Computational Biology* **2012**, *8*, e1002601, doi:10.1371/journal.pcbi.1002601.
69. Jachan, M.; Reinhard, M.; Spindeler, L.; Hetzel, A.; Schelter, B.; Timmer, J. Parametric Versus Nonparametric Transfer Function Estimation of Cerebral Autoregulation from Spontaneous Blood-Pressure Oscillations. *Cardiovasc Eng* **2009**, *9*, 72–82, doi:10.1007/s10558-009-9072-5.

70. Kostoglou, K.; Wright, A.D.; Smirl, J.D.; Bryk, K.; van Donkelaar, P.; Mitsis, G.D. Dynamic Cerebral Autoregulation in Young Athletes Following Concussion. In Proceedings of the 2016 38th Annual International Conference of the IEEE Engineering in Medicine and Biology Society (EMBC); August 2016; pp. 696–699.
71. Miller, E.C.; Santos, K.R.M. dos; Marshall, R.S.; Kougioumtzoglou, I.A. Joint Time-Frequency Analysis of Dynamic Cerebral Autoregulation Using Generalized Harmonic Wavelets. *Physiol. Meas.* **2020**, *41*, 024002, doi:10.1088/1361-6579/ab71f2.
72. Myers, R.B.; Lazaridis, C.; Jermaine, C.M.; Robertson, C.S.; Rusin, C.G. Predicting Intracranial Pressure and Brain Tissue Oxygen Crises in Patients With Severe Traumatic Brain Injury. *Critical Care Medicine* **2016**, *44*, 1754, doi:10.1097/CCM.0000000000001838.
73. Petrov, D.; Miranda, S.P.; Balu, R.; Wathen, C.; Vaz, A.; Mohan, V.; Colon, C.; Diaz-Arrastia, R. Prediction of Intracranial Pressure Crises after Severe Traumatic Brain Injury Using Machine Learning Algorithms. *Journal of Neurosurgery* **2023**, *1*, 1–8, doi:10.3171/2022.12.JNS221860.
74. Scalzo, F.; Hamilton, R.; Asgari, S.; Kim, S.; Hu, X. Intracranial Hypertension Prediction Using Extremely Randomized Decision Trees. *Medical Engineering & Physics* **2012**, *34*, 1058–1065, doi:10.1016/j.medengphy.2011.11.010.
75. Schäck, T.; Muma, M.; Feng, M.; Guan, C.; Zoubir, A.M. Robust Nonlinear Causality Analysis of Nonstationary Multivariate Physiological Time Series. *IEEE Transactions on Biomedical Engineering* **2018**, *65*, 1213–1225, doi:10.1109/TBME.2017.2708609.
76. Semenyutin, V.; Antonov, V.; Malykhina, G.; Salnikov, V. Investigation of Cerebral Autoregulation Using Time-Frequency Transformations. *Biomedicines* **2022**, *10*, 3057, doi:10.3390/biomedicines10123057.
77. Swiercz, M.; Mariak, Z.; Lewko, J.; Chojnacki, K.; Kozlowski, A.; Piekarski, P. Neural Network Technique for Detecting Emergency States in Neurosurgical Patients. *Med. Biol. Eng. Comput.* **1998**, *36*, 717–722, doi:10.1007/BF02518874.
78. Swiercz, M.; Mariak, Z.; Krejza, J.; Lewko, J.; Szydlik, P. Intracranial Pressure Processing with Artificial Neural Networks: Prediction of ICP Trends. *Acta Neurochir (Wien)* **2000**, *142*, 401–406, doi:10.1007/s007010050449.
79. Tsui, F.-C.; Sun, M.; Li, C.-C.; Sclabassi, R.J. A Wavelet Based Neural Network for Prediction of ICP Signal. In Proceedings of the Proceedings of 17th International Conference of the Engineering in Medicine and Biology Society; September 1995; Vol. 2, pp. 1045–1046 vol.2.
80. Wijayatunga, P.; Koskinen, L.-O.D.; Sundström, N. Probabilistic Prediction of Increased Intracranial Pressure in Patients with Severe Traumatic Brain Injury. *Sci Rep* **2022**, *12*, 9600, doi:10.1038/s41598-022-13732-x.
81. Zeiler, F.A.; Smielewski, P.; Donnelly, J.; Czosnyka, M.; Menon, D.K.; Ercole, A. Estimating Pressure Reactivity Using Noninvasive Doppler-Based Systolic Flow Index. *Journal of Neurotrauma* **2018**, *35*, 1559–1568, doi:10.1089/neu.2017.5596.
82. Zeiler, F.A.; Smielewski, P.; Stevens, A.; Czosnyka, M.; Menon, D.K.; Ercole, A. Non-Invasive Pressure Reactivity Index Using Doppler Systolic Flow Parameters: A Pilot Analysis. *Journal of Neurotrauma* **2019**, *36*, 713–720, doi:10.1089/neu.2018.5987.
83. Zhang, F.; Feng, M.; Pan, S.J.; Loy, L.Y.; Guo, W.; Zhang, Z.; Chin, P.L.; Guan, C.; King, N.K.K.; Ang, B.T. Artificial Neural Network Based Intracranial Pressure Mean Forecast Algorithm for Medical Decision Support. In Proceedings of the 2011 Annual International Conference of the IEEE Engineering in Medicine and Biology Society; August 2011; pp. 7111–7114.
84. Zhang, F.; Feng, M.; Loy, L.Y.; Zhang, Z.; Guan, C. Online ICP Forecast for Patients with Traumatic Brain Injury. In Proceedings of the Proceedings of the 21st International Conference on Pattern Recognition (ICPR2012); November 2012; pp. 37–40.
85. Alexandrin, V.V. Relationship between Myogenic Reaction and Autoregulation of Cerebral Circulation. *Bull Exp Biol Med* **2010**, *150*, 168–171, doi:10.1007/s10517-010-1095-9.
86. Doblar, D.D.; Min, B.G.; Chapman, R.W.; Harback, E.R.; Welkowitz, W.; Edelman, N.H. Dynamic Characteristics of Cerebral Blood Flow Response to Sinusoidal Hypoxia. *Journal of Applied Physiology* **1979**, *46*, 721–729, doi:10.1152/jappl.1979.46.4.721.
87. Issam, N.; Raffaello, S.; Dafne, S.; Luigi, C.; Abdelkrim, T. A Simple Approach to Studying Cerebral Blood Flow during Psychological Stress. *Naunyn-Schmiedeberg's Arch Pharmacol* **2019**, *392*, 505–509, doi:10.1007/s00210-019-01638-x.
88. Zheng, Y.; Mayhew, J. A Time-Invariant Visco-Elastic Windkessel Model Relating Blood Flow and Blood Volume. *NeuroImage* **2009**, *47*, 1371–1380, doi:10.1016/j.neuroimage.2009.04.022.
